# Superelectrophilic Activation of Phosphacoumarins towards Weak Nucleophiles via Brønsted Acid Assisted Brønsted Acid Catalysis

**DOI:** 10.3390/ijms25126327

**Published:** 2024-06-07

**Authors:** Alena V. Zalaltdinova, Yulia M. Sadykova, Almir S. Gazizov, Atabek K. Smailov, Victor V. Syakaev, Daria P. Gerasimova, Elena A. Chugunova, Nurgali I. Akylbekov, Rakhmetulla U. Zhapparbergenov, Nurbol O. Appazov, Alexander R. Burilov, Michail A. Pudovik, Igor V. Alabugin, Oleg G. Sinyashin

**Affiliations:** 1 FRC Kazan Scientific Center, Arbuzov Institute of Organic and Physical Chemistry, Russian Academy of Science, Arbuzova Str., 8, 420088 Kazan, Russia; zalaltdinova.alena@iopc.ru (A.V.Z.); vvs@iopc.ru (V.V.S.); darya.p_gerasimova@mail.ru (D.P.G.); burilov@iopc.ru (A.R.B.);; 2 Kazan National Research Technological University, Karl Marx Str., 68, 420015 Kazan, Russia; 3 Laboratory of Engineering Profile “Physical and Chemical Methods of Analysis”, Korkyt Ata Kyzylorda University, Aitekebie Str., 29A, Kyzylorda 120014, Kazakhstan; nurgali_089@mail.ru (N.I.A.);; 4 “CNEC” LLP, Dariger Ali Str., Kyzylorda 120001, Kazakhstan; 5 “DPS Kyzylorda” LLP, Amangeldy Imanov Str., 112A, Kyzylorda 120008, Kazakhstan; 6 Department of Chemistry and Biochemistry, Florida State University, Tallahassee, FL 32306, USA

**Keywords:** phosphacoumarins, acid catalysis, arenes, electrophilicity

## Abstract

The electrophilic activation of various substrates via double or even triple protonation in superacidic media enables reactions with extremely weak nucleophiles. Despite the significant progress in this area, the utility of organophosphorus compounds as superelectrophiles still remains limited. Additionally, the most common superacids require a special care due to their high toxicity, exceptional corrosiveness and moisture sensitivity. Herein, we report the first successful application of the “Brønsted acid assisted Brønsted acid” concept for the superelectrophilic activation of 2-hydroxybenzo[*e*][1,2]oxaphosphinine 2-oxides (phosphacoumarins). The pivotal role is attributed to the tendency of the phosphoryl moiety to form hydrogen-bonded complexes, which enables the formation of dicationic species and increases the electrophilicity of the phosphacoumarin. This unmasks the reactivity of phosphacoumarins towards non-activated aromatics, while requiring only relatively non-benign trifluoroacetic acid as the reaction medium.

## 1. Introduction

The concept of superelectrophilic activation was first introduced by Staskun in 1964 to explain the mechanism of the acid-promoted cyclizations of β-ketoamides [1]. Later, it was popularized by Olah in the classic studies of stable carbocations [2] and heteroatom-containing ions [3] in highly acidic media. Since these pioneering studies, significant progress has been achieved in this area. Various synthetic methodologies have been developed, which utilize these superelectrophilic species [4]. A number of reviews summarize various aspects of superelectrophile chemistry [5,6,7,8] and the closely related chemistry of superacids [9].

**Scheme 1:**
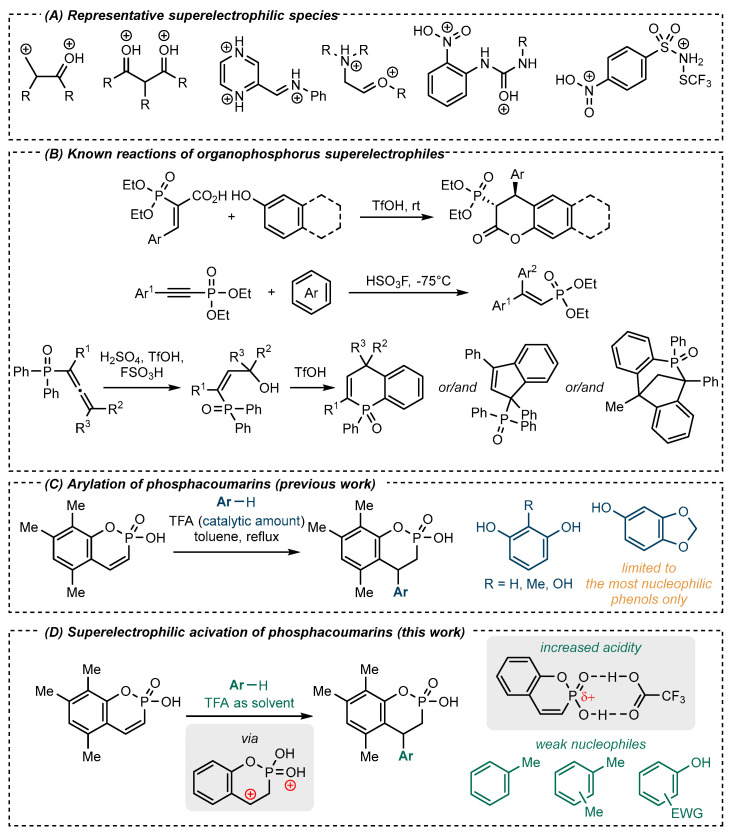
Some examples of common superelectrophilic species [10,11,12,13,14,15,16,17,18,19] (**A**), the reactions of organophosphorus superelectrophiles reported thus far [20,21,22,23,24,25,26,27] (**B**), the known arylation of phosphacoumarins (**C**) and the superelectrophilic activation of phosphacoumarins reported in this work (**D**).

The concept of superelectrophilic activation is often associated with the formation of doubly protonated dicationic species in strongly acidic (superacidic) media, which are capable of reacting with exceptionally weak nucleophiles. Some examples of dicationic superelectrophiles are presented in Scheme 1A. The generation of superelectrophilic species may be achieved via the diprotonation of α,β-unsaturated carbonyl compounds [10,11], 1,2- [12,13] and 1,3-dicarbonyls [14] and related compounds. This methodology has also been extended to other groups capable of being protonated in acidic media, such as nitrogen-containing heterocycles [15], aminoacetals [16,17] and even nitroarylureas [18] and nitroarylsulfamides [19]. Despite the growing number of compounds employed as precursors of superelectrophiles, the superelectrophilic activation of organophosphorus compounds has received little attention and examples of such reactions are still rare (Scheme 1B). In particular, Krawczyk and coworkers reported the triflic acid-catalyzed condensation of 2-(diethoxyphosphoryl)acrylic acid derivatives with electron-rich hydroxyarenes [20]. The reactions of vinyl type carbocations derived from ethynylphosphonates with substituted benzenes in fluorosulfonic acid have been described by Vasil’ev and coworkers [21,22]. Later, the same group reported a series of transformations of 1-(phosphoryl)allenes in superacidic media, which furnished various phosphorus heterocycles [23,24,25,26,27]. Thus far, these are the only known reactions of organophosphorus superelectrophiles.

Generally, dicationic intermediates are generated in strong acids, with trifluoromethanesulfonic (triflic) [28], fluoroantimonic [29] and “magic acid” (HSO_3_F/SbF_5_) being the representative examples. Other prominent examples of superacids include the carborane acids reported by Reed and coworkers [30,31,32], which are presumably the strongest acids obtained thus far. In spite of being a versatile tool in organic chemistry, traditional superacids are not free from drawbacks. The toxicity of the reagents (e.g., HF and SbF_5_), their highly corrosive nature and sensitivity to water and residual moisture, as well their cost, somewhat limit their application in daily lab work.

Meanwhile, an acid-assisted acid catalysis, particularly Brønsted acid assisted Brønsted acid catalysis (BBA), has emerged as a useful tool for the design of highly reactive and selective acidic catalysts [33,34]. Earlier, we reported the acid-catalyzed arylation of 2-hydroxybenzo[*e*][1,2]oxaphosphinine 2-oxides (phosphacoumarins) with some electron-rich phenols [35]. However, this transformation was limited to the most reactive phenols only, other aromatics being inert under the reported conditions (Scheme 1C). We speculated that the BBA concept could be applied to enhance the reactivity of phosphacoumarins, thus expanding the scope of suitable substrates. As a result of our studies, herein we report the trifluoroacetic acid (TFA)-assisted arylation of phosphacoumarins, where phosphacoumarin acts both as the substrate and the Bronsted acid and TFA serves as a reaction media. The key to the successful reaction is the strong hydrogen-bonding ability of the phosphoryl group, which facilitates its complexation with TFA. This increases the acidity of the phosphacoumarin’s phosphonic acid moiety and simultaneously enables the formation of the superelectrophilic species. As a result of this dual activation, even weak nucleophiles can be subjected to the reaction in mild conditions (Scheme 1D).

## 2. Results and Discussion

### 2.1. Synthesis of 4-(aryl)phosphaflavanoids in TFA Medium

**Scheme 2:**
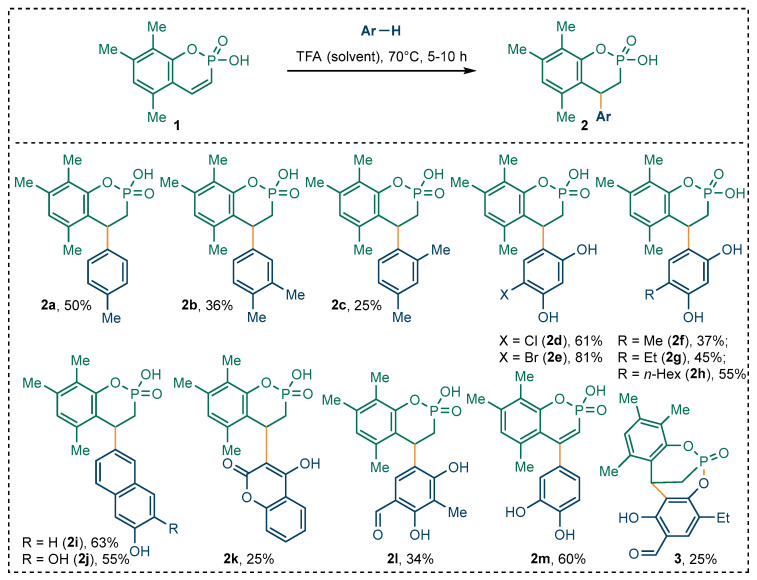
Synthesis of 4-aryl-substituted phosphaflavanoids in TFA medium.

We initiated our studies by carrying out the reaction of the phosphacoumarin **1** with toluene using TFA as a solvent. The reaction did not proceed at room temperature regardless of the reagents’ ratio. Pleasingly, simply by increasing the temperature up to 70 °C the equimolar mixture of toluene and the phosphacoumarin **1** provided the mixture of *o*- and *p*-arylated products, from which the *p*-isomer **2a** was isolated in a 50% yield (Scheme 2). Benzene, a less nucleophilic substrate, proved to be unreactive under these conditions, whereas *o*- and *m*-xylenes furnished the target compounds **2b** and **2c** in 36% and 24% isolated yields, accordingly. The structures of the compounds **2b** and **2c** were elucidated using X-ray diffractometry (see Supplementary Materials for the detailed X-ray data). Encouraged by these “proof-of-concept” reactions, we next switched to other arenes, which were unreactive towards phosphacoumarins in previous studies.

Notably, the 4-(hydroxyarene)-substituted coumarin scaffold is present in many natural biologically active compounds, e.g., flavonoids [36,37,38]. The replacement of a particular fragment of a natural compound by a phosphorus-containing moiety can provide an entry to novel biologically active scaffolds. The remarkable example of this approach is the replacement of a carboxyl group by a phosphoryl group in amino acids. Thus-derived aminophosphonates are well-known bioactive compounds and have found widespread applications in medicinal chemistry [39,40,41]. With this in mind, we aimed at the synthesis of phosphaflavanoids possessing a hydroxyarene moiety. In contrast to the previously reported procedure [35], various phenols with electron-withdrawing groups reacted in TFA media with the compound **1** to give the desired phosphorus analogues of flavonoids **2d**,**e** and **2l**. Isolated yields of the compound **2l** were considerably lower compared to the phosphaflavanoids **2d**,**e**, which is in accordance with the stronger electron-withdrawing nature of the formyl group. The yields of the alkyl-substituted derivatives **2f**–**h** were lower compared to the other compounds. However, based on the ^31^P NMR spectra of the reaction mixtures, we attribute this to their higher solubility in organic solvents, which causes significant losses during purification, rather than to the electronic effects of the substituents. The same holds true for the compound **2k** with the 4-hydrocoumarin moiety, which could be isolated in a 25% yield only, despite the ^31^P NMR spectrum of the reaction mixture evidencing its almost quantitative yield.

Interestingly, the reaction of the phosphacoumarin **1** with 5-ethyl-2,4-dihydroxybenzaldehyde yielded the bicyclic phosphonate **3** instead of the expected phosphaflavanoid. The structure of the obtained compound was confirmed by X-ray analysis (see the Supporting Materials for the detailed X-ray data). Presumably, the compound **3** is formed via an intermediate phosphaflavanoid, which further undergoes intramolecular cyclisation to give the final product, as was described by us earlier [42].

**Figure 1:**
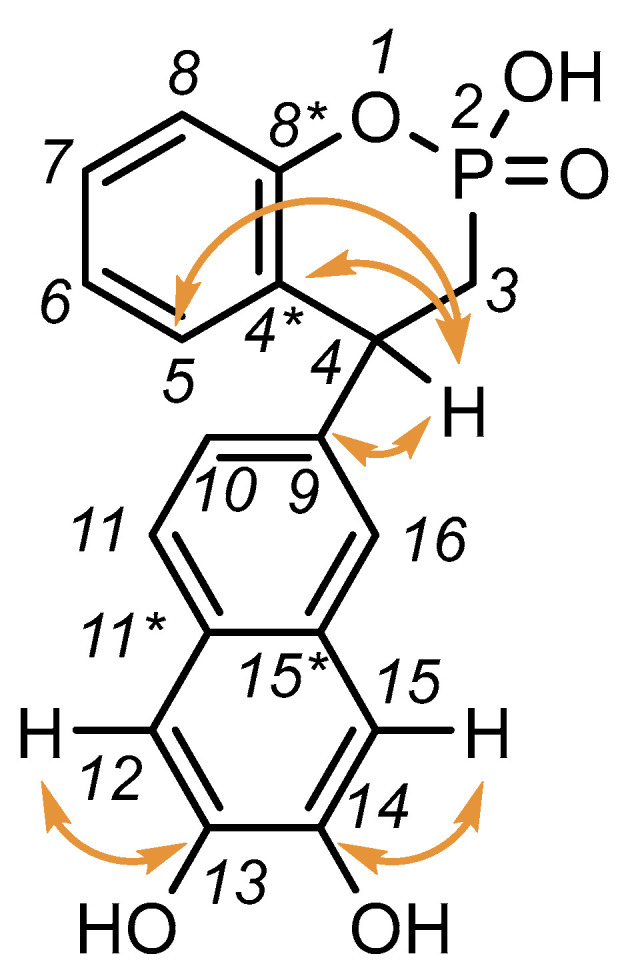
Structure determination of the compound **2j**. Key cross-peaks in the ^1^H-^13^C HMBC spectrum are indicated by arrows.

The compound 2-naphthol and naphthalene-2,3-diol also reacted smoothly to provide the compounds **2i** and **2j**. Multiple 2D NMR experiments have been carried out for the compound **2j** to elucidate the substitution site at the naphthalene core (Figure 1). The NMR signals were assigned on the basis of ^1^H, ^13^C, ^1^H-^1^H COSY, ^1^H-^13^C HMBC and ^1^H-^13^C HSQC experiments (see the Supporting Materials). In the ^1^H-^13^C HMBC spectrum, a cross-peak is observed between the proton of a methyne group H-C^4^ and C^5^ and C^8^ carbon atoms, as well as a cross-peak between the same proton and the C^9^ carbon atom. Additionally, two cross-peak are present between the protons *ortho* to the hydroxyl groups (H-C^12^ and H-C^15^) and the C^13^ and C^14^ carbon atoms, respectively.

**Scheme 3:**
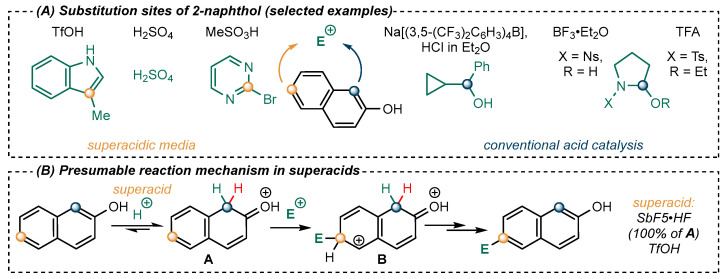
Selected examples highlighting the preferred substitution site of 2-naphthol in acid- [43,44,45] and superacid-catalyzed reactions [46,47,48] (**A**) and the tentative reaction mechanism in superacidic medium (**B**).

All of the above unequivocally indicates the substitution to Position 6 of the naphthalene ring. This is a somewhat unexpected result, since the position *ortho* to the hydroxy groups is generally supposed to be the most reactive. Intrigued by this discrepancy, we searched the literature for reactions of 2-naphthol with carbon electrophiles, paying particular attention to the reaction conditions. The analysis of the collected data revealed that the conventional acidic catalysts promote the substitution at Position 1 of 2-naphthol (for typical examples, see [43,44,45]). At the same time, when the reaction with an electrophile is carried out in the presence of superacids or in a sulfuric acid solution, the electrophilic attack preferably occurs at the sixth position (Scheme 3A) [46,47,48].

The compound 2-naphthol is known to undergo exhaustive C^1^-protonation in superacidic media, as was reported by Olah [49] and Hartmann and Yu [50]. We speculate that this may be the reason for different substitution patterns in the case of acidic and superacidic catalysts. Presumably, the protonation of the C^1^ atom in the presence of a superacid forces the electrophilic attack to the C^6^ atom of a fused benzene ring (Scheme 3B). The initial protonation deactivates the hydroxylated aromatic ring against further reactions with electrophiles. This opens up the possibility of directing the substitution at the desired site by regulating the acidity of a reaction media. As far as we know, such a possibility has never been stated for the naphthols before. A separate study is needed to further test this hypothesis; however, this falls out of the scope of the present paper. Nonetheless, this finding also indirectly indicates the superacidic nature of the reaction media in our case.

### 2.2. Exploration of the Reaction Mechanism by Quantum Chemistry Calculations

As was noted above, a superelectrophilic activation is often associated with the formation of doubly protonated dicationic species. Thus, a comparison of the reaction barriers for the mono- and dicationic species would provide additional insight into the reaction mechanism and further clarify the possibility of the superelectrophilic activation of phosphacoumarin under the reaction conditions. With this in mind, we carried out quantum chemistry calculations. The calculations were performed at the ωB97X-V/def2-TZVPD//PBE/def2-TZVPD theory level with Orca 5.0.3 software (see the Supplementary Materials for additional details on method choice) [51]. The solvent effect was accounted for using the C-PCM [52] model with TFA as a solvent. We have chosen the interaction of the unsubstituted phosphacoumarin **PHOS** with toluene as the model reaction. Note that due to the complex equilibria between monomeric, dimeric, trimeric, etc., species in TFA [53], it is hard to estimate the most feasible counter-anions. Additionally, the compound **1** is also an acid, and thus may take part in these equilibria, forming complexes with TFA. This complicates the task even more, especially given that its concentration in TFA should also be accounted for. Thus, the effect of the counter-ion was neglected for these calculations.

**Scheme 4:**
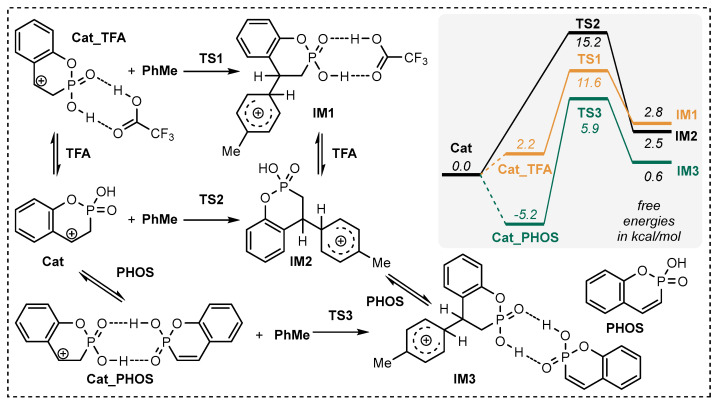
Possible reaction pathways and free energy profiles (kcal/mol) for the interaction of the monocationic phosphacoumarin electrophile with toluene as obtained from quantum chemistry calculations (ωB97X-V/def2-TZVPD//PBE/def2-TZVPD, C-PCM(TFA), Orca 5.0.3).

First, we calculated the energies of the cation **Cat** and its complexes with TFA (**Cat_TFA**) and the phosphacoumarin (**Cat_PHOS**) (Scheme 4). The obtained data suggest that the formation of the TFA complex **Cat_TFA** is slightly endergonic (2.2 kcal/mol) and the complex **Cat_PHOS** is more energetically favorable (by 5.2 kcal/mol). Next, we calculated the transition state energies for the reaction of these species with toluene.

The energy of the transition state **TS2** for the reaction of the parent cation **Cat** with benzene was the highest among them all (15.2 kcal/mol). The formation of the hydrogen-bonded complexes **Cat_TFA** and **Cat_PHOS** lowers the energy of the transition states **TS1** and **TS3** by 3.6 kcal/mol and 9.3 kcal/mol, respectively. Complexation with phosphacoumarin lowers the relative energy of the H-bonded cationic intermediate **IM3** (0.6 kcal/mol) compared to the energy of the simpler intermediate **IM2** (2.5 kcal/mol). On the contrary, the TFA-bonded intermediate **IM1** is somewhat higher in energy (2.8 kcal/mol), albeit the difference is rather small.

With all of the above, the energetically most favorable reaction pathway involves the formation of the complex **Cat_PHOS** and its interaction with the toluene molecule through the transition state **TS3** to give the intermediate **IM3**.

**Scheme 5:**
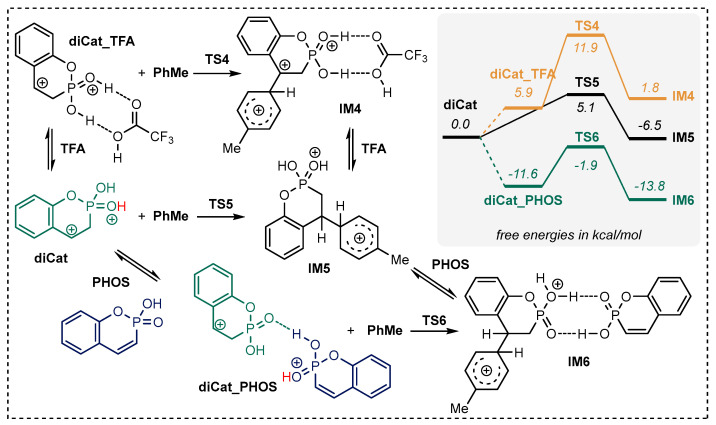
Possible reaction pathways and free energy profiles (kcal/mol) for the interaction of the dicationic phosphacoumarin electrophile with toluene as obtained from quantum chemistry calculations (ωB97X–V/def2-TZVPD//PBE/def2–TZVPD, C–PCM(TFA), Orca 5.0.3).

Similarly, we have calculated the relative energies of the dications **diCat**, **diCat_TFA** and **diCat_PHOS**, as well as the energies of their respective transition states **TS5**, **TS4**, **TS6** and intermediates **IM4**, **IM5** and **IM6** (Scheme 5). The complexation of the dication **diCat** with TFA is accompanied by a considerable energy penalty (5.9 kcal/mol). The complexation with TFA destabilizes both the transition state **TS4** and the intermediate **IM4** compared to their non-complexed counterparts (energy difference is 6.8 kcal/mol and 8.3 kcal/mol, respectively).

**Scheme 6:**
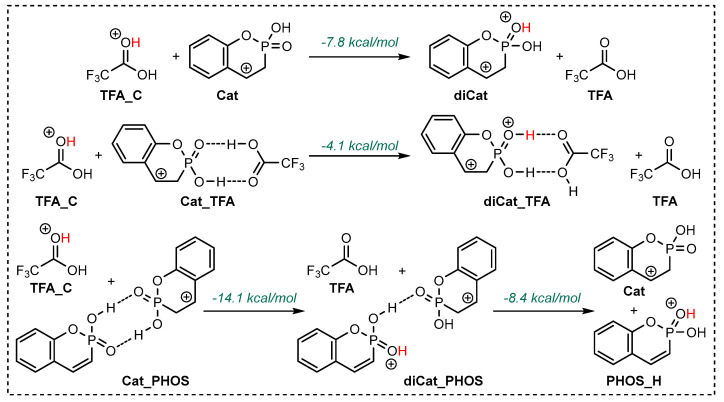
Free energies for the protonation of monocationic organophosphorus electrophiles as obtained from quantum chemistry calculations (ωB97X–V/def2–TZVPD//PBE/def2–TZVPD, C–PCM(TFA), Orca 5.0.3).

In contrast, the formation of the complex with phosphacoumarin is strongly exergonic (−11.6 kcal/mol). The analysis of the geometry of the optimized complex **diCat_PHOS** suggests the migration of the proton from the phosphoryl group of **diCat** to the phosphoryl group of **PHOS** upon complexation (see the Supplementary Materials, Figure S4 for key bond lengths). This leads to the localization of two positive charges on different molecules, leading to the stabilization of the complex. The same factor results in much lower energies of the transition state **TS6** (−1.9 kcal/mol relative to reactants) and the intermediate **IM6** (−13.8 kcal/mol) compared to the non-complexed **TS5** (5.1 kcal/mol) and **IM5** (−6.5 kcal/mol). Consequently, one should assume the formation of the intermediate **IM6** via the transition state **TS6** to be the most favorable pathway of the reaction. However, there are some points to note here. First of all, these species can be considered a complex of two hydrogen-bonded cations **Cat** and **PHOS_H** rather than dications. Moreover, the complex **diCat_PHOS** is 8.4 kcal/mol higher in energy compared to the cations **Cat** and **PHOS_H** (see Scheme 6). Thus, it is more likely to dissociate to the cations **Cat** and **PHOS_H** (downhill by 8.4 kcal/mol) rather than react with the toluene molecule (uphill by 9.7 kcal/mol). Taken altogether, these considerations make the formation of the intermediate **IM5** from non-complexed dication **diCat** more feasible.

We have also tried to estimate the relative energies of mono- and dicationic species. Note that multiple positively charged ions are likely to be present in TFA solution, including dimeric, trimeric and polymeric species [53]. Therefore, the accurate prediction of the nature of the protonated species has to account for all of them. This makes such a calculation a non-trivial and computationally demanding task. Herein, we were interested primarily in the relative energies of the cations rather than the elucidation of all possible molecular ensembles. Thus, we utilized the simplest protonated TFA molecule (**TFA_Cat**) as a proton source. Although not being completely realistic, this can serve as a suitable approximation for our purposes. The protonation of the monocation **Cat**, as well as the cationic complexes **Cat_TFA** and **Cat_PHOS**, appeared to be exergonic by 7.8, 4.1 and 14.1 kcal/mol, respectively (Scheme 6); that is, the proton transfer from the cation **TFA_Cat** to these cations is favorable. As was mentioned above, the complex **diCat_PHOS** is likely to undergo further dissociation to the cations **Cat** and **PHOS_H**, and thus its presence in the reaction mixture is at least questionable. This suggests that the doubly protonated **diCat** is the most probable reactive species.

In conclusion, the quantum chemistry calculations suggest that the reaction of phosphacoumarins with aromatics is most likely to proceed via the monocationic complex of *C*-protonated phosphacoumarin with the second molecule of phosphacoumarin (**Cat_PHOS**) or TFA (**Cat_TFA**) or via the doubly protonated phosphacoumarin **diCat**. The energies of the respective transition states **TS1, TS3** and **TS5** for the reaction with benzene are 11.6, 5.9 and 5.1 kcal/mol relative to the reactants, which is lower than the energy of the transition state in case of the monocation **Cat** (15.2 kcal/mol). Additionally, the proton transfer from the protonated form of TFA to the *C*-protonated phosphacoumarin **Cat** to create the dicationic intermediate **diCat** is accompanied by considerable increase in energy (7.8 kcal/mol). Overall, these data support our hypothesis on the superelectrophilic activation of the phosphacoumarin and the important role of the hydrogen-bonded complexes.

## 3. Materials and Methods

### 3.1. General Methods

The ^1^H and ^13^C NMR spectra were recorded on a Bruker Avance 600 spectrometer (Bruker, Billerica, MA, USA) (operating frequency 600 MHz and 150 MHz, respectively) with respect to the residual proton signals of deuterated solvents (DMSO-*d*_6_, CDCl_3_). ^31^P spectra were recorded on a Bruker MSL 400 (162 MHz) spectrometer (Bruker, Billerica, MA, USA) using 85% H_3_PO_4_ as an external reference. Multiplicity was indicated as follows: s (singlet), d (doublet), t (triplet), q (quartet), m (multiplet), dd (doublet of doublet), td (triplet of doublet), bs (broad singlet). Coupling constants were reported in Hertz (Hz). The IR spectra were recorded on a Vector 22 Fourier spectrometer (Bruker, Billerica, MA, USA) in the range of 400–4000 cm^−1^ from KBr pellets. The melting points were determined in glass capillaries on a Stuart SMP 10 instrument (Stuart, Bibby Scientific Ltd., Stone, UK). The elemental analysis was carried out on a CHNS analyzer Vario Macro cube (Elementar Analysensysteme GmbH, Langenselbold, Germany). The samples were weighed on a Sartorius Cubis II (Germany) microbalance in tin capsules. VarioMacro Software V4.0.11 (Abacus Analytical Systems GmbH, Berlin, Germany) was used to perform quantitative measurements and evaluate the data received. ESI-TOF-MS spectra were recorded on a Bruker AmazonX instrument (Bruker, Billerica, MA, USA). The halogen content was determined by the Schöniger method. The solvents were purified according to standard procedures. Commercially available reagents were used without additional purification. The compound 2-Hydroxybenzo[*e*][1,2]oxaphosphinine 2-oxide **1** was obtained by the previously reported procedure [42].

### 3.2. X-Ray Analysis

X-ray diffraction (XRD) spectra of the single crystals **2b**, **2c** and **3** were obtained on a Bruker D8 QUEST automated three-circle diffractometer with a PHOTON III area detector and an IμS DIAMOND microfocus X-ray tube: λ(Mo *K*α) = 0.71073 Å, ω/ϕ scanning mode with a step of 0.5°. Data collection and indexing, determination and refinement of the unit cell parameters were carried out using the *APEX3* software package. Numerical absorption correction based on the crystal shape, additional spherical absorption correction and systematic error correction were performed using the *SADABS*-2016/2 software [54]. Using *OLEX2*, 59 structures were solved by direct methods using the *SHELXT*-2018/3 program [55] and refined by full-matrix least-squares on *F*^2^ using the *SHELXL*-2018/3 program. Nonhydrogen atoms were refined anisotropically. The positions of the H(O) hydrogen atom were determined from difference electron density maps and refined isotropically. The positions of the hydrogen atoms of methyl groups were inserted using the rotation of the group with idealized bond angles; the remaining hydrogen atoms were refined using a riding model. Most calculations were performed using the *WinGX*-2021.3 software package [56]. Crystallographic data for the investigated structures are in the Supplementary Materials and deposited in the Cambridge Crystallographic Data Centre as Supplementary Publication Nos. CCDC 2299351, 2321868 and 2299352.

### 3.3. Computational Details and Methods

All calculations were performed with the ORCA 5.0.3 package [51]. Geometry optimizations were performed at the PBE/def2-TZVPD theory level with the atom-pairwise dispersion correction with the Becke–Johnson damping scheme [57,58] (D3BJ) using a resolution of identity approximation for Coulomb integrals (RI-J) with the def2/J auxiliary basis set [59]. All optimizations were followed by frequency calculations at the ωB97X-V/def2-TZVPD level of theory with the D3BJ dispersion correction and RI-J approximation (def2/J auxiliary basis set) and numerical chain-of-sphere integration for the HF exchange integrals (COSX). The structure was accepted if and only if no imaginary frequencies were present (for ground states) or if exactly one imaginary frequency was present (for transition states). The IRC calculations were performed to verify that the obtained transition state really connects two minima. The implicit solvation model (PCM, TFA as a solvent) was used to account for the solvent effects. The following values were used for the refraction index and dielectric constant of TFA: *ε* = 8.55, *R_D_* = 1.285.

### 3.4. Synthesis of Phosphacoumarin **1**

The compound 2-ethoxyvinylphosphonic acid dichloroanhydride (5.3 mmol, 1 equiv) in toluene (5 mL) was added dropwise to a mixture of 2,3,5-trimethylphenol (10.6 mmol, 2 equiv) and trifluoroacetic acid (5.3 mmol, 1 equiv) in boiling toluene (30 mL). The reaction mixture was refluxed for 3 h. The oil that precipitated from the reaction mixture was heated in isopropyl alcohol until a white precipitate formed. The precipitate was filtered and dried under vacuum to a constant weight to give the target compound **1** as a white solid; yield 1 g 85%; m.p. 206–208 °C. IR (KBr, cm^−1^) 1225, 1590, 1617, 2924. ^1^H NMR (DMSO-*d_6_*, 400 MHz): δ 7.54 (dd, 1H, J(H,P) = 42.2, J(H,H) = 12.8 Hz), 6.85 (s, 1H), 6.24 (dd, 1H, J(H,P) = 20.3, J(H,H) = 12.8 Hz), 2.34 (s, 3H), 2.24 (s, 3H), 2.14 (s, 3H). ^13^C{^1^H} NMR (DMSO-*d_6_*, 151 MHz): δ 150.3 (d, J(C,P) = 9.0 Hz), 139.8, 139.3, 134.0, 126.6, 123.4 (d, J(C,P) = 5.9 Hz), 117.6 (d, J(C,P) = 17.9 Hz), 115.7, 114.5, 20.2, 19.0, 11.7. ^31^P{^1^H} NMR (DMSO-*d_6_*, 162 MHz): δ 5.3. Anal. Calcd for C_11_H_13_O_3_P: C, 58.93; H, 5.84; P, 13.82. Found: C, 58.80; H, 5.83; P, 13.76. MS (ESI) *m*/*z* calcd for 224.2, found 247.2 [M + Na]^+^.

### 3.5. General Procedure for Synthesis of Compounds **2**

The compound 2-hydroxy-5,7,8-trimethylbenzo[e][1,2]oxaphosphinine 2-oxide **1** (1.3 mmol, 1 equiv) was added to a solution of appropriate arene (1.3 mmol, 1 equiv) in trifluoroacetic acid (8 mL). The reaction mixture was heated at 70 °C. The reaction completion was monitored by ^31^P{^1^H} NMR.

The compound *2-hydroxy-5,7,8-trimethyl-4-(p-tolyl)-3,4-dihydrobenzo[e][1,2]oxaphosphinine 2-oxide* (**2a**) was synthesized according to the general procedure. The reaction mixture was stirred for 10 h. The formed precipitate was filtered off, washed with 20 mL of diethyl ether and dried under vacuum to a constant weight. White solid; yield 0.2 g, 50%; m.p. 290−292 °C. IR (KBr, cm^−1^): 1215, 1616, 2866, 2919, 2943, 3429. ^1^H NMR (DMSO-*d_6_*, 600 MHz): δ 7.04 (d, 2H, J = 8.0 Hz), 6.97 (d, 2H, J = 8.1 Hz), 6.78 (s, 1H), 4.62 (dd, 0.5H, J(H,P) = 36.0, J(H,H) = 3.5 Hz), 4.56 (dd, 0.5H, J(H,P) = 36.0, J(H,H) = 3.5 Hz), 2.50–2.32 (m, 2H), 2.24 (s, 3H), 2.21 (s, 3H), 2.11 (s, 3H), 2.02 (s, 3H). ^13^C{^1^H} NMR (DMSO-*d_6_*, 101 MHz): δ 151.0 (d, J(C,P) = 7.4 Hz), 140.2 (d, J(C,P) = 3.1 Hz), 137.2, 136.2, 134.4, 129.8, 128.3, 127.6, 125.2 (d, J(C,P) = 11.1 Hz), 124.8 (d, J(C,P) = 4.9 Hz), 39.3 (d, J(C,P) = 7.3 Hz), 30.3 (d, J(C,P) = 128.5 Hz), 21.5, 20.4, 19.9, 12.6. ^31^P{^1^H} NMR (DMSO-*d_6_*, 243 MHz): δ 21.8. Anal. Calcd for C_18_H_21_O_3_P: C, 68.22; H, 6.69; P, 9.79. Found: C, 68.22; H, 6.59; P, 9.71. MS (ESI) *m*/*z* calcd for 316.1, found 317.1 [M + H]^+^; 339.1 [M + Na]^+^.

The compound *4-(3,4-dimethylphenyl)-2-hydroxy-5,7,8-trimethyl-3,4-dihydrobenzo[e][1,2]oxaphosphinine 2-oxide* (**2b**) was synthesized according to the general procedure. The reaction mixture was stirred for 10 h. The formed precipitate was filtered off, washed with 20 mL of diethyl ether and dried under vacuum to a constant weight. White solid; yield 0.15 g, 36%; m.p. 258−260 °C. IR (KBr, cm^−1^): 1212, 1614, 2920, 2942, 2969, 3426. ^1^H NMR (DMSO-*d_6_*, 600 MHz): δ 6.97 (d, 1H, J = 7.8 Hz), 6.91 (s, 1H), 6.77 (s, 1H), 6.74 (dd, 1H, J = 7.7 Hz), 4.58 (dd, 0.5H, J(H,P) = 35.6, J(H,H) = 2.7 Hz), 4.53 (dd, 0.5H, J(H,P) = 35.6, J(H,H) = 2.7 Hz), 2.47–2.31 (m, 2H), 2.21 (s, 3H), 2.15 (s, 3H), 2.14 (s, 3H), 2.12 (s, 3H), 2.01 (s, 3H). ^13^C{^1^H} NMR (DMSO-*d_6_*, 101 MHz): δ 151.0 (d, J(C,P) = 7.4 Hz), 140.6 (d, J(C,P) = 3.0 Hz), 137.1, 136.7, 134.9, 134.5, 130.3, 129.5, 127.6, 125.7, 125.2 (d, J(C,P) = 11.1 Hz), 124.8 (d, J(C,P) = 4.8 Hz), 39.3 (d, J(C,P) = 7.2 Hz), 30.4 (d, J(C,P) = 128.4 Hz), 20.6, 20.4, 20.0, 19.9, 12.6. ^31^P{^1^H} NMR (DMSO-*d_6_*, 243 MHz): δ 21.9. Anal. Calcd for C_19_H_23_O_3_P: C, 69.08; H, 7.02; P, 9.38. Found: C, 68.98; H, 6.90; P, 9.29. MS (ESI) *m*/*z* calcd for 330.2, found 331.2 [M + H]^+^; 353.2 [M + Na]^+^.

The compound *4-(2,4-dimethylphenyl)-2-hydroxy-5,7,8-trimethyl-3,4-dihydrobenzo[e][1,2]oxaphosphinine 2-oxide* (**2c**) was synthesized according to the general procedure. The reaction mixture was stirred for 10 h. The solvent was eliminated under reduced pressure, the resulting oily residue was dissolved in 10 mL of diethyl ether. The formed precipitate was filtered off, washed with 20 mL of diethyl ether and dried under reduced pressure to a constant weight. White solid; yield 0.11 g, 25%; m.p. 289−291 °C. IR (KBr, cm^−1^): 1222, 1615, 2863, 2919, 2947, 3427. ^1^H NMR (DMSO-d_6_, 600 MHz): δ 6.98 (s, 1H), 6.77 (d, 1H, J = 7.6 Hz), 6.72 (s, 1H), 6.55 (d, 1H, J = 7.8 Hz), 4.68 (dd, 0.5H, J(H,P) = 29.9, J(H,H) = 3.7 Hz), 4.63 (dd, 0.5H, J(H,P) = 29.9, J(H,H) = 3.7 Hz), 2.44–2.38 (m, 1H), 2.37 (s, 3H), 2.21 (s, 3H), 2.20 (s, 3H), 2.15 (s, 3H), 2.11–2.05 (m, 1H), 1.82 (s, 3H). ^13^C{^1^H} NMR (DMSO-d_6_, 101 MHz): δ 151.6 (d, J(C,P) = 7.1 Hz), 138.9 (d, J(C,P) = 3.5 Hz), 137.0, 136.2, 135.6, 134.6, 131.9, 128.5, 127.6, 127.3, 124.5 (d, J(C,P) = 12.2 Hz), 124.5 (d, J(C,P) = 5.2 Hz), 36.9 (d, J(C,P) = 6.9 Hz), 28.6 (d, J(C,P) = 126.6 Hz), 21.4, 20.4, 19.8, 19.7, 12.7. ^31^P{^1^H} NMR (DMSO-d_6_, 243 MHz): δ 19.9. Anal. Calcd for C_19_H_23_O_3_P: C, 69.08; H, 7.02; P, 9.38. Found: C, 68.96; H, 6.93; P, 9.27. MS (ESI) *m*/*z* calcd for 330.2, found 331.2 [M + H]^+^; 353.2 [M + Na]^+^.

The compound *4-(5-chloro-2,4-dihydroxyphenyl)-2-hydroxy-5,7,8-trimethyl-3,4-dihydrobenzo[e][1,2]oxaphosphinine 2-oxide* (**2d**) was synthesized according to the general procedure. The reaction mixture was stirred for 7 h. The formed precipitate was filtered off, washed with 20 mL of diethyl ether and dried under vacuum to a constant weight. White solid; yield 0.29 g, 61%; m.p. 265−267 °C. IR (KBr, cm^−1^): 1200, 1620, 2864, 2921, 2973, 3428. ^1^H NMR (DMSO-*d_6_*, 400 MHz): δ 9.81 (s, 1H), 9.78 (s, 1H), 6.78 (s, 1H), 6.54 (s, 1H), 6.28 (s, 1H), 4.70 (dd, 0.5H, J(H,P) = 34.5, J(H,H) = 3.3 Hz), 4.61 (dd, 0.5H, J(H,P) = 34.5, J(H,H) = 3.3 Hz), 2.44–2.35 (m, 1H), 2.23 (s, 3H), 2.20–2.16 (m, 1H), 2.14 (s, 3H), 1.94 (s, 3H). ^13^C{^1^H} NMR (DMSO-*d_6_*, 101 MHz): δ 154.5, 152.4, 151.0 (d, J(C,P) = 7.4 Hz), 136.7, 133.8, 129.0, 127.1, 124.1 (d, J(C,P) = 5.2 Hz), 123.8 (d, J(C,P) = 11.3 Hz), 120.4, 109.4, 103.9, 33.7 (d, J(C,P) = 7.4 Hz), 27.4 (d, J(C,P) = 128.0 Hz), 19.9, 19.1, 12.2. ^31^P{^1^H} NMR (DMSO-*d_6_*, 162 MHz): δ 22.5. Anal. Calcd for C_17_H_18_ClO_5_P: C, 55.37; H, 4.92; Cl, 9.61; P, 8.40. Found: C, 55.25; H, 4.83; P, 8.31. MS (ESI) *m*/*z* calcd for 368.1, found 369.1 [M + H]^+^; 399.1 [M + Na]^+^.

The compound *4-(5-bromo-2,4-dihydroxyphenyl)-2-hydroxy-5,7,8-trimethyl-3,4-dihydrobenzo[e][1,2]oxaphosphinine 2-oxide* (**2e**) was synthesized according to the general procedure. The reaction mixture was stirred for 7 h. The formed precipitate was filtered off, washed with 20 mL of diethyl ether and dried under vacuum to a constant weight. White solid; yield 0.43 g, 81%; m.p. 268−270 °C. IR (KBr, cm^−1^): 1200, 1616, 2865, 2922, 2972, 3410. ^1^H NMR (DMSO-*d_6_*, 600 MHz): δ 9.85 (s, 1H), 9.84 (s, 1H), 6.77 (s, 1H), 6.54 (s, 1H), 6.43 (s, 1H), 4.68 (dd, 0.5H, J(H,P) = 35.4, J(H,H) = 2.9 Hz), 4.65 (dd, 0.5H, J(H,P) = 35.4, J(H,H) = 2.9 Hz), 2.41–2.36 (m, 1H), 2.23 (s, 3H), 2.21–2.16 (m, 1H), 2.14 (s, 3H), 1.94 (s, 3H). ^13^C{^1^H} NMR (DMSO-*d_6_*, 101 MHz): δ 155.7, 154.0, 151.5 (d, J(C,P) = 7.2 Hz), 137.2, 134.3, 132.3, 127.6, 124.5 (d, J(C,P) = 4.9 Hz), 124.3 (d, J(C,P) = 11.2 Hz), 121.6, 104.3, 98.7, 33.8 (d, J(C,P) = 7.1 Hz), 27.9 (d, J(C,P) = 127.9 Hz), 20.4, 19.6, 12.7. ^31^P{^1^H} NMR (DMSO-*d_6_*, 243 MHz): δ 21.5. Anal. Calcd for C_17_H_18_BrO_5_P: C, 49.42; H, 4.39; Br, 19.34; P, 7.50. Found: C, 49.36; H, 4.32; Br, 19.26; P, 7.43. MS (ESI) *m/z* calcd for 412.0, found 435.3 [M + Na]^+^.

The compound *4-(2,4-dihydroxy-5-methylphenyl)-2-hydroxy-5,7,8-trimethylbenzo[e][1,2]oxaphosphinine 2-oxide* (**2f**) was synthesized according to the general procedure. The reaction mixture was stirred for 7 h. The solvent was eliminated under reduced pressure, the resulting oily residue was dissolved in 10 mL of diethyl ether. The formed precipitate was filtered off, washed with 20 mL of diethyl ether and dried under reduced pressure to a constant weight. White solid; yield 0.16 g, 37%; m.p. 232−234 °C. IR (KBr, cm^−1^): 1214, 1619, 2925, 2972, 3341. ^1^H NMR (DMSO-*d_6_*, 400 MHz): δ 9.22 (s, 1H), 8.90 (s, 1H), 6.73 (s, 1H), 6.35 (s, 1H), 6.13 (s, 1H), 4.69 (dd, 0.5H, J(H,P) = 33.6, J(H,H) = 3.1 Hz), 4.60 (dd, 0.5H, J(H,P) = 33.6, J(H,H) = 3.1 Hz), 2.39–2.30 (m, 1H), 2.21 (s, 3H), 2.18–2.17 (m, 1H), 2.14 (s, 3H), 1.91 (s, 3H), 1.80 (s, 3H). ^13^C{^1^H} NMR (DMSO-*d_6_*, 101 MHz): 155.2, 153.8, 151.6 (d, J(C,P) = 7.3 Hz), 136.7, 134.4, 130.5, 127.5, 125.2 (d, J(C,P) = 11.3 Hz), 124.4 (d, J(C,P) = 5.2 Hz), 119.2 (d, J(C,P) = 5.2 Hz), 114.2, 103.0, 33.8 (d, J(C,P) = 7.3 Hz), 28.7 (d, J(C,P) = 127.4 Hz), 20.4, 19.7, 16.5, 12.7. ^31^P{^1^H} NMR (DMSO-*d_6_*, 162 MHz): δ 22.2. Anal. Calcd for C_18_H_19_O_5_P: C, 62.43; H, 5.53; P, 8.94. Found: C, 62.37; H, 5.49; P, 8.87.

The compound *4-(5-ethyl-2,4-dihydroxyphenyl)-2-hydroxy-5,7,8-trimethylbenzo[e][1,2]oxaphosphinine 2-oxide* (**2g**) was synthesized according to the general procedure. The reaction mixture was stirred for 7 h. The solvent was eliminated under reduced pressure, the resulting oily was dissolved in 10 mL of diethyl ether. The formed precipitate was filtered off, washed with 20 mL of diethyl ether and dried under reduced pressure to a constant weight. White solid; yield 0.21 g, 45%; m.p. 218−220 °C. IR (KBr, cm^−1^): 1219, 1617, 2873, 2923, 2967, 3299. ^1^H NMR (DMSO-*d_6_*, 400 MHz): δ 9.20 (s, 1H), 8.86 (s, 1H), 6.73 (s, 1H), 6.34 (s, 1H), 6.18 (s, 1H), 4.69 (dd, 0.5H, J(H,P) = 32.8, J(H,H) = 4.1 Hz), 4.61 (dd, 0.5H, J(H,P) = 32.8, J(H,H) = 4.1 Hz), 2.36–2.24 (m, 2H), 2.21 (s, 3H), 2.19–2.15 (m, 2H), 2.21 (s, 3H), 2.13 (s, 3H), 1.90 (s, 3H), 1.09 (t, 3H, J = 7.0 Hz). ^13^C{^1^H} NMR (DMSO-*d_6_*, 101 MHz): 154.8, 153.7, 151.6 (d, J(C,P) = 7.3 Hz), 136.6, 134.5, 129.3, 127.5, 125.1 (d, J(C,P) = 11.3 Hz), 124.3 (d, J(C,P) = 5.1 Hz), 120.7, 119.4 (d, J(C,P) = 3.3 Hz), 103.1, 33.9 (d, J(C,P) = 7.1 Hz), 28.7 (d, J(C,P) = 127.2 Hz), 23.4, 20.4, 19.7, 15.7, 12.7. ^31^P{^1^H} NMR (DMSO-*d_6_*, 162 MHz): δ 21.8. Anal. Calcd for C_19_H_21_O_5_P: C, 63.33; H, 5.87; P, 8.60. Found: C, 63.28; H, 5.80; P, 8.52.

The compound *4-(5-hexyl-2,4-dihydroxyphenyl)-2-hydroxy-5,7,8-trimethylbenzo[e][1,2]oxaphosphinine 2-oxide* (**2h**) was synthesized according to the general procedure. The reaction mixture was stirred for 7 h. The solvent was eliminated under reduced pressure, the resulting oily residue was dissolved in 10 mL of diethyl ether. The formed precipitate was filtered off, washed with 20 mL of diethyl ether and dried under reduced pressure to a constant weight. White solid; yield 0.3 g, 55%; m.p. 203−205 °C. IR (KBr, cm^−1^): 1208, 1432, 1619, 2857, 2927, 2953, 3372. ^1^H NMR (DMSO-*d_6_*, 400 MHz): δ 9.19 (s, 1H), 8.82 (s, 1H), 6.72 (s, 1H), 6.33 (s, 1H), 6.13 (s, 1H), 4.68 (dd, 0.5H, J(H,P) = 32.4, J(H,H) = 3.5 Hz), 4.60 (dd, 0.5H, J(H,P) = 32.4, J(H,H) = 3.5 Hz), 2.38–2.24 (m, 2H), 2.21 (s, 3H), 2.19–2.15 (m, 2H), 2.13 (s, 3H), 1.89 (s, 3H), 1.30–1.23 (m, 2H), 1.18–1.11 (m, 6H), 1.09 (t, 3H, J = 7.0 Hz). ^13^C{^1^H} NMR (DMSO-*d_6_*, 101 MHz): 154.9, 153.7, 151.6 (d, J(C,P) = 7.2 Hz), 136.6, 134.5, 130.0, 127.4, 125.0 (d, J(C,P) = 11.4 Hz), 124.3 (d, J(C,P) = 5.2 Hz), 119.2 (d, J(C,P) = 3.5 Hz), 119.0, 103.1, 34.0 (d, J(C,P) = 7.1 Hz), 32.1, 30.2, 29.8, 29.0, 28.7 (d, J(C,P) = 127.1 Hz), 23.0, 20.4, 19.7, 14.9, 12.7. ^31^P{^1^H} NMR (DMSO-*d_6_*, 162 MHz): δ 21.7. Anal. Calcd for C_23_H_29_O_5_P: C, 66.33; H, 7.02; P, 7.44. Found: C, 66.29; H, 6.96; P, 7.39.

The compound *2-hydroxy-4-(6-hydroxynaphthalen-2-yl)-5,7,8-trimethyl-3,4-dihydrobenzo[e][1,2]oxaphosphinine 2-oxide* (**2i**) was synthesized according to the general procedure. The reaction mixture was stirred for 5 h. The formed precipitate was filtered off, washed with 20 mL of diethyl ether and dried under vacuum to a constant weight. White solid; yield 0.3 g, 63%; m.p. ˃ 300 °C. IR (KBr, cm^−1^): 1227, 1565, 1604, 2864, 2920, 3651. ^1^H NMR (DMSO-*d_6_*, 400 MHz): δ 9.60 (s, 1H), 7.58 (d, 1H, J = 8.8 Hz), 7.57 (d, 1H, J = 8.6 Hz), 7.34 (d, 1H, J = 1.8 Hz), 7.21 (dd, 1H, J = 8.6 Hz), 7.05 (d, 1H, J = 2.4 Hz), 7.01 (dd, 1H, J = 8.8 Hz), 6.80 (s, 1H), 4.78 (dd, 0.5H, J(H,P) = 35.3, J(H,H) = 2.9 Hz), 4.70 (dd, 0.5H, J(H,P) = 35.3, J(H,H) = 2.9 Hz), 2.56 (ddd, 1H, J(H,P) = 16.0, J(H,H) = 7.3 Hz), 2.41 (ddd, 1H, J(H,P) = 16.0, J(H,H) = 7.3 Hz), 2.24 (s, 3H), 2.14 (s, 3H), 2.04 (s, 3H). ^13^C{^1^H} NMR (DMSO-*d_6_*, 126 MHz): δ 155.0, 150.0 (d, J(C,P) = 7.4 Hz), 136.3, 136.2, 133.6, 133.3, 129.1, 127.4, 126.7, 126.3, 126.0, 125.3, 124.0 (d, J(C,P) = 11.0 Hz), 123.9 (d, J(C,P) = 4.6 Hz), 118.5, 108.4, 38.6 (d, J(C,P) = 7.2 Hz), 29.3 (d, J(C,P) = 127.9 Hz), 19.4, 18.9, 11.7. ^31^P{^1^H} NMR (DMSO-*d_6_*, 162 MHz): δ 21.6. Anal. Calcd for C_21_H_21_O_4_P: C, 68.47; H, 5.75; P, 8.41. Found: C, 68.39; H, 5.66; P, 8.32. MS (ESI) *m*/*z* calcd for 368.1, found 369.1 [M + H]^+^; 407.1 [M + Na]^+^.

The compound *4-(6,7-dihydroxynaphthalen-2-yl)-2-hydroxy-5,7,8-trimethyl-3,4-dihydrobenzo[e][1,2]oxaphosphinine 2-oxide* (**2j**) was synthesized according to the general procedure. The reaction mixture was stirred for 5 h. The formed precipitate was filtered off, washed with 20 mL of diethyl ether and dried under vacuum to a constant weight. White solid; yield 0.27 g, 55%; m.p. ˃ 300 °C. IR (KBr, cm^−1^): 1226, 1562, 1612, 2918, 2977, 3517. ^1^H NMR (DMSO-*d_6_*, 400 MHz): δ 9.41 (s, 2H), 7.44 (d, 1H, J = 8.5 Hz), 7.18 (d, 1H, J = 1.7 Hz), 7.02 (s, 1H), 6.98 (dd, 1H, J = 8.5 Hz), 6.91 (s, 1H), 6.79 (s, 1H), 4.73 (dd, 0.5H, J(H,P) = 35.1, J(H,H) = 3.0 Hz), 4.66 (dd, 0.5H, J(H,P) = 35.1, J(H,H) = 3.0 Hz), 2.54 (ddd, 1H, J(H,P) = 16.0, J(H,H) = 7.3 Hz), 2.34 (ddd, 1H, J(H,P) = 16.0, J(H,H) = 7.3 Hz), 2.23 (s, 3H), 2.14 (s, 3H), 2.03 (s, 3H). ^13^C{^1^H} NMR (DMSO-*d_6_*, 126 MHz): δ 150.1 (d, J(C,P) = 7.3 Hz), 146.9, 146.5, 136.6, 136.1, 133.6, 128.5, 127.4, 126.6, 125.6, 124.4 (d, J(C,P) = 11.8 Hz), 123.8 (d, J(C,P) = 4.6 Hz), 123.4, 123.0, 109.4, 109.2, 38.7 (d, J(C,P) = 7.2 Hz), 29.3 (d, J(C,P) = 128.1 Hz), 19.4, 19.0, 11.7. ^31^P{^1^H} NMR (DMSO-*d_6_*, 162 MHz): δ 21.9. Anal. Calcd for C_21_H_21_O_5_P: C, 65.62; H, 5.51; P, 8.06. Found: C, 65.53; H, 5.43; P, 7.98. MS (ESI) *m*/*z* calcd for 384.1, found 385.1 [M + H]^+^.

The compound *4-Hydroxy-3-(2-hydroxy-5,7,8-trimethyl-2-oxido-3,4-dihydrobenzo[e][1,2]oxaphosphinin-4-yl)-2H-chromen-2-one* (**2k**) was synthesized according to the general procedure. The reaction mixture was stirred for 6 h. The solvent was eliminated under reduced pressure, and the resulting oily was dissolved in 10 mL of diethyl ether. The precipitate was filtered off, the solvent was evaporated under vacuum and the oily residue was heated in isopropanol. The precipitate that formed was filtered off and dried in a vacuum to a constant weight. White solid; yield 0.12 g, 25%; m.p. ˃ 300 °C. IR (KBr, cm^−1^): 1204, 1653, 1717, 2924, 2963, 3420. ^1^H NMR (DMSO-*d_6_*, 400 MHz): δ 8.00 (dd, 1H, J = 8.3 Hz), 7.68 (m, 2H), 7.46 (s, 2H), 4.32 (dt, 1H, J(H,P) = 30.0, J(H,H) = 5.2 Hz), 2.32 (s, 3H), 2.30 (s, 3H), 2.26 (m, 1H), 2.23 (s, 3H), 1.93 (m, 1H). ^13^C{^1^H} NMR (DMSO-*d_6_*, 100.6 MHz): δ 162.0, 157.3, 153.1, 149.1, 136.8, 133.8, 133.1, 129.0, 125.3, 123.5, 122.1, 120.2 (d, J(C,P) = 2.8 Hz), 117.4, 115.5, 104.2 (d, J(C,P) = 4.1 Hz), 27.0 (d, J(C,P) = 108.2 Hz), 20.2, 19.0, 12.6. ^31^P{^1^H} NMR (DMSO-*d_6_*, 162 MHz): δ 22.2. Anal. Calcd for C_20_H_19_O_6_P: C, 62.18; H, 4.96; P, 8.02. Found: C, 62.09; H, 4.89; P, 7.93. MS (ESI) *m*/*z* calcd for 386.0, found 387.0 [M + H]^+^, 409.0 [M + Na]^+^.

The compound *2,4-dihydroxy-5-(2-hydroxy-5,7,8-trimethyl-2-oxido-3,4-dihydrobenzo[e][1,2]oxaphosphinin-4-yl)-3-methylbenzaldehyde* (**2l**) was synthesized according to the general procedure. The reaction mixture was stirred for 10 h. The formed precipitate was filtered off, washed with 20 mL of diethyl ether and dried under vacuum to a constant weight. White solid; yield 0.16 g, 34%; m.p. 233−235 °C. IR (KBr, cm^−1^): 1209, 1622, 1638, 2865, 2922, 3302. ^1^H NMR (DMSO-*d_6_*, 400 MHz): δ 10.12 (s, 1H), 9.51 (s, 1H), 6.79 (s, 1H), 6.69 (s, 1H), 4.86 (dd, 0.5H, J(H,P) = 33.8, J(H,H) = 3.4 Hz), 4.78 (dd, 0.5H, J(H,P) = 33.8, J(H,H) = 3.4 Hz), 2.41–2.26 (m, 2H), 2.23 (s, 3H), 2.16 (s, 3H), 2.10 (s, 3H), 1.94 (s, 3H). ^13^C{^1^H} NMR (DMSO-*d_6_*, 101 MHz): δ 196.2, 161.3, 160.9, 151.8 (d, J(C,P) = 7.3 Hz), 137.3, 134.4, 131.9, 127.7, 124.7 (d, J(C,P) = 5.3 Hz), 123.9 (d, J(C,P) = 11.1 Hz), 122.7 (d, J(C,P) = 3.0 Hz), 114.7, 111.6, 34.3 (d, J(C,P) = 7.3 Hz), 28.0 (d, J(C,P) = 127.9 Hz), 20.5, 19.7, 12.8, 9.2. ^31^P{^1^H} NMR (DMSO-*d_6_*, 162 MHz): δ 21.3. Anal. Calcd for C_19_H_21_O_6_P: C, 60.64; H, 5.62; P, 8.23. Found: C, 60.55; H, 5.54; P, 8.15. MS (ESI) *m*/*z* calcd for 376.1, found 399.1 [M + Na]^+^. 

The compound *4-(3,4-dihydroxyphenyl)-2-hydroxy-5,7,8-trimethylbenzo[e][1,2]oxaphosphinine 2-oxide* (**2m**) was synthesized according to the general procedure. The reaction mixture was stirred for 5 h. The solvent was eliminated under reduced pressure, the resulting oily was dissolved in 10 mL of diethyl ether. The formed precipitate was filtered off, recrystallized from isopropyl alcohol and dried under vacuum to a constant weight. White solid; yield 0.27 g, 60%; m.p. ˃ 300 °C. IR (KBr, cm^−1^): 1209, 1618, 2868, 2922, 2969, 33352. ^1^H NMR (DMSO-*d_6_*, 600 MHz): δ 8.60 (s, 2H), 6.75 (s, 1H), 6.57 (d, 1H, J = 8.1 Hz), 6.42 (s, 1H), 6.37 (d, 1H, J = 9.1 Hz), 4.43 (dd, 1H, J(H,P) = 34.9, J(H,H) = 5.2 Hz), 2.40–2.22 (m, 2H), 2.19 (s, 3H), 2.09 (s, 3H), 2.01 (s, 3H). ^13^C{^1^H} NMR (DMSO-*d_6_*, 101 MHz): δ 151.0 (d, J(C,P) = 7.6 Hz), 145.8, 144.6, 136.9, 134.5, 134.2 (d, J(C,P) = 3.1 Hz), 127.6, 125.7 (d, J(C,P) = 11.2 Hz), 124.7 (d, J(C,P) = 4.8 Hz), 119.2, 116.3, 115.8, 39.0 (d, J(C,P) = 7.1 Hz), 30.6 (d, J(C,P) = 128.0 Hz), 20.4, 19.9, 12.6. ^31^P{^1^H} NMR (DMSO-*d_6_*, 162 MHz): δ 22.2. Anal. Calcd for C_17_H_17_O_5_P: C, 61.45; H, 5.16; P, 9.32. Found: C, 61.37; H, 5.07; P, 9.25.

The compound *4-ethyl-1-hydroxy-8,9,11-trimethyl-12H-6,12-methanodibenzo[d,g][1,3,2]dioxaphosphocine-2-carbaldehyde 6-oxide* (**3**) was synthesized according to the general procedure. The reaction mixture was stirred for 10 h. The formed precipitate was filtered off, washed with 20 mL of diethyl ether and dried under vacuum to a constant weight. White solid; yield 0.12 g, 25%; m.p. 202−203 °C. IR (KBr, cm^−1^): 1234, 1632, 2871, 2970, 3022. ^1^H NMR (DMSO-*d_6_*, 600 MHz): δ 11.80 (s, 1H), 9.86 (s, 1H), 7.60 (s, 1H), 6.78 (s, 1H), 4.85 (dd, 0.5H, J(H,P) = 33.8, J(H,H) = 3.0 Hz), 5.19 (dt, 1H, J(H,P) = 35.8, J(H,H) = 4.1 Hz), 2.76–2.71 (m, 1H), 2.61–2.57 (m, 1H),2.52 (s, 3H), 2.50 (dt, 2H, J(H,H) = 3.6 Hz), 2.13 (s, 3H), 2.05 (s, 3H), 1.16 (t, 3H, J(H,H) = 7.5 Hz). ^13^C{^1^H} NMR (DMSO-*d_6_*, 101 MHz): δ 191.2, 158.5, 156.6 (d, J(C,P) = 8.2 Hz), 150.9 (d, J(C,P) = 8.2 Hz), 137.8, 134.5, 133.9, 124.8, 126.2 (d, J(C,P) = 6.9 Hz), 123.8 (d, J(C,P) = 3.0 Hz), 117.8, 116.0 (d, J(C,P) = 12.9 Hz), 31.6 (d, J(C,P) = 10.5 Hz), 22.8, 20.6 (d, J(C,P) = 112.3 Hz), 20.6, 20.2, 14.8, 12.5. ^31^P{^1^H} NMR (DMSO-*d_6_*, 162 MHz): δ 15.2. Anal. Calcd for C_20_H_21_O_5_P: C, 64.51; H, 5.68; P, 8.32. Found: C, 64.43; H, 5.60; P, 8.26. MS (ESI) *m*/*z* calcd for 372.1, found 373.1 [M + H]^+^, 395.3 [M + Na]^+^.

## 4. Conclusions

In summary, we have developed a convenient and efficient method for the synthesis of 4-aryl-substituted phosphaflavanoids through the TFA-assisted arylation of phosphacoumarins. The formation of dicationic species from phosphacoumarins or/and their hydrogen-bonded complexes plays a pivotal role in this process. Not only does this double activation enable the reaction with even weak nucleophiles, but it also demonstrates the successful application of the “Bronsted acid assisted Bronsted acid catalysis” concept in the superelectrophilic activation of organophosphorus compounds. The use of inexpensive reagents and mild reaction conditions are also the salient features of this protocol.

## Data Availability

The data underlying this study are available in the published article and its Supporting Materials.

## Supplementary Materials

The following supporting information can be downloaded at: https://www.mdpi.com/article/10.3390/ijms25126327/s1.
