# Supplementary material for: Superelectrophilic Activation of Phosphacoumarins towards Weak Nucleophiles via Brønsted Acid Assisted Brønsted Acid Catalysis
Source: Int J Mol Sci. 2024 Jun 7;25(12):6327. doi: 10.3390/ijms25126327 (PMC11203683; doi:10.3390/ijms25126327)
Supplement: Supplementary file 1 [file ijms-25-06327-s001.zip › ijms-2995446-supplementary.pdf]

Supporting Information

Superelectrophilic activation of phosphacoumarins towards weak nucleophiles via Brønsted acid assisted Brønsted acid catalysis

A.V. Zalaltdinova,[a] Y.M. Sadykova,[a] A.S. Gazizov,[a]* A.K. Smailov,[b] V.V. Syakaev,[a] D.P. Gerasimova,[a] E.A. Chugunova,[a] N.I. Akylbekov,[c,d] R.U. Zhapparbergenov,[d,f] N.O. Appazov,[c,d]* A.R. Burilov,[a] M.A. Pudovik,[a] I.V. Alabugin,[e] O.G. Sinyashin[a]

[a] *Arbuzov Institute of Organic and Physical Chemistry, FRC Kazan Scientific Center, Russian Academy of Science, Arbuzova str., 8, Kazan, 420088, Russian Federation*

[b] *Kazan National Research Technological University, Karl Marx str., 68, Kazan, 420015 Russia Federation*

[d] *Laboratory of Engineering Profile "Physical and Chemical Methods of Analysis", Korkyt Ata Kyzylorda University, Aitekebie str., 29A, Kyzylorda 120014, Kazakhstan*

[e] *CNEC "LLP, Dariger Ali Str. without a number, Kyzylorda 120001, Kazakhstan*

[f] *"DPS Kyzylorda" LLP, Amangeldy Imanov str., 112A, Kyzylorda 120008, Kazakhstan*

[c] *Department of Chemistry and Biochemistry, Florida State University, Tallahassee, Fl 32306, United States*

**Corresponding authors, email: agazizov@iopc.ru (A.S.), nurasar.82@mail.ru (N.A.)*

Table of Contents

X-ray data for the compounds 2b,c and 3 2

Quantum chemistry calculations 3

Copies of NMR spectra 5

Coordinates of stationary points 31

X-ray data for the compounds 2b,c and 3

Table S1. Crystallographic data and X-ray structural experiment parameters for the single crystals **2b**, **2c** and **3**.

Compound	**2b**	**2c**	**3**
Empirical formula	$C_{19}H_{23}O_3P$, CF_3COOH	$C_{19}H_{23}O_3P$	$C_{20}H_{21}O_5P$
Formula weight	444.37	330.34	372.34
Radiation, wavelength	Mo *K*α, 0.71073 Å	Mo *K*α, 0.71073 Å	Mo *K*α, 0.71073 Å
Temperature	100(2)	100(2)	100(2)
Crystal system	Triclinic	Monoclinic	Monoclinic
Space group	$P\bar{1}$ (No. 2)	$P2_1/n$ (No. 14)	$P2_1/n$ (No. 14)
Unit cell dimensions	*a* = 5.7616(3) Å, *b* = 13.6469(7) Å, *c* = 14.1530(8) Å, α = 110.433(2)°, β = 99.132(2)°, γ = 98.681(2)°	*a* = 12.2730(6) Å, *b* = 9.3005(4) Å, *c* = 15.0153(7) Å, β = 108.7040(10)°	*a* = 13.5083(7) Å, *b* = 8.6113(4) Å, *c* = 15.3635(8) Å, β = 99.227(2)°
Volume	1003.65(9) $Å^3$	1623.41(13) $Å^3$	1764.02(15) $Å^3$
Z and *Z*′	2 and 1	4 and 1	4 and 1
Calculated density	1.470 g cm^{-3}	1.352 g cm^{-3}	1.402 g cm^{-3}
Absorption coefficient	0.196 mm^{-1}	0.182 mm^{-1}	0.185 mm^{-1}
F(000)	464	704	784
Crystal size	0.706 x 0.177 x 0.175 mm^3	0.385 x 0.344 x 0.108 mm^3	0.520 x 0.513 x 0.340 mm^3
θ range for data collection	2.670° to 26.999°	2.617° to 27.000°	2.816° to 26.996°
Index ranges	$-7 \le h \le 7$, $-17 \le k \le 17$, $-18 \le l \le 18$	$-15 \le h \le 15$, $-11 \le k \le 11$, $-19 \le l \le 19$	$-17 \le h \le 17$, $-11 \le k \le 11$, $-19 \le l \le 19$
Reflections collected	44413	22695	32197
Independent reflections	4383	3540	3825
R_{int}	0.0497	0.0387	0.0325
R_σ	0.0242	0.0244	0.0172
Observed Data [$I > 2\sigma(I)$]	3979	3213	3589
Completeness to θ = 25.242°	99.8	99.6	99.4
Max. and min. transmission	0.7471 and 0.6352	0.7460 and 0.6872	0.7383 and 0.6952
Data / restraints / parameters	4383 / 0 / 284	3540 / 0 / 217	3825 / 0 / 243
Goodness-of-fit on F^2	1.035	1.061	1.051
Final *R* indices [$I > 2\sigma(I)$]	*R*1 = 0.0319, *wR*2 = 0.0807	*R*1 = 0.0357, *wR*2 = 0.0851	*R*1 = 0.0343, *wR*2 = 0.0903
R indices (all data)	*R*1 = 0.0354, *wR*2 = 0.0828	*R*1 = 0.0397, *wR*2 = 0.0873	*R*1 = 0.0366, *wR*2 = 0.0919
Largest diff. peak and hole	0.512 and –0.389 e $Å^{-3}$	0.430 and –0.339 e $Å^{-3}$	0.443 and –0.426 e $Å^{-3}$
CCDC number	2299351	2321868	2299352

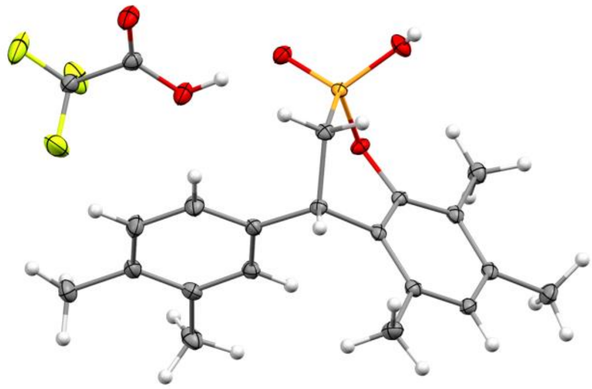

Figure S1. Molecular geometry in the crystals **2b**. Thermal ellipsoids for non-H atoms are set at the 50% probability level.

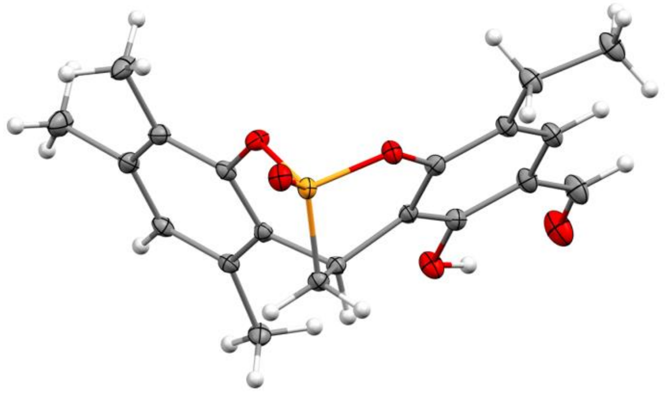

Figure S2. Molecular geometry in the crystals **2c**. Thermal ellipsoids for non-H atoms are set at the 50% probability level.

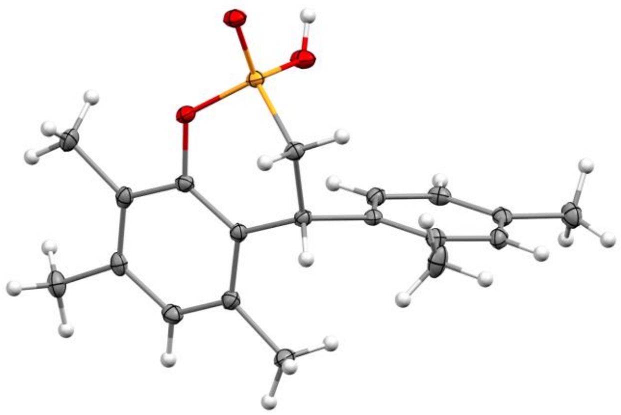

Figure S3. Molecular geometry in the crystals **3**. Thermal ellipsoids for non-H atoms are set at the 50% probability level.

Quantum chemistry calculations

Initially, we chose a range-separated ωB97X-V functional[6] both for the geometry optimization and thermochemistry calculations. The choice was mainly guided by the ability of ωB97X-V functional to reproduce non-covalent interaction reasonably well.[7] However, further studies demonstrated that the use of PBE functional[8] for geometry optimizations gives almost the same results, while being computationally much less demanding. The differences between relative free Gibbs energies were less than 1 kcal/mol (0.2 kcal/mol for transition state **TS1** and 0.6 kcal/mol for the intermediate **IM1**, see Scheme S1). Thus, we switched to the PBE functional for geometry optimizations, and used ωB97X-V functional for subsequent single point calculations.

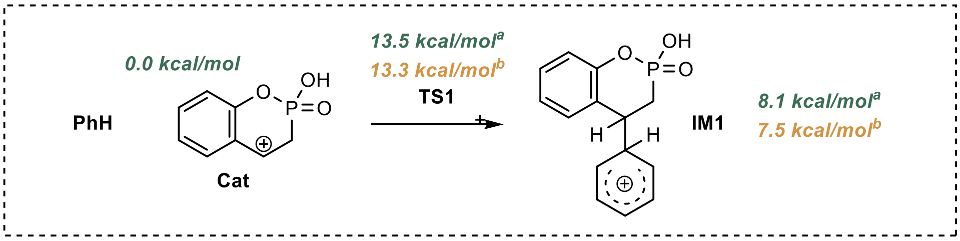

Scheme S1. Free Gibbs energies of reactants, transition state and intermediate for the reaction of the cation **Cat** with benzene calculated at different levels of theory. [a] ωB97X-V/def2-TZVPD//PBE/def2-TZVPD, C-PCM(TFA); [b] ωB97X-V/def2-TZVPD, C-PCM(TFA); (Orca 5.0.3)

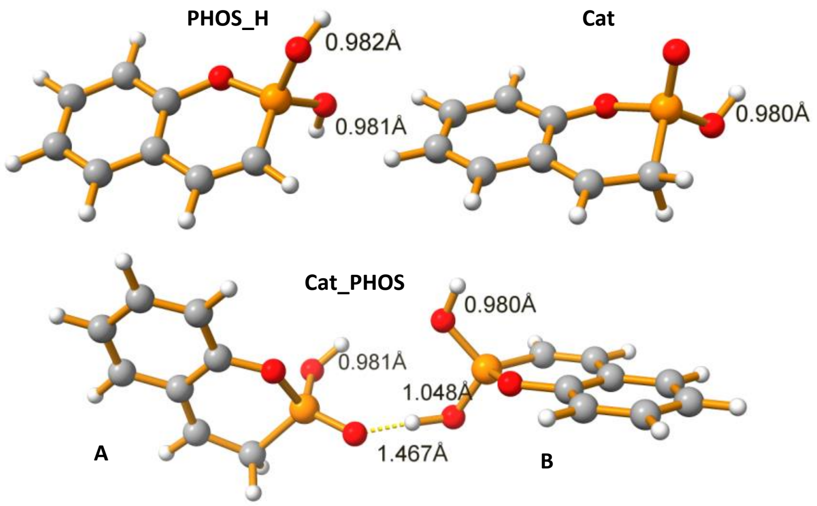

Figure S4. Calculated H…O bond lengths for the protonated phosphacoumarin **PHOS_H**, cation **Cat** and **Cat_PHOS** complex (ωB97X-V/def2-TZVPD//PBE/def2-TZVPD, C-PCM(TFA), Orca 5.0.3).

As seen from the Figure S4, H…O bond lengths are within 0.980-0.982 A for all three molecules, except the hydrogen atom involved in hydrogen bond in **Cat_PHOS** complex. The hydrogen atom is bonded to the molecule **B** of the complex (which is indeed the protonated phosphacoumarin **PHOS_H**). However, the bond is somewhat elongated compared to the non-complexed **PHOS_H** (1.048 vs 0.981 A), which is expected. The distance to the oxygen atom of the molecule **A** is much more (1.467 A). This is consistent with the **Cat_PHOS** being a complex of **Cat** and **PHOS_H**, rather than "truly" ditionic species.

Copies of NMR spectra

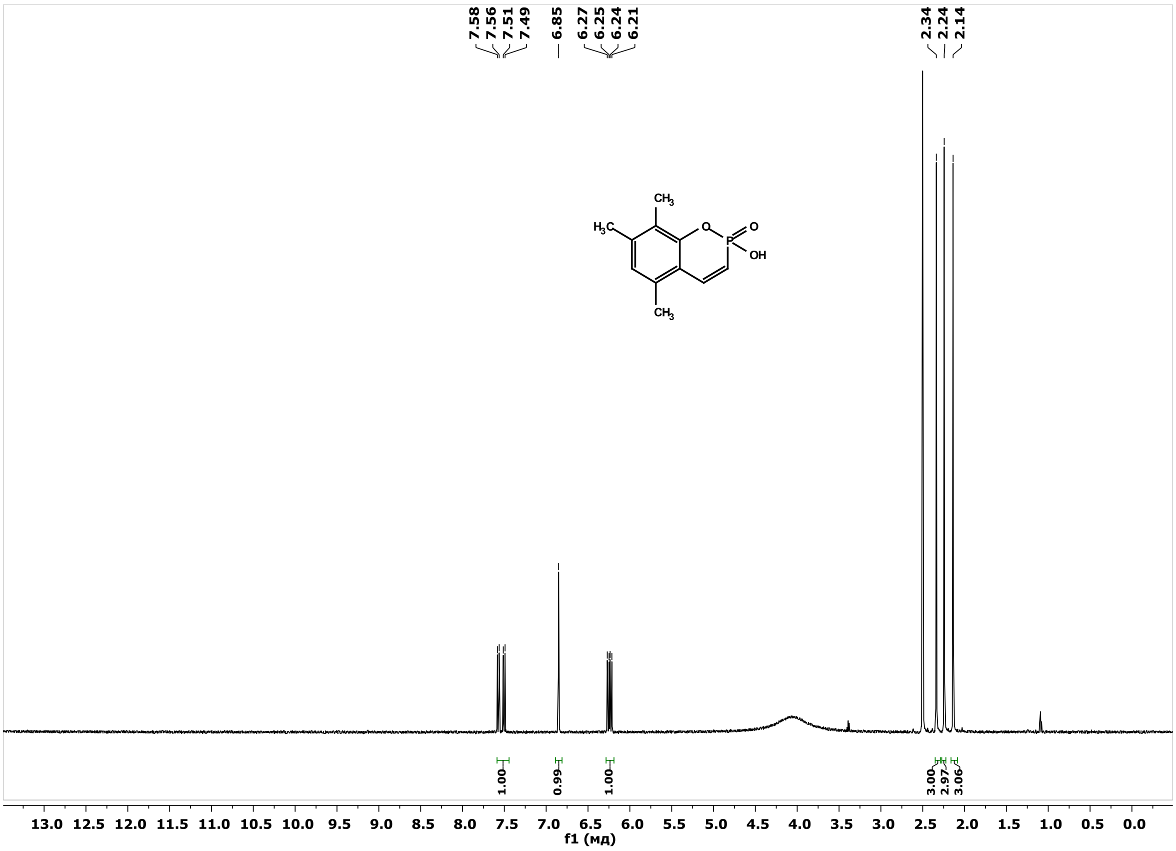

Figure S5. ^{1}H NMR (DMSO-d_6, 600 MHz) spectrum of the compound **1**

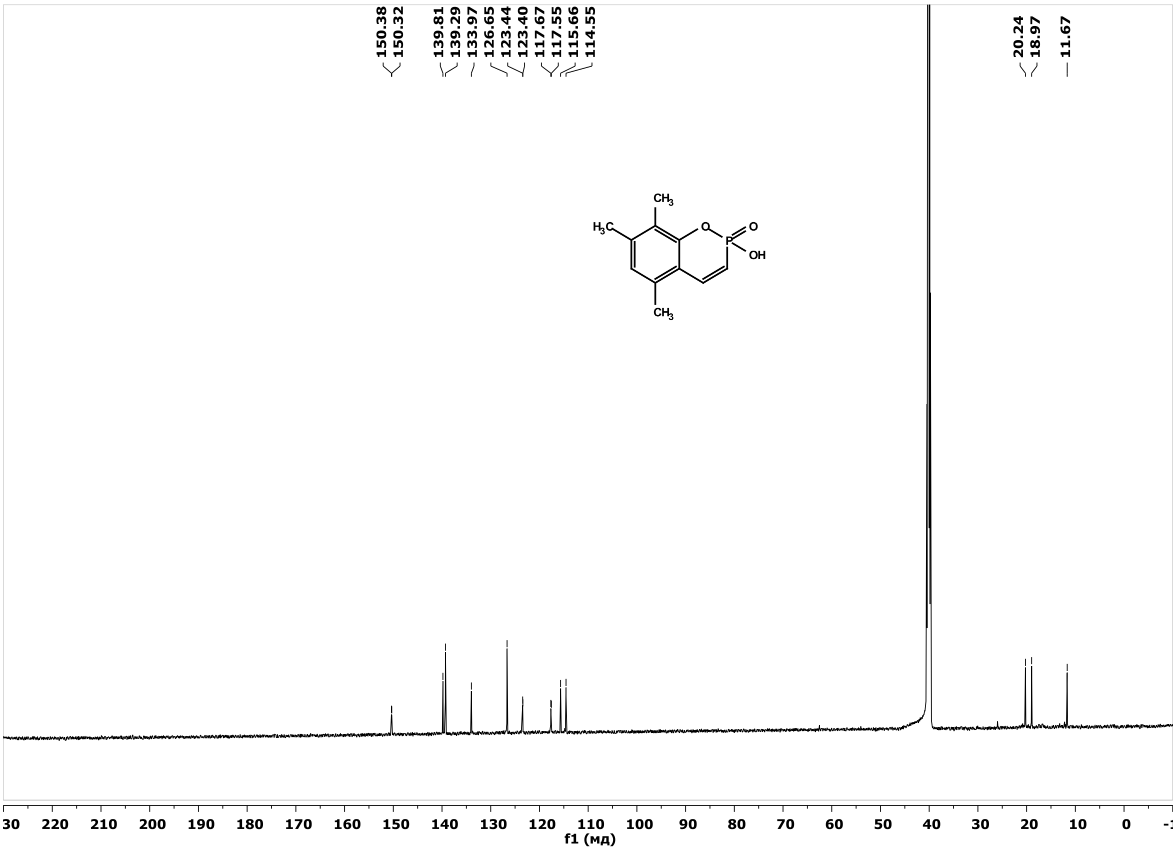

Figure S6. $^{13}C\{^{1}H\}$ NMR (DMSO-d_6, 151 MHz) spectrum of the compound **1**

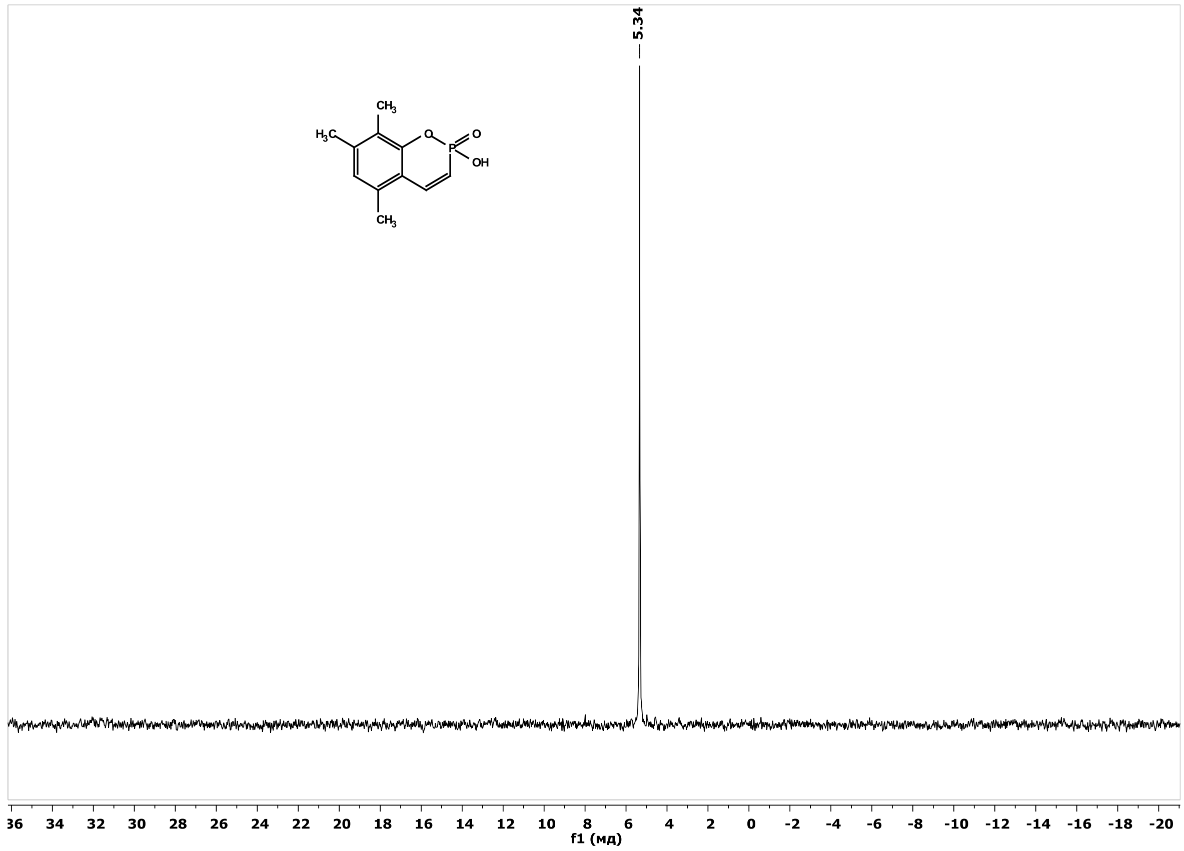

Figure S7. ^{31}P NMR (DMSO-d_6, 162 MHz) spectrum of the compound **1**

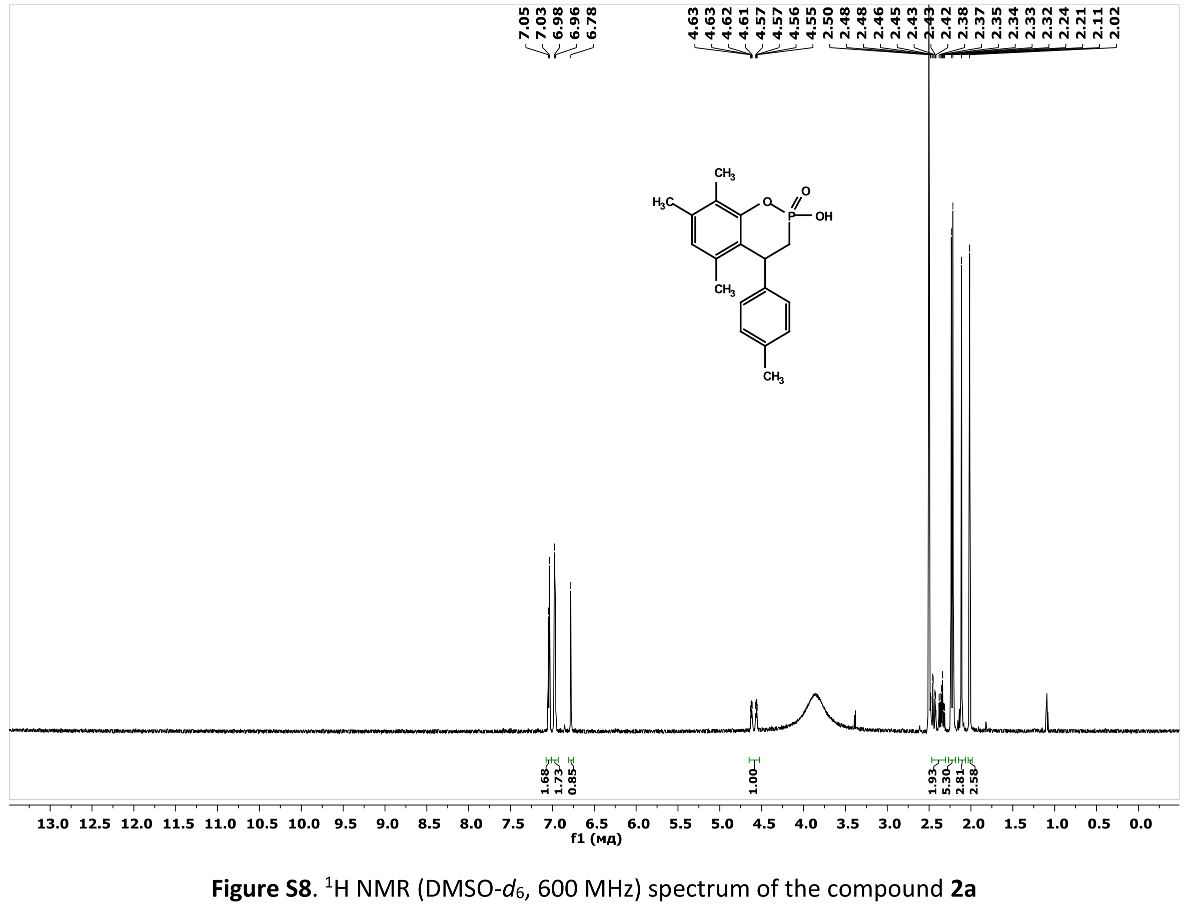

Figure S8. ^{1}H NMR (DMSO-d_6, 600 MHz) spectrum of the compound **2a**

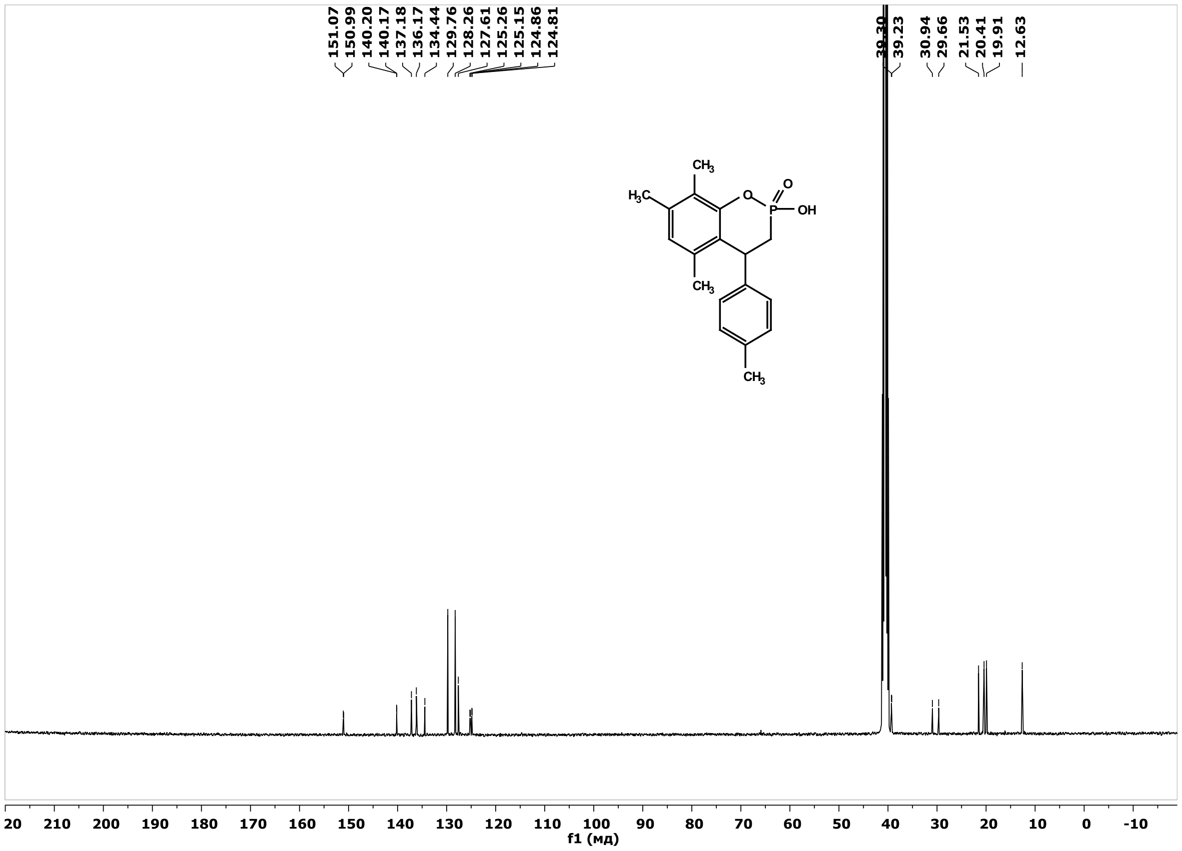

Figure S9. $^{13}C\{^{1}H\}$ NMR (DMSO-d_6, 101 MHz) spectrum of the compound **2a**

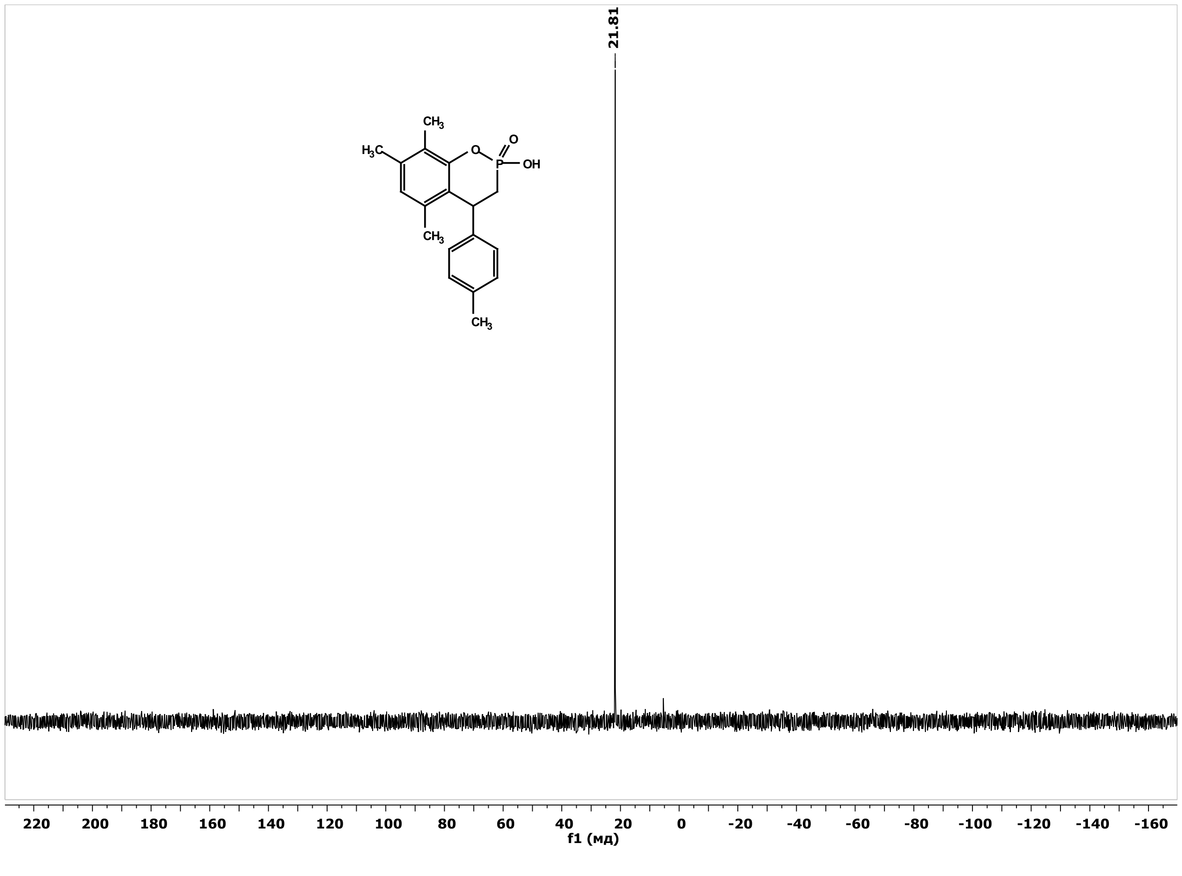

Figure S10. ^{31}P NMR (DMSO-d_6, 243 MHz) spectrum of the compound **2a**

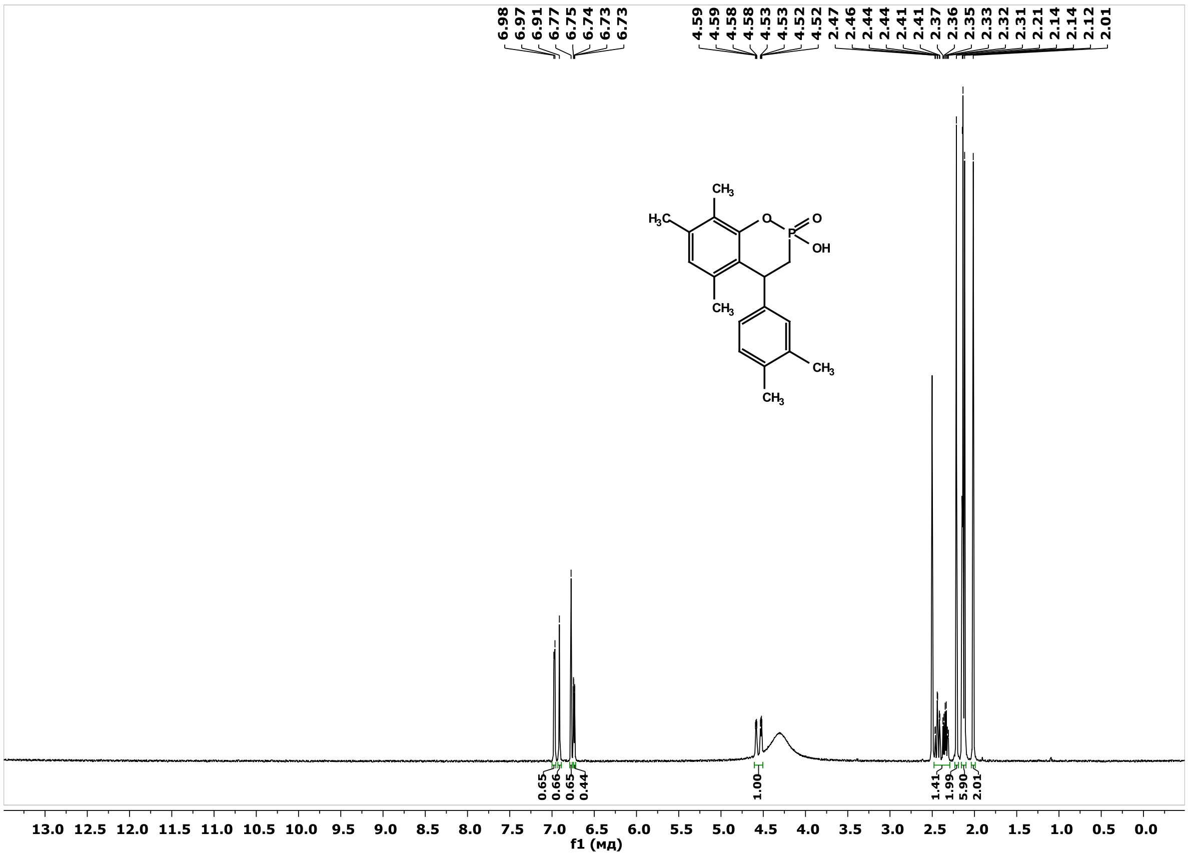

Figure S11. ^{1}H NMR (DMSO-d_6, 600 MHz) spectrum of the compound **2b**

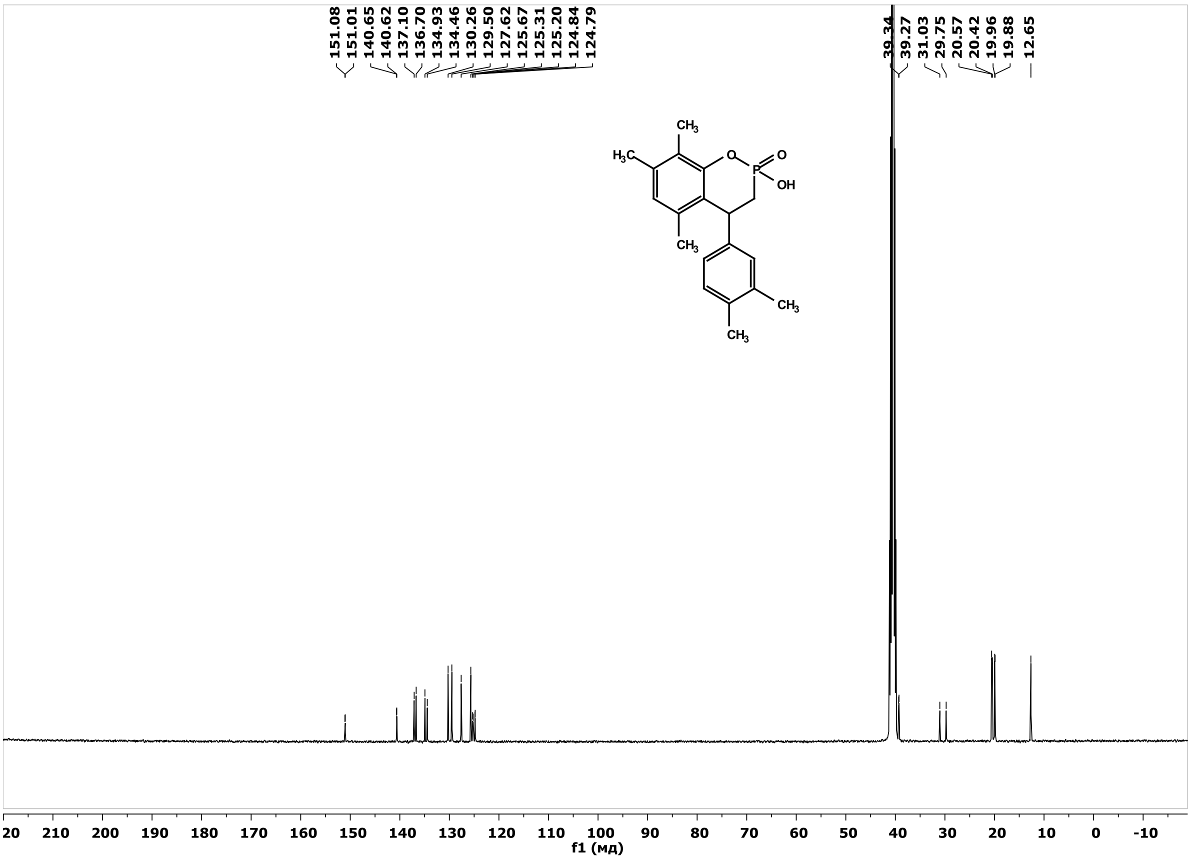

Figure S12. $^{13}C\{^{1}H\}$ NMR (DMSO-d_6, 101 MHz) spectrum of the compound **2b**

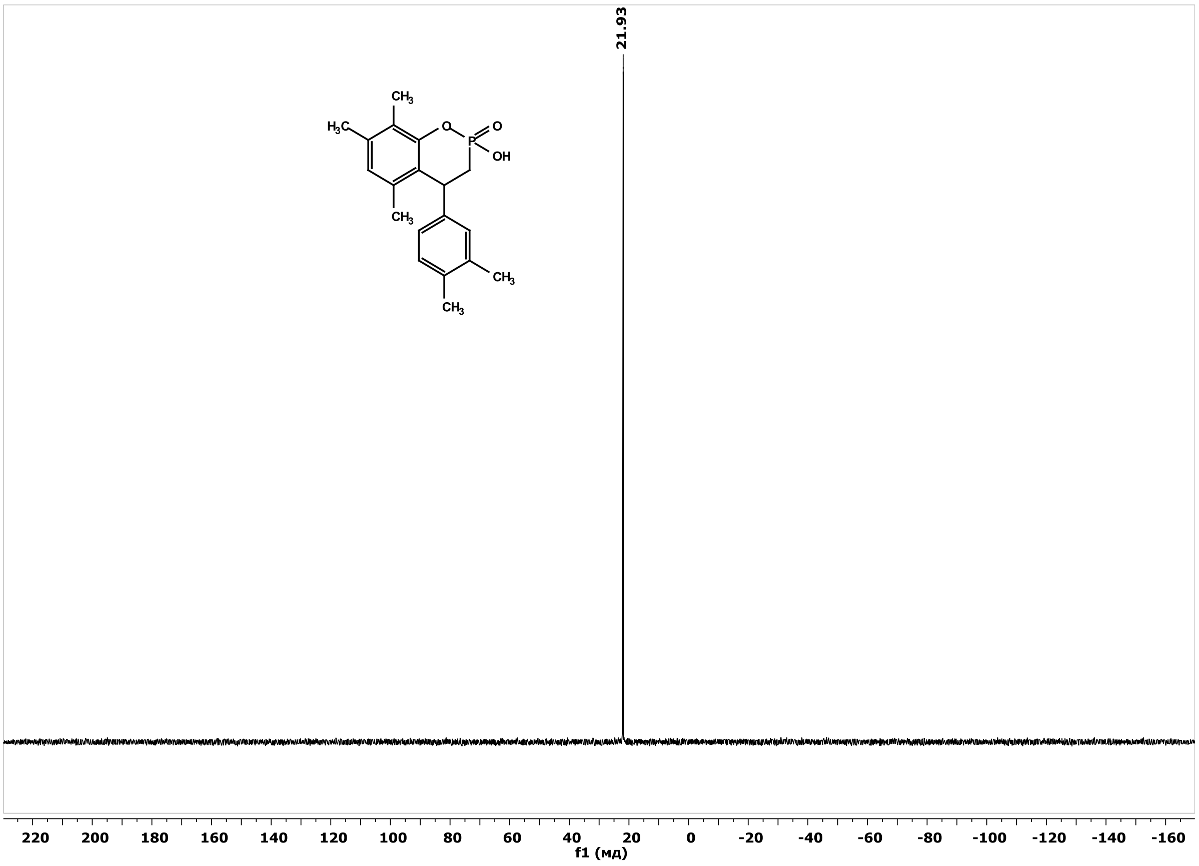

Figure S13. ^{31}P NMR (DMSO-d_6, 243 MHz) spectrum of the compound **2b**

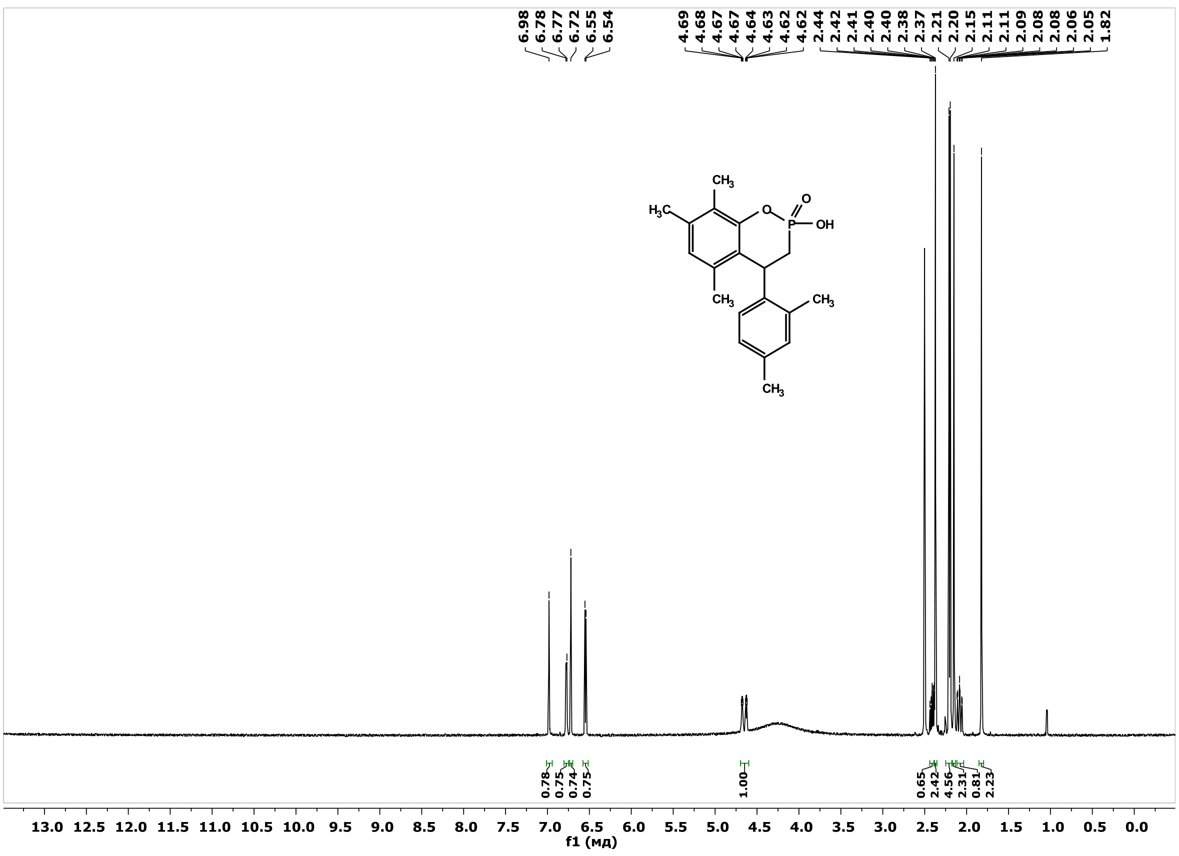

Figure S14. ^{1}H NMR (DMSO-d_6, 600 MHz) spectrum of the compound **2c**

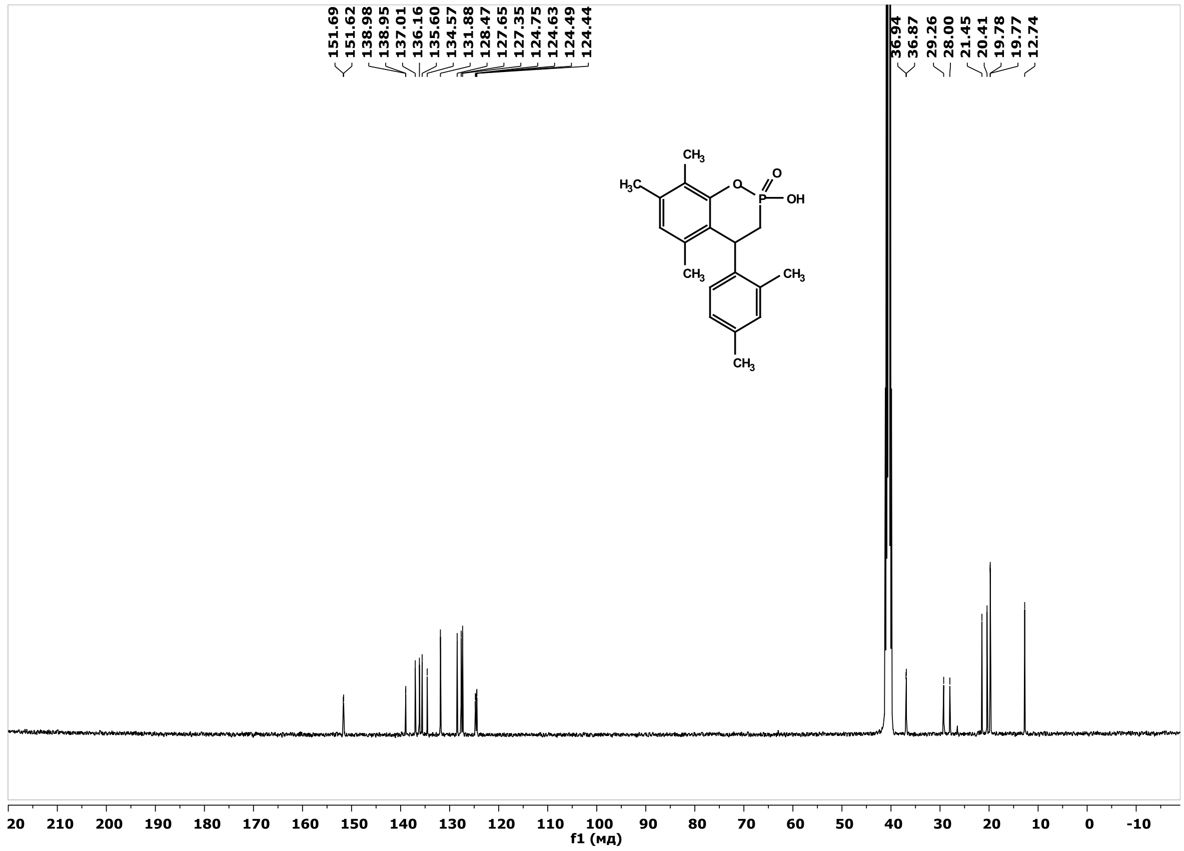

Figure S15. $^{13}C\{^{1}H\}$ NMR (DMSO-d_6, 101 MHz) spectrum of the compound **2c**

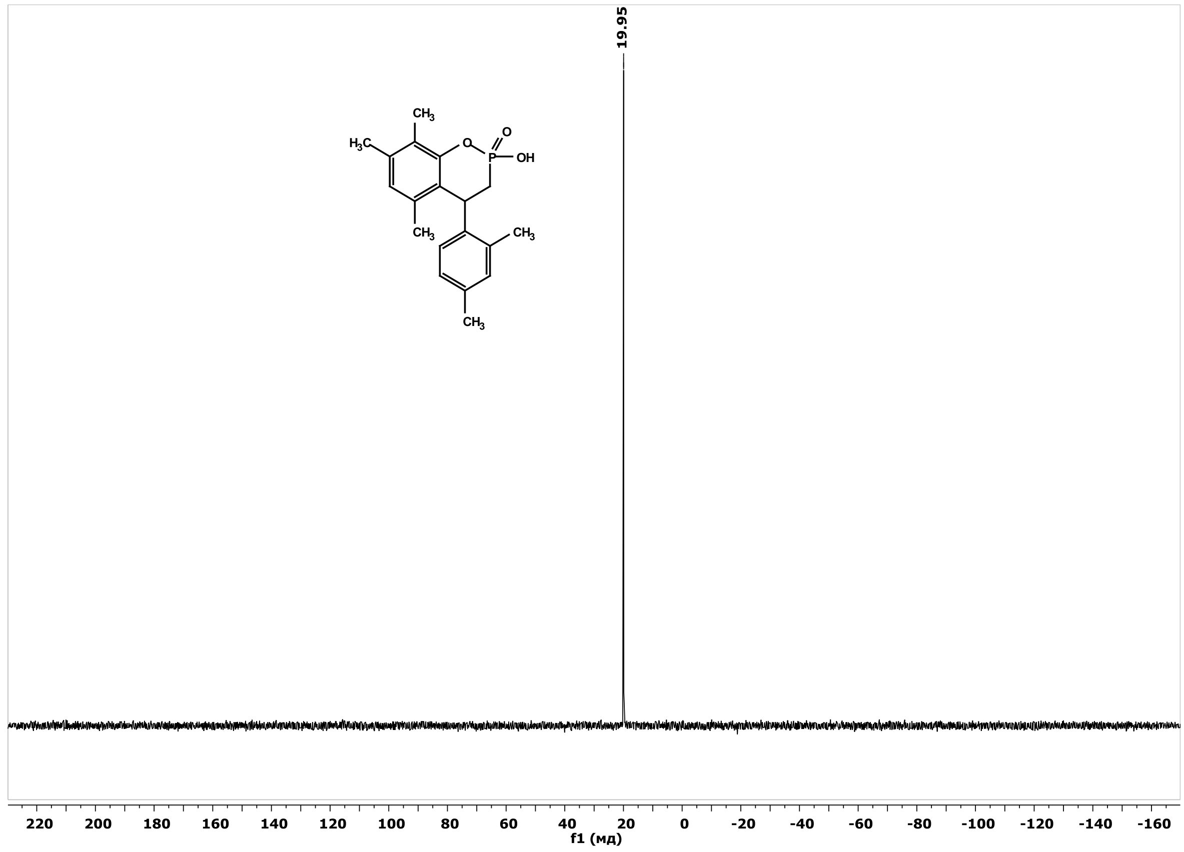

Figure S16. ^{31}P NMR (DMSO-d_6, 243 MHz) spectrum of the compound **2c**

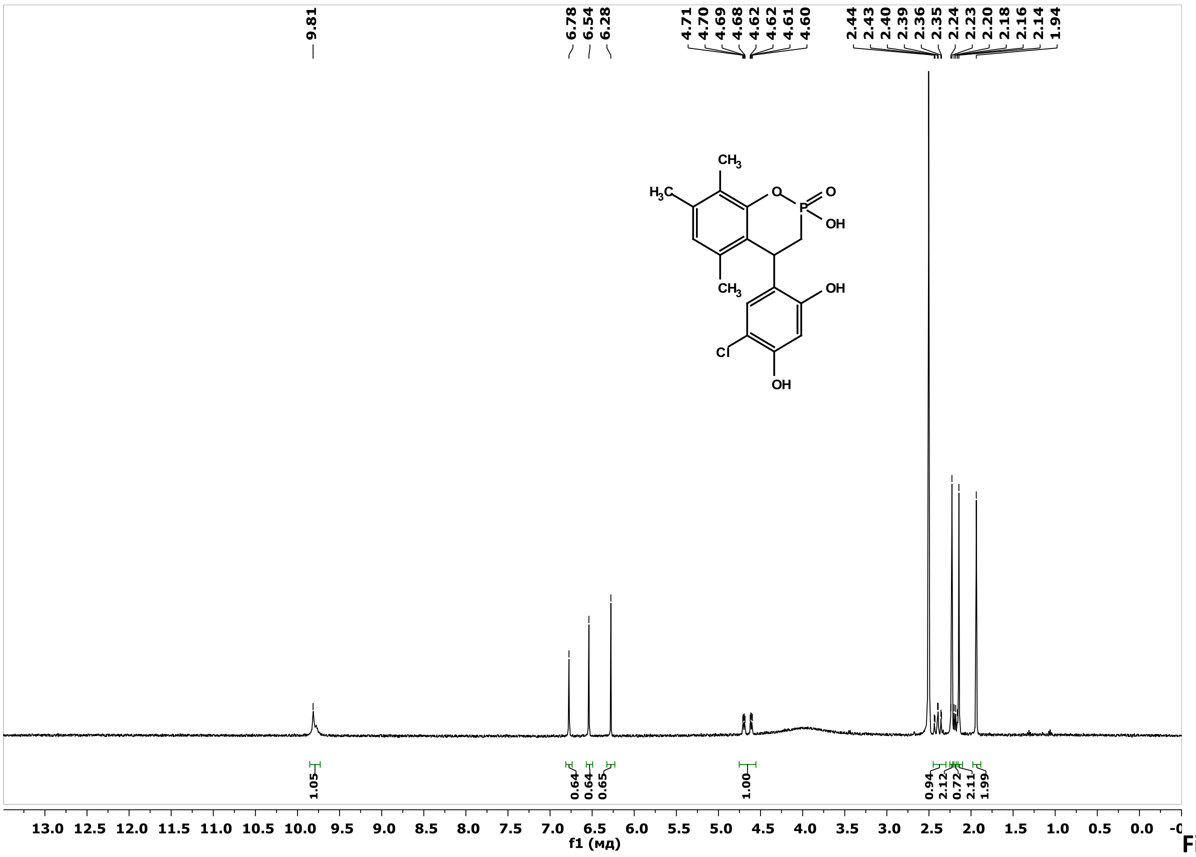

Figure S17. ^{1}H NMR (DMSO-d_6, 400 MHz) spectrum of the compound **2d**

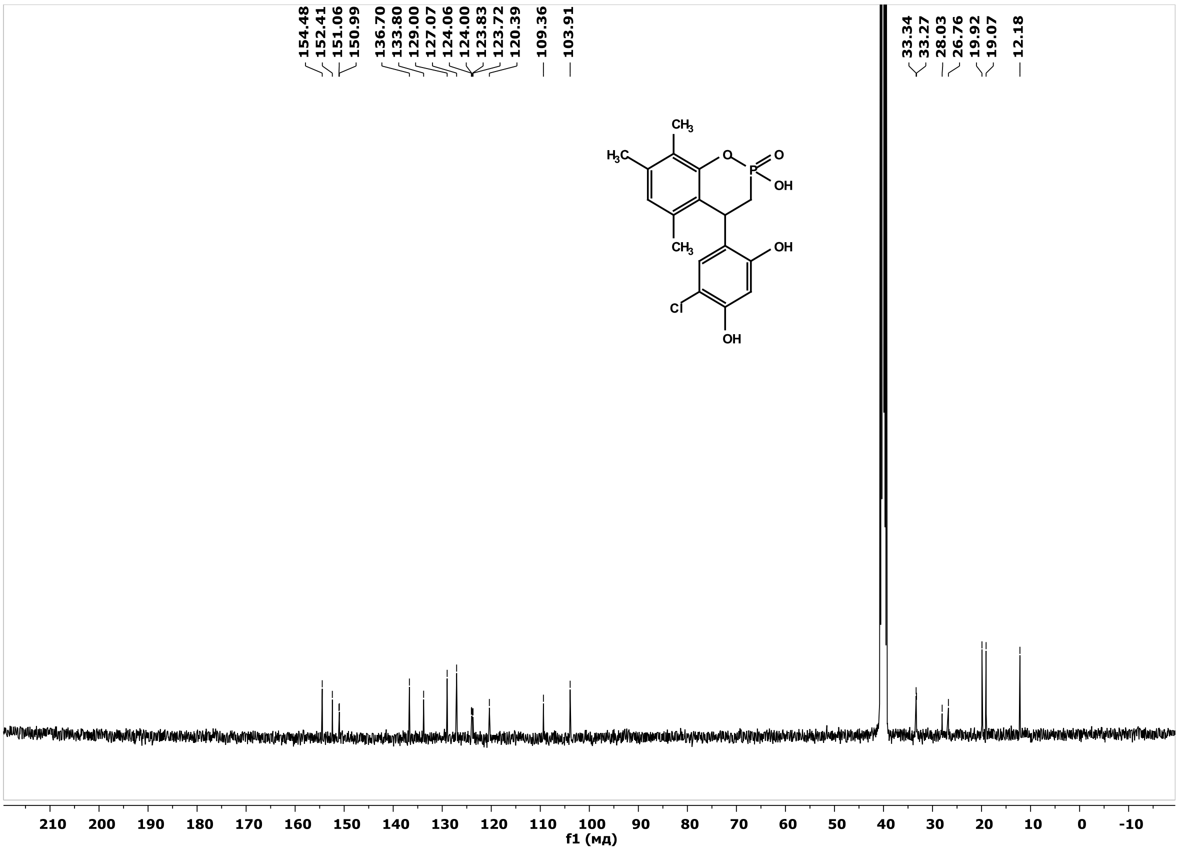

Figure S18. $^{13}C\{^{1}H\}$ NMR (DMSO-d_6, 101 MHz) spectrum of the compound **2d**

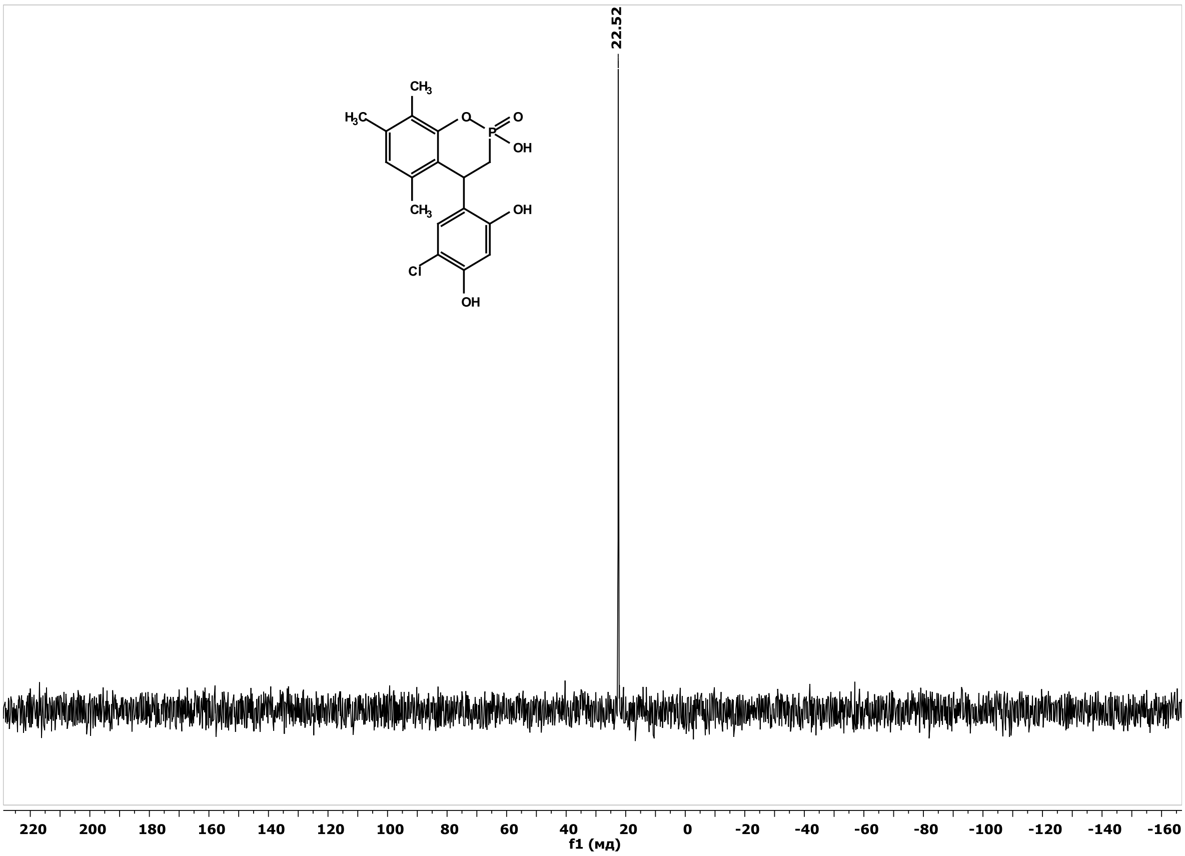

Figure S19. ^{31}P NMR (DMSO-d_6, 162 MHz) spectrum of the compound **2d**

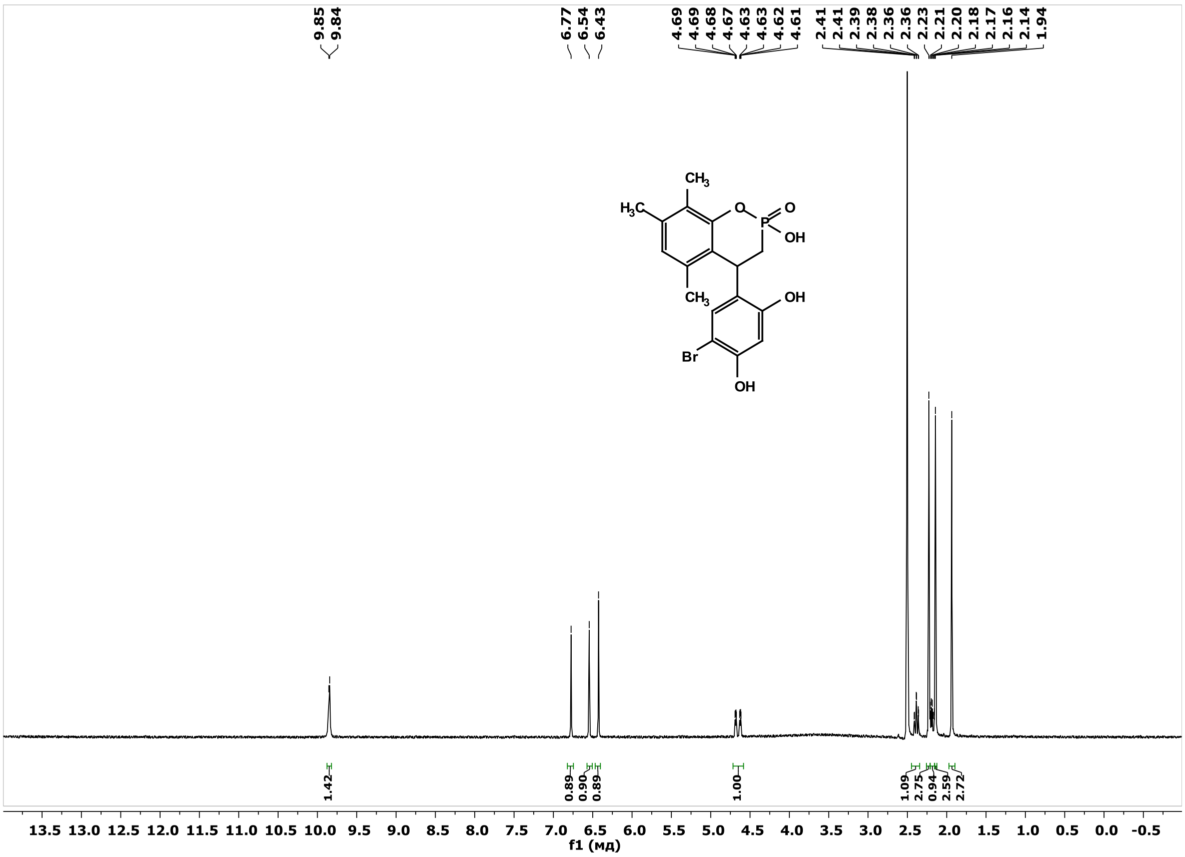

Figure S20. ^{1}H NMR (DMSO-d_6, 600 MHz) spectrum of the compound **2e**

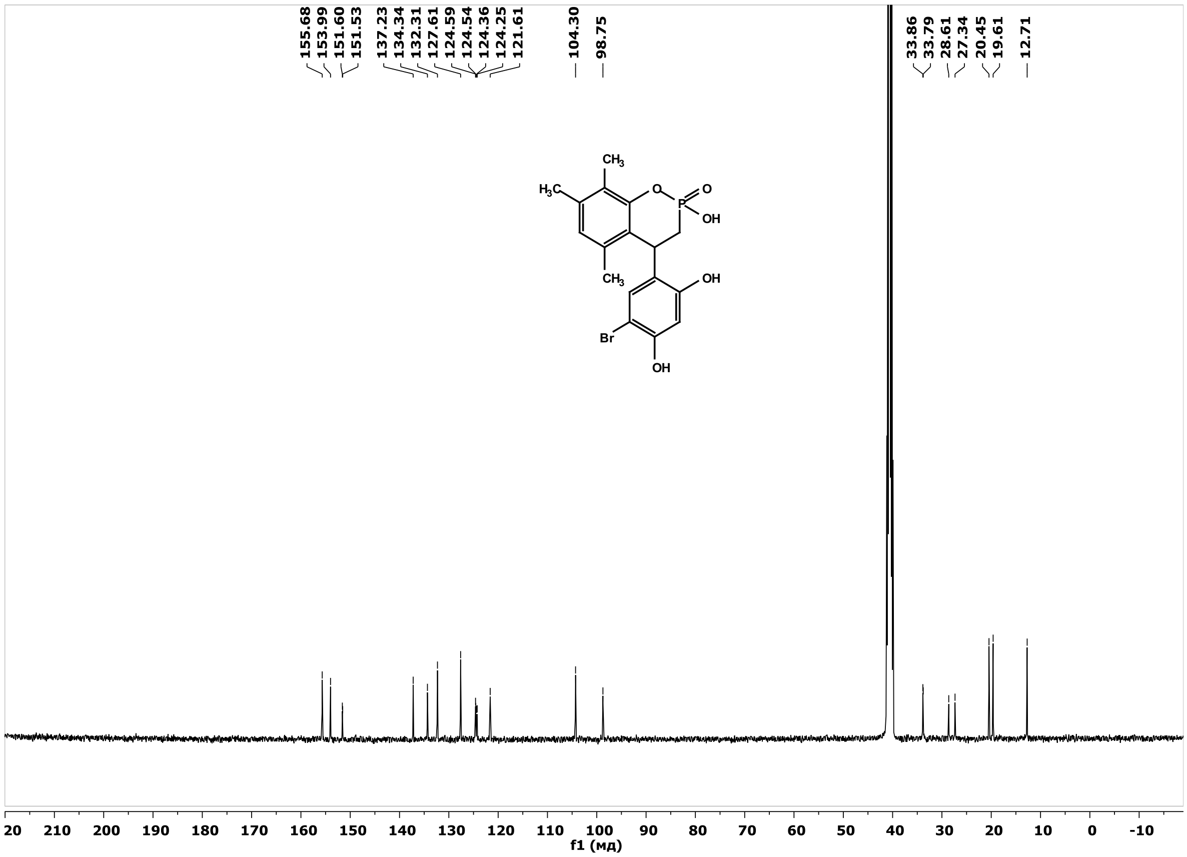

Figure S21. $^{13}C\{^{1}H\}$ NMR (DMSO-d_6, 101 MHz) spectrum of the compound **2e**

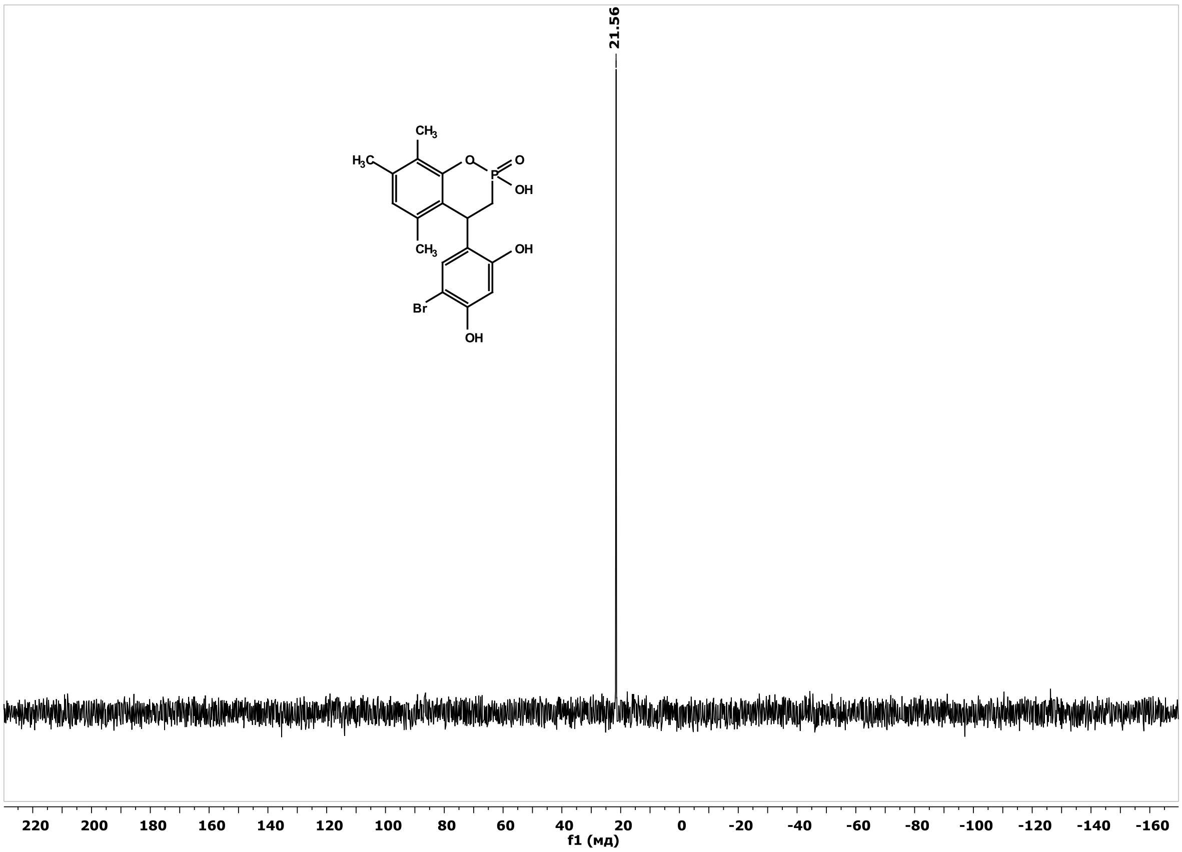

Figure S22. ^{31}P NMR (DMSO-d_6, 243 MHz) spectrum of the compound **2e**

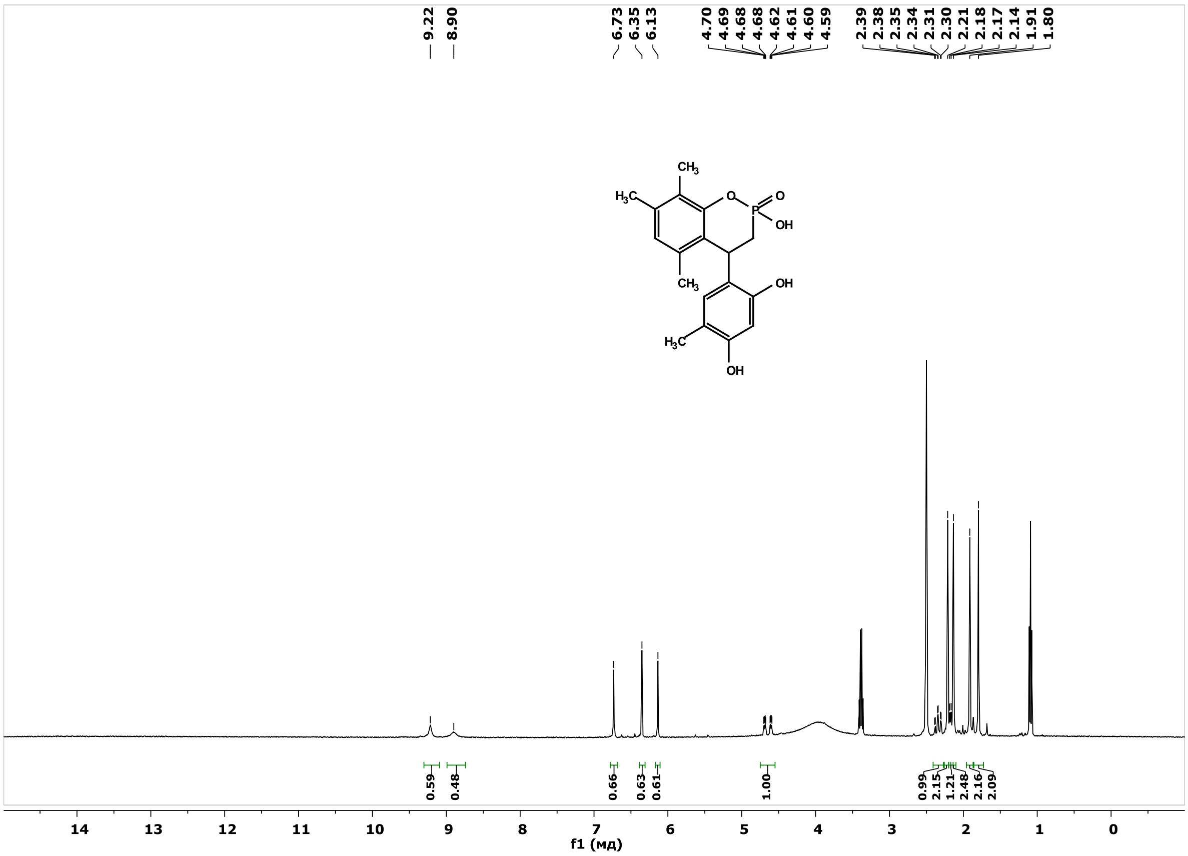

Figure S23. ^{1}H NMR (DMSO-d_6, 400 MHz) spectrum of the compound **2f**

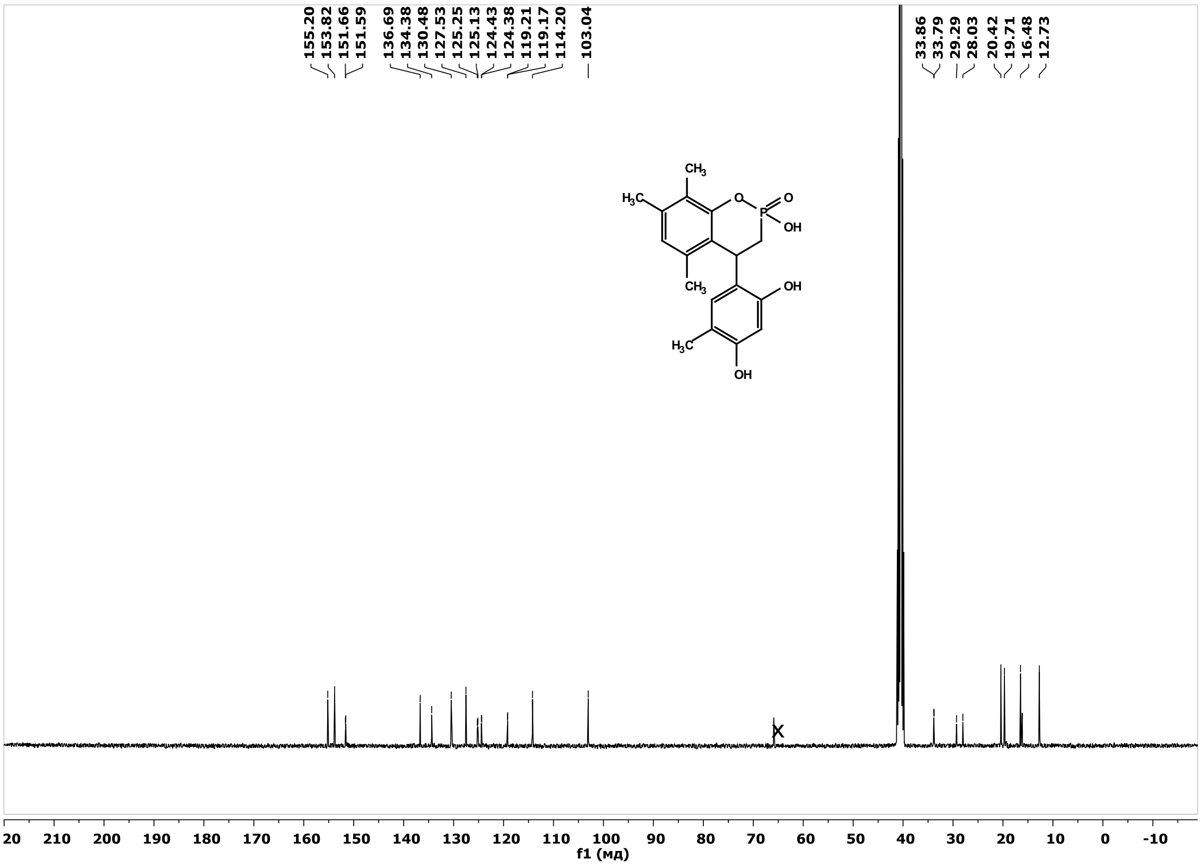

Figure S24. $^{13}C\{^{1}H\}$ NMR (DMSO-d_6, 101 MHz) spectrum of the compound **2f**

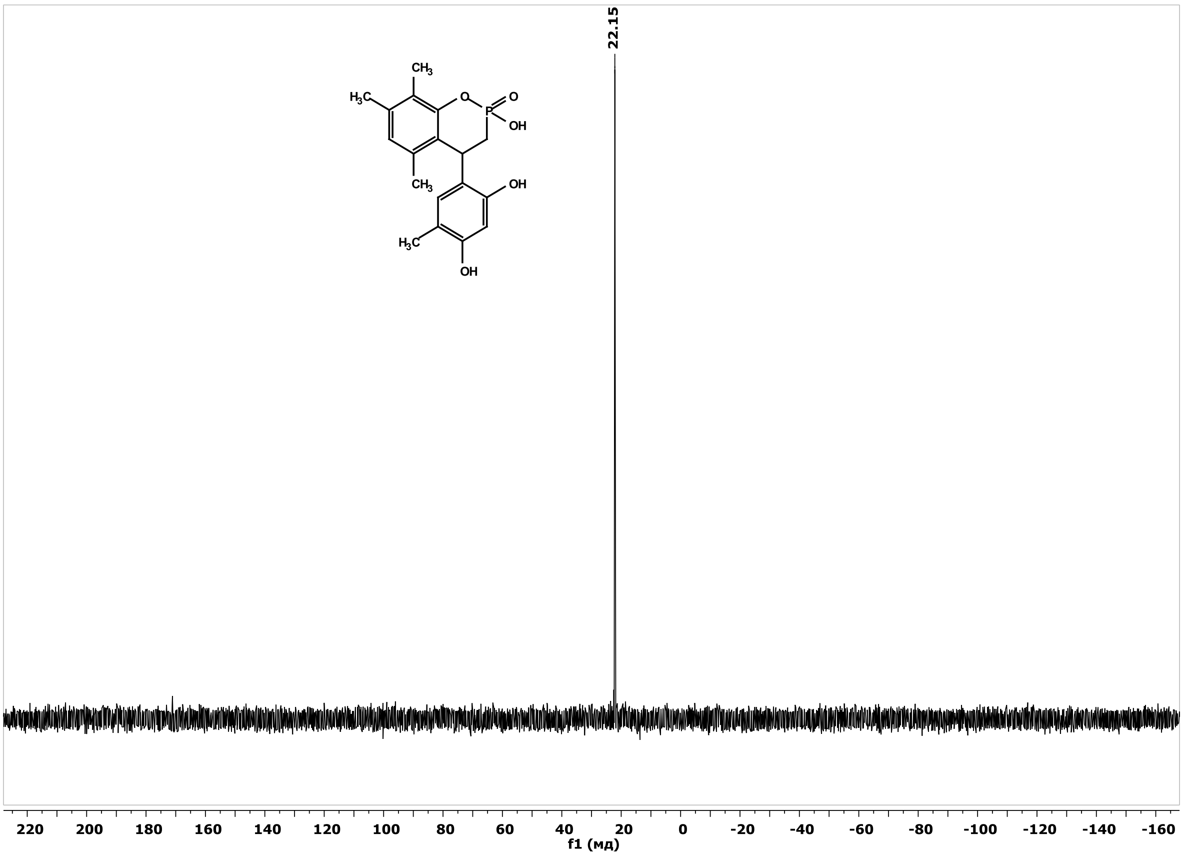

Figure S25. ^{31}P NMR (DMSO-d_6, 162 MHz) spectrum of the compound **2f**

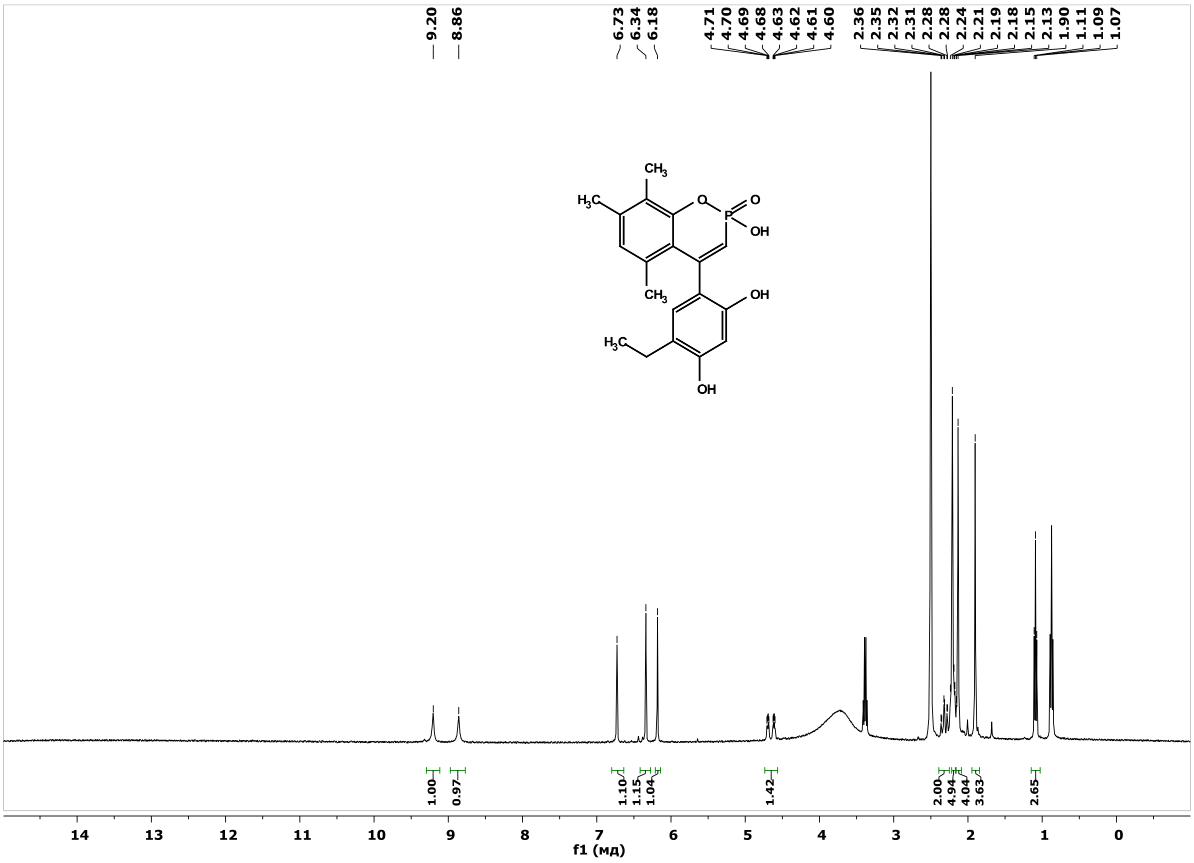

Figure S26. ^{1}H NMR (DMSO-d_6, 400 MHz) spectrum of the compound **2g**

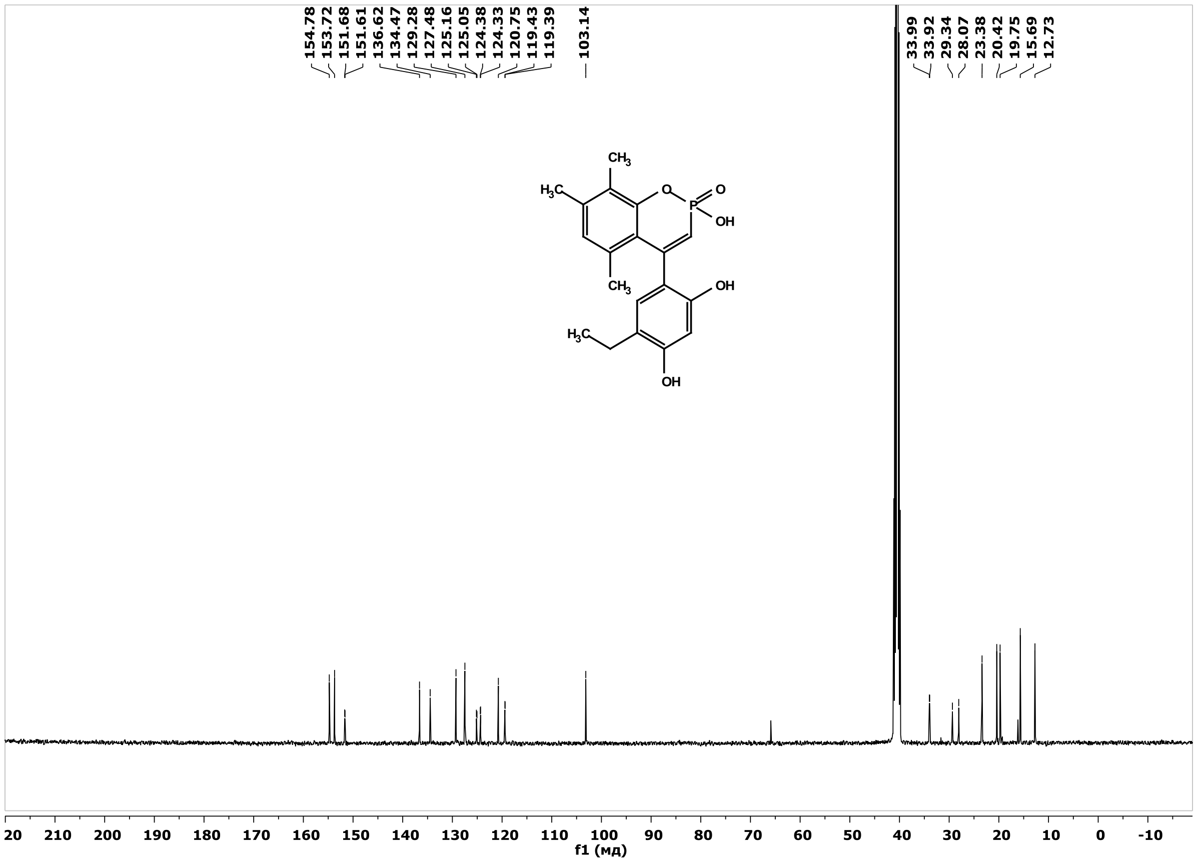

Figure S27. $^{13}C\{^{1}H\}$ NMR (DMSO-d_6, 101 MHz) spectrum of the compound **2g**

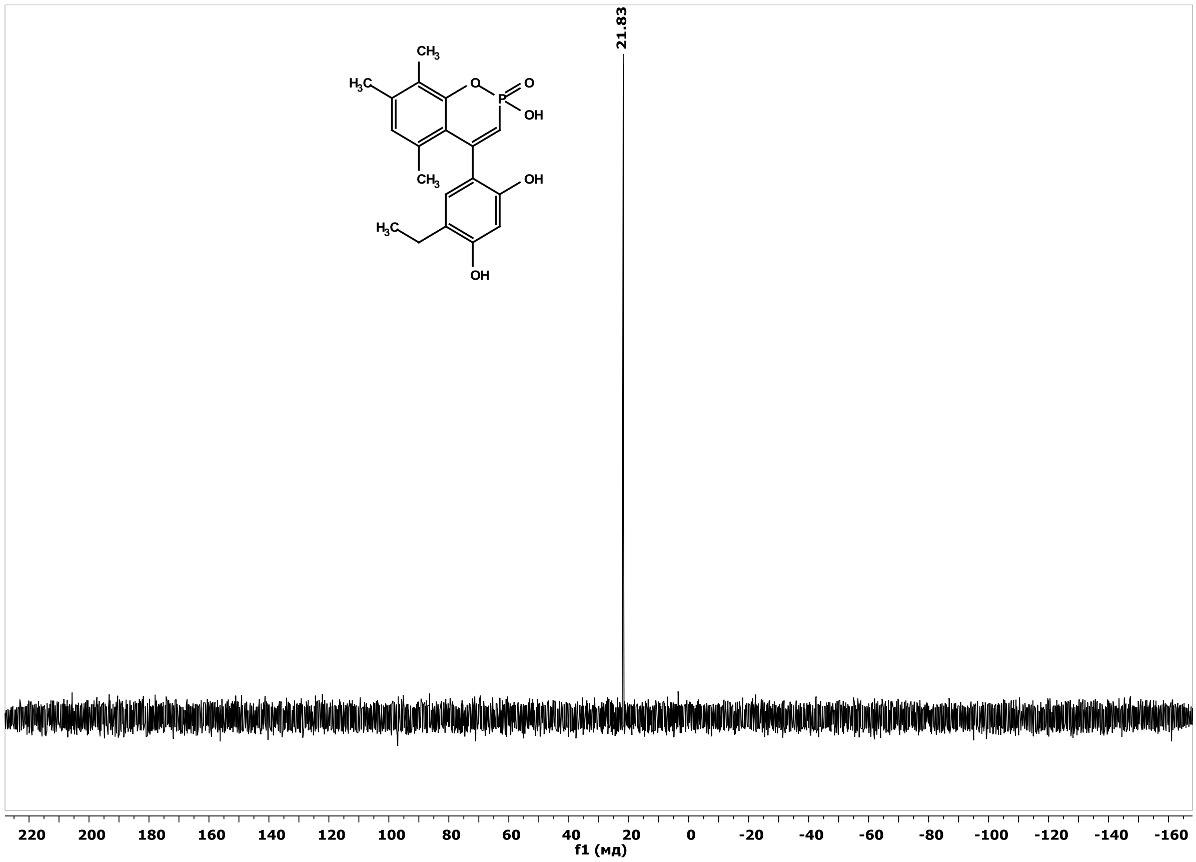

Figure S28. ^{31}P NMR (DMSO-d_6, 162 MHz) spectrum of the compound **2g**

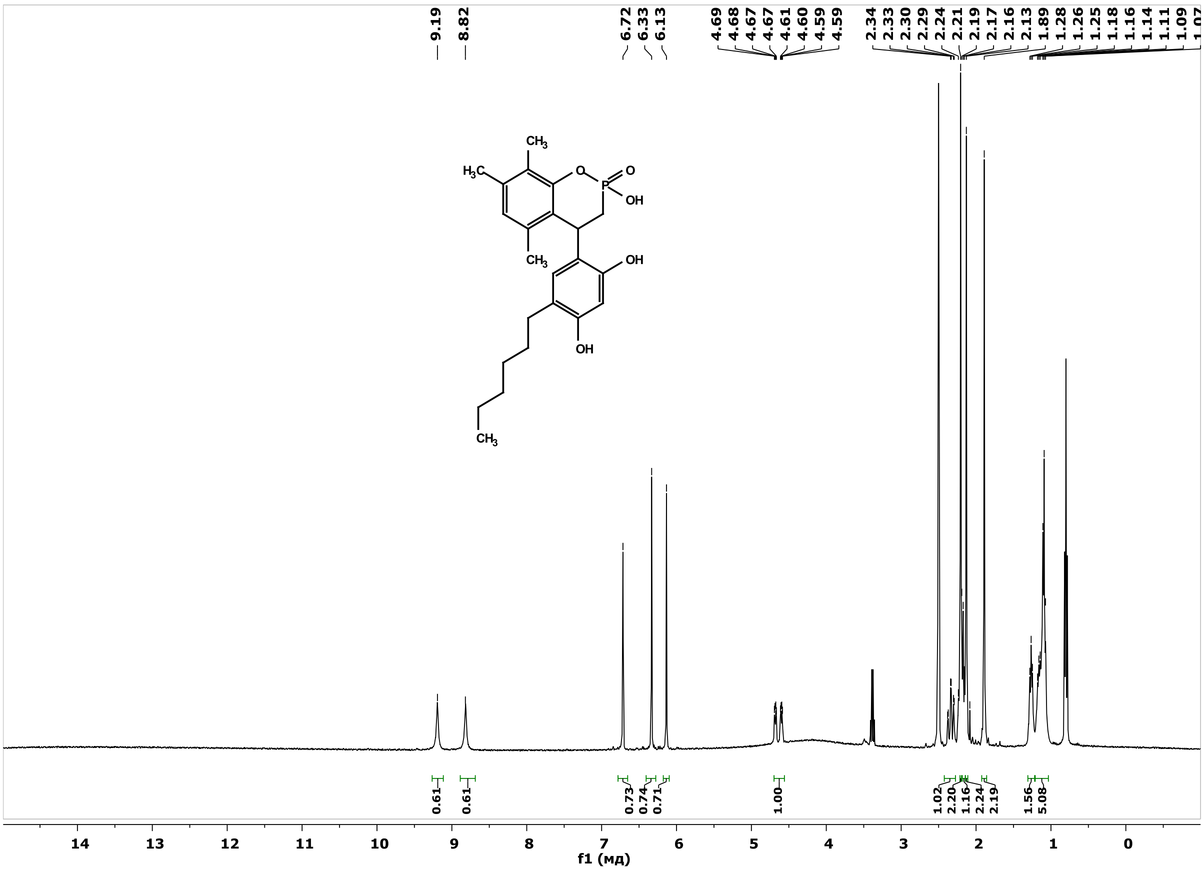

Figure S29. ^{1}H NMR (DMSO-d_6, 400 MHz) spectrum of the compound **2h**

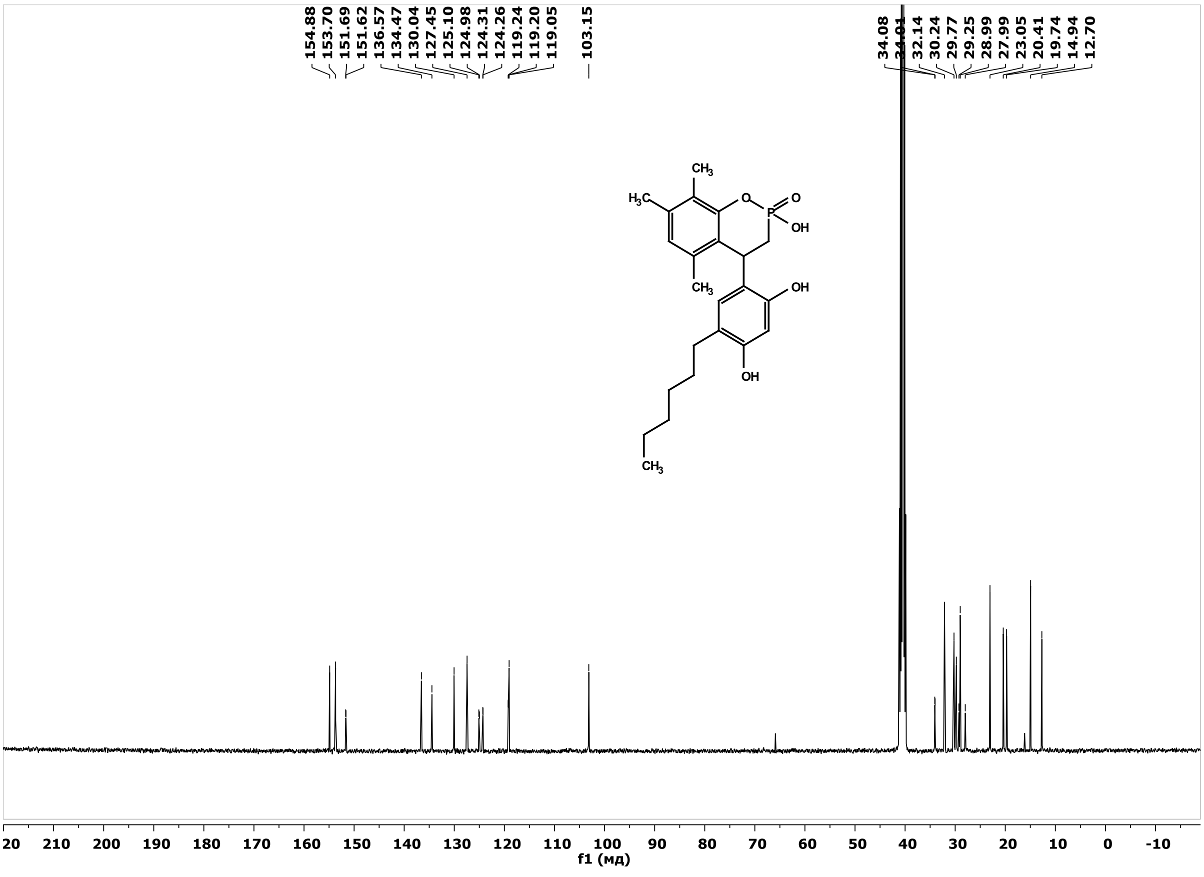

Figure S30. ^{13}C{^{1}H} NMR (DMSO-d_6, 101 MHz) spectrum of the compound **2h**

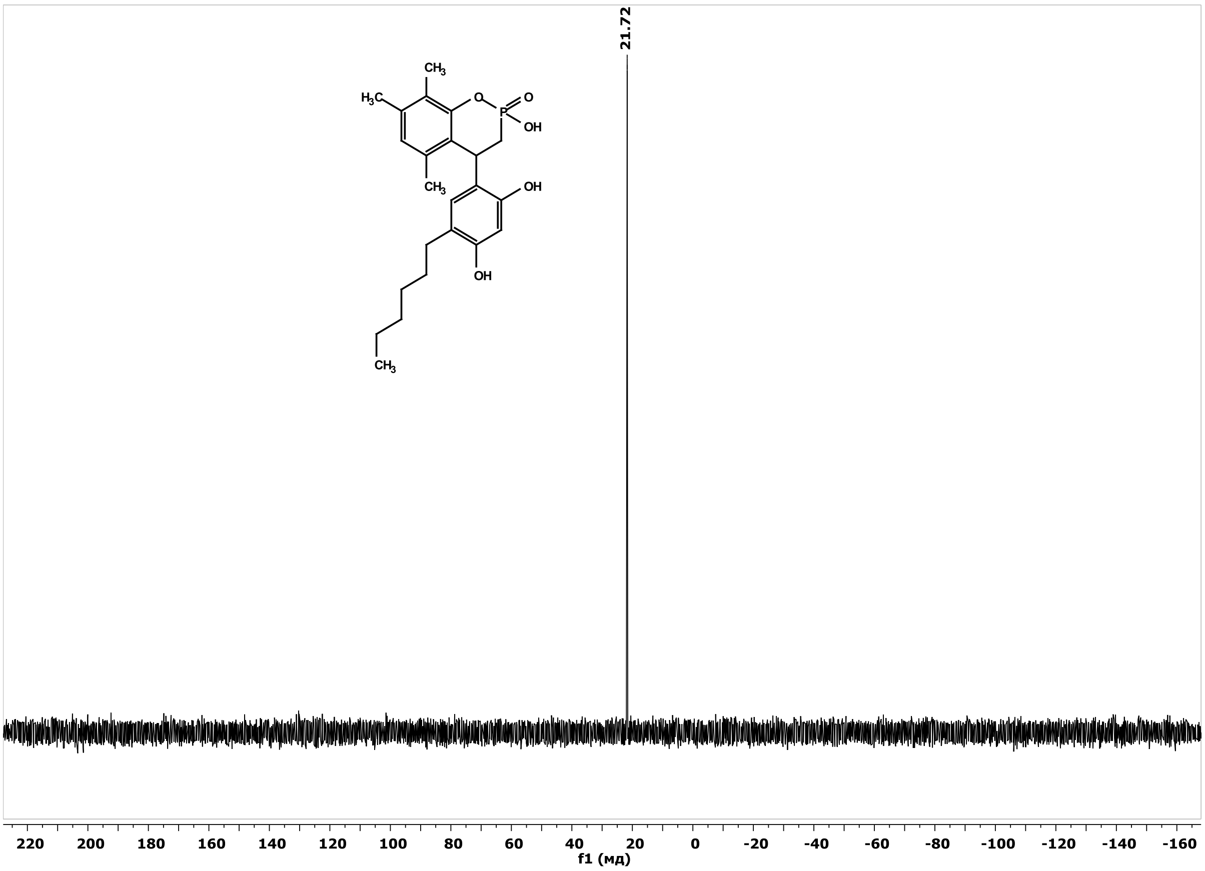

Figure S31. ^{31}P NMR (DMSO-d_6, 162 MHz) spectrum of the compound **2h**

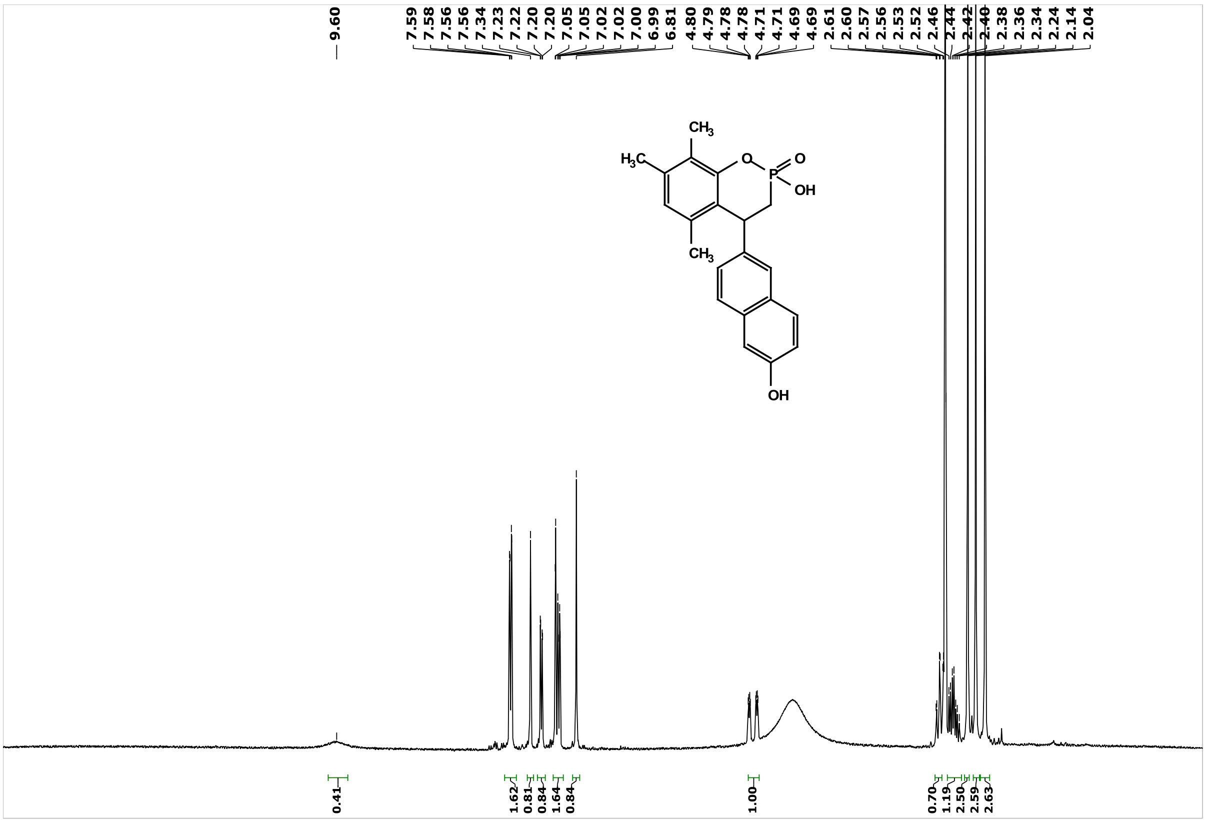

Figure S32. ^{1}H NMR (DMSO-d_6, 400 MHz) spectrum of the compound **2i**

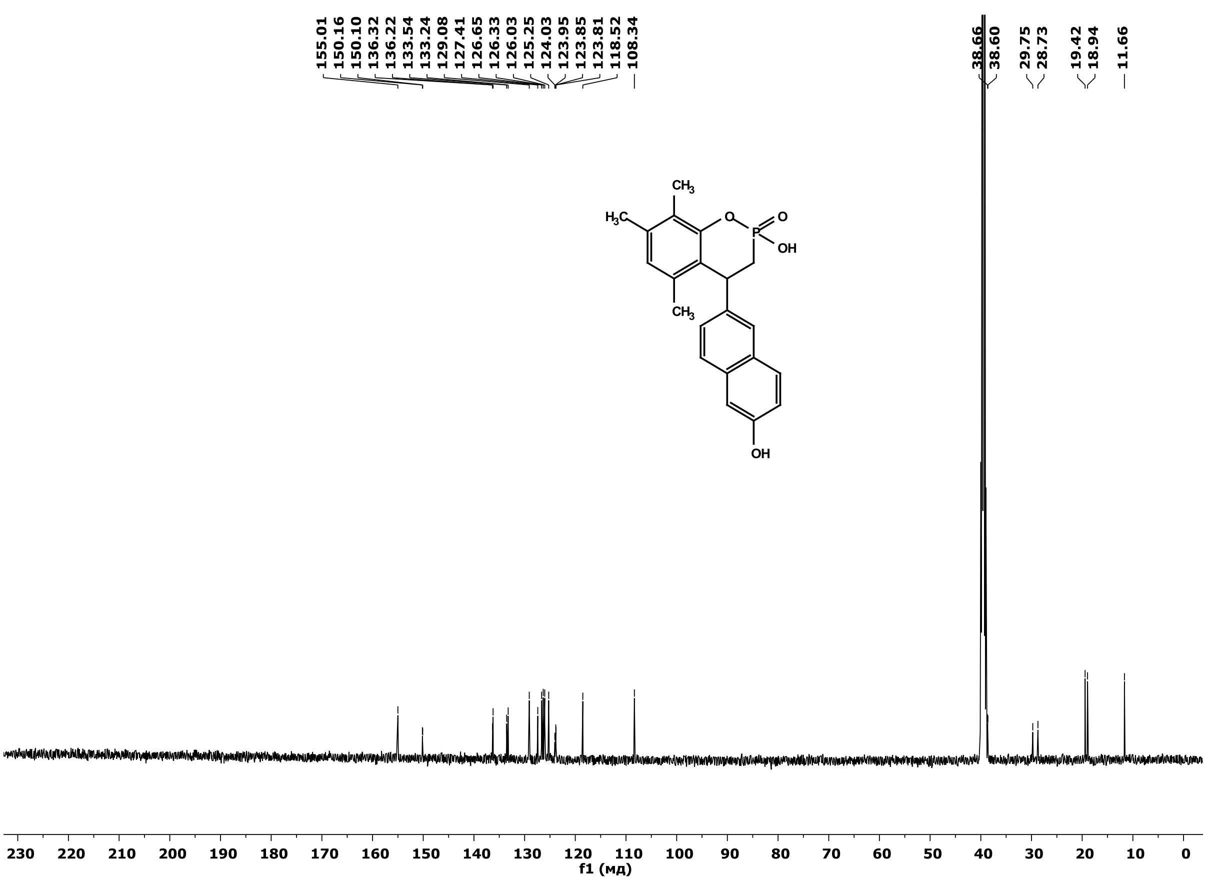

Figure S33. $^{13}C\{^1H\}$ NMR (DMSO-*d*6, 126 MHz) spectrum of the compound **2i**

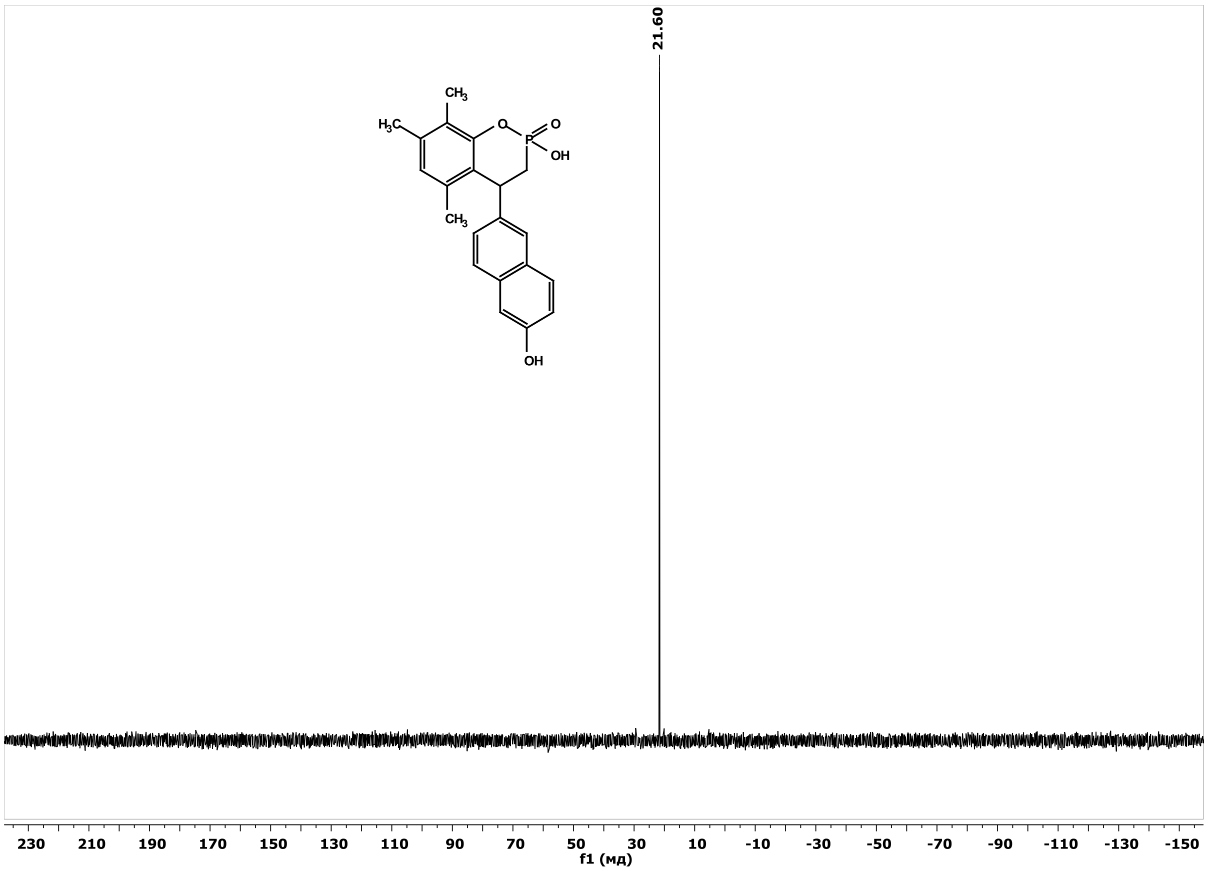

Figure S34. ^{31}P NMR (DMSO-*d*6, 162 MHz) spectrum of the compound **2i**

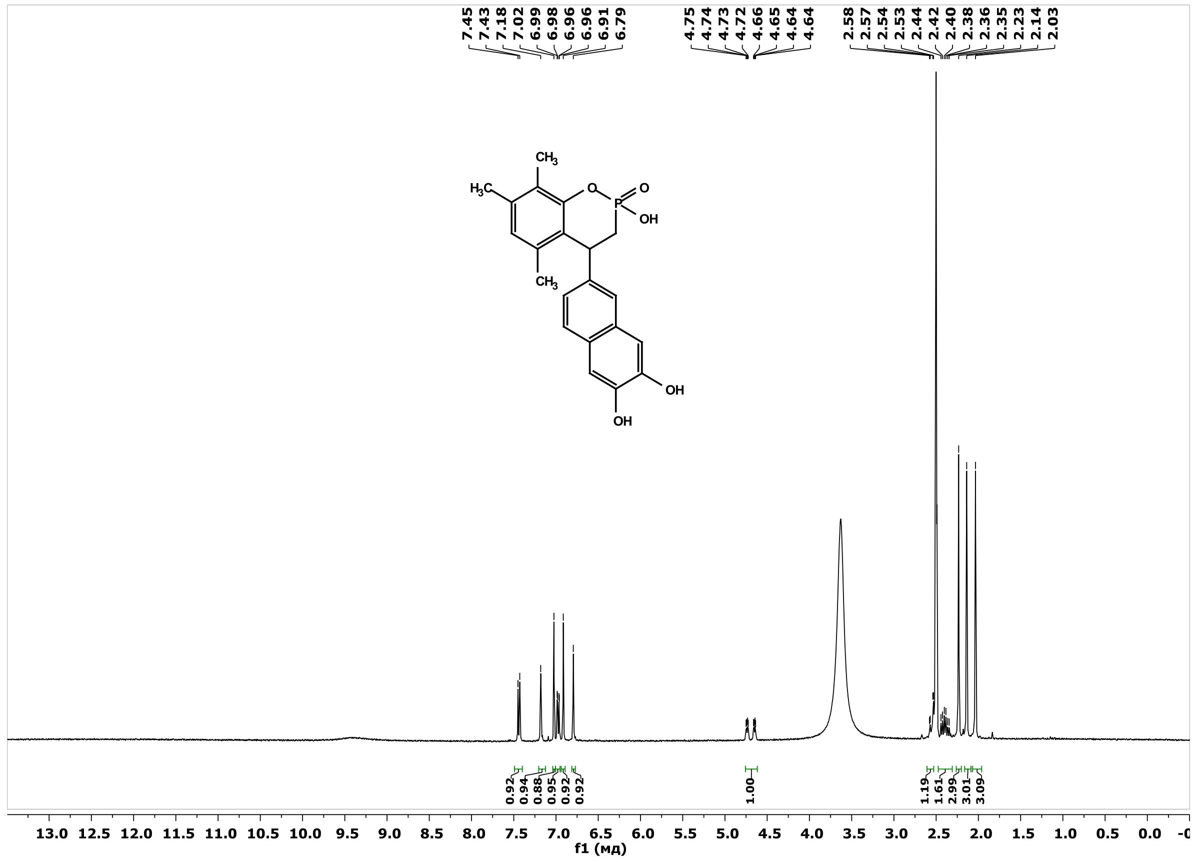

Figure S35. ^{1}H NMR (DMSO-d_6, 400 MHz) spectrum of the compound **2j**

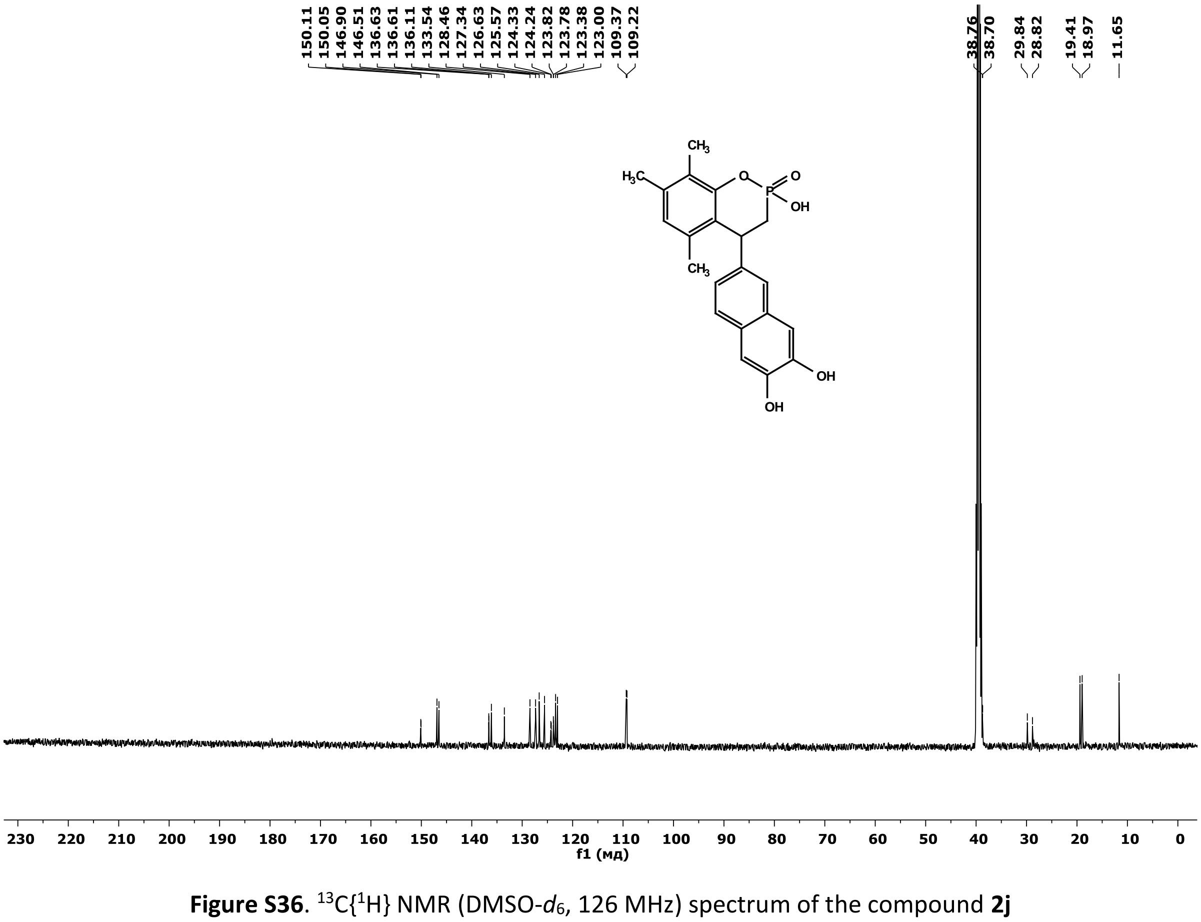

Figure S36. $^{13}C\{^{1}H\}$ NMR (DMSO-d_6, 126 MHz) spectrum of the compound **2j**

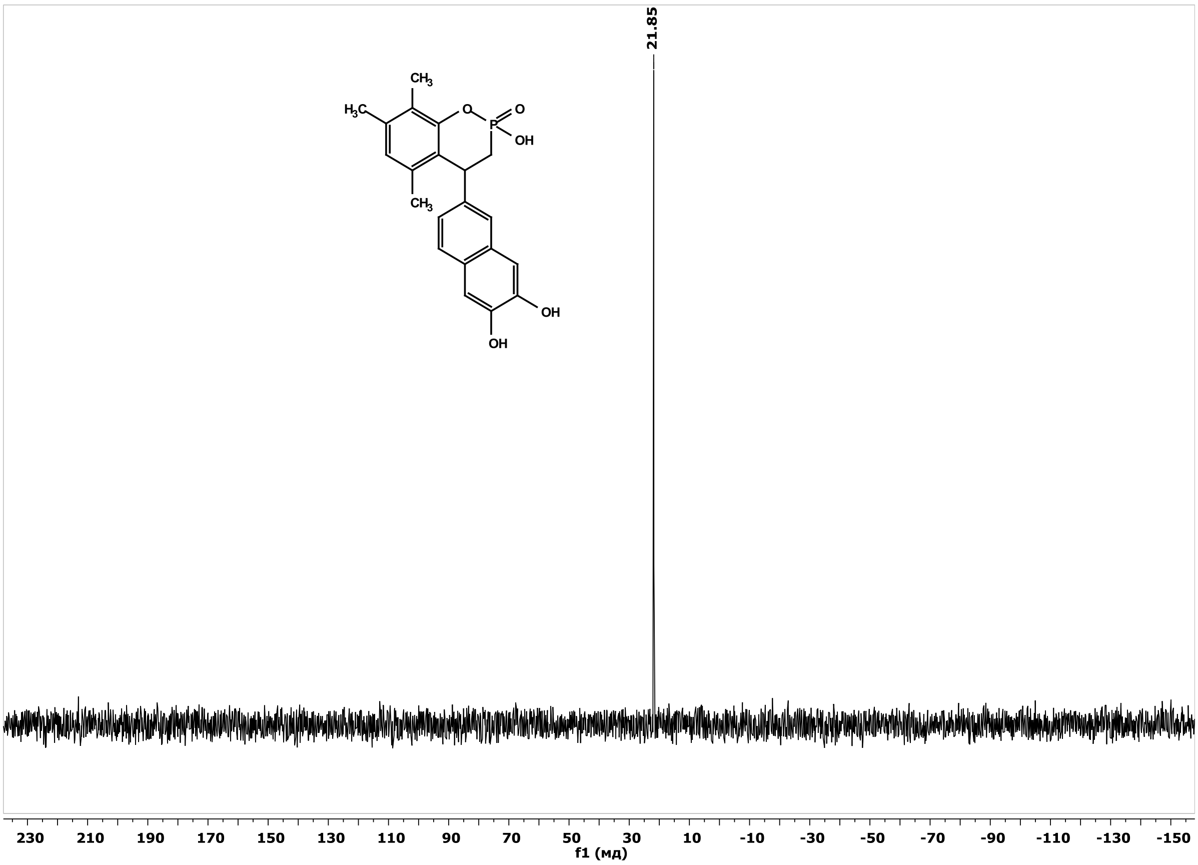

Figure S37. ^{31}P NMR (DMSO-d_6, 162 MHz) spectrum of the compound **2j**

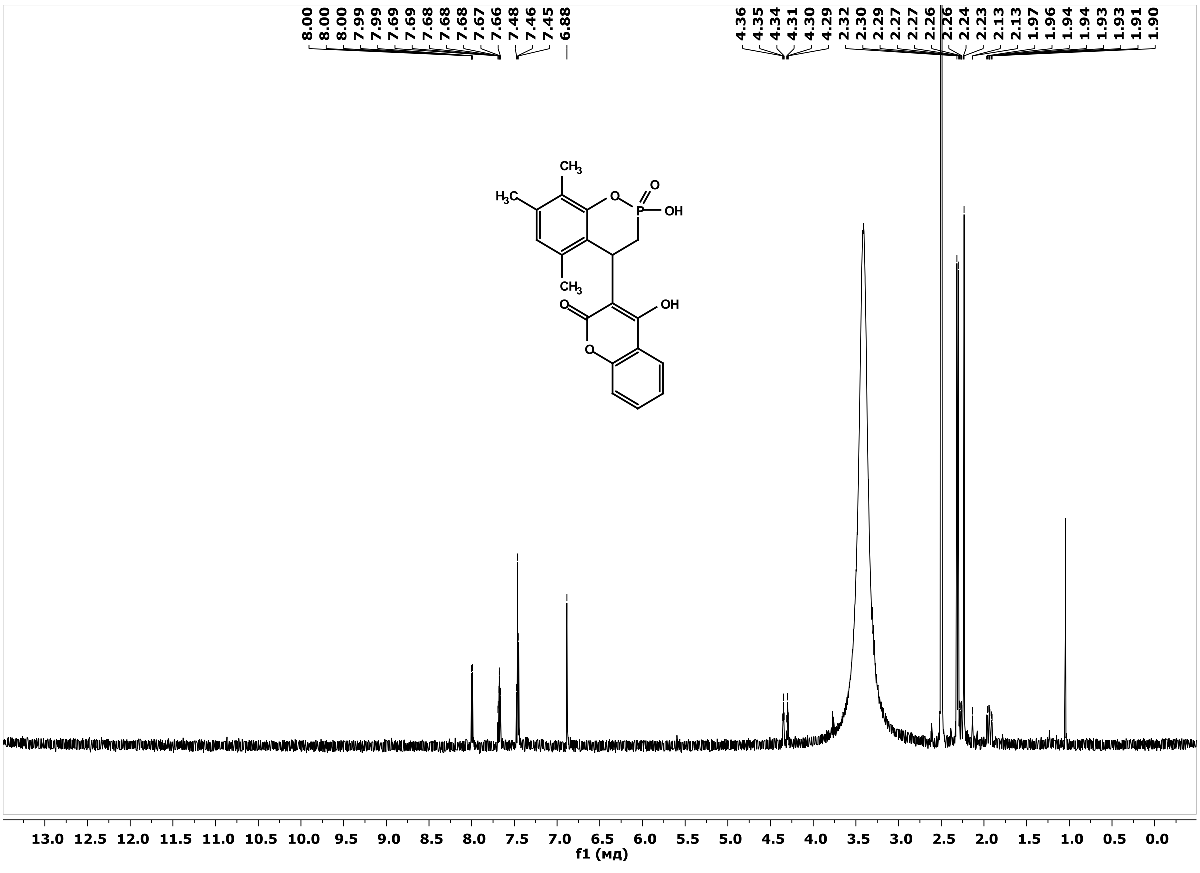

Figure S38. ^{1}H NMR (DMSO-d_6, 600 MHz) spectrum of the compound **2k**

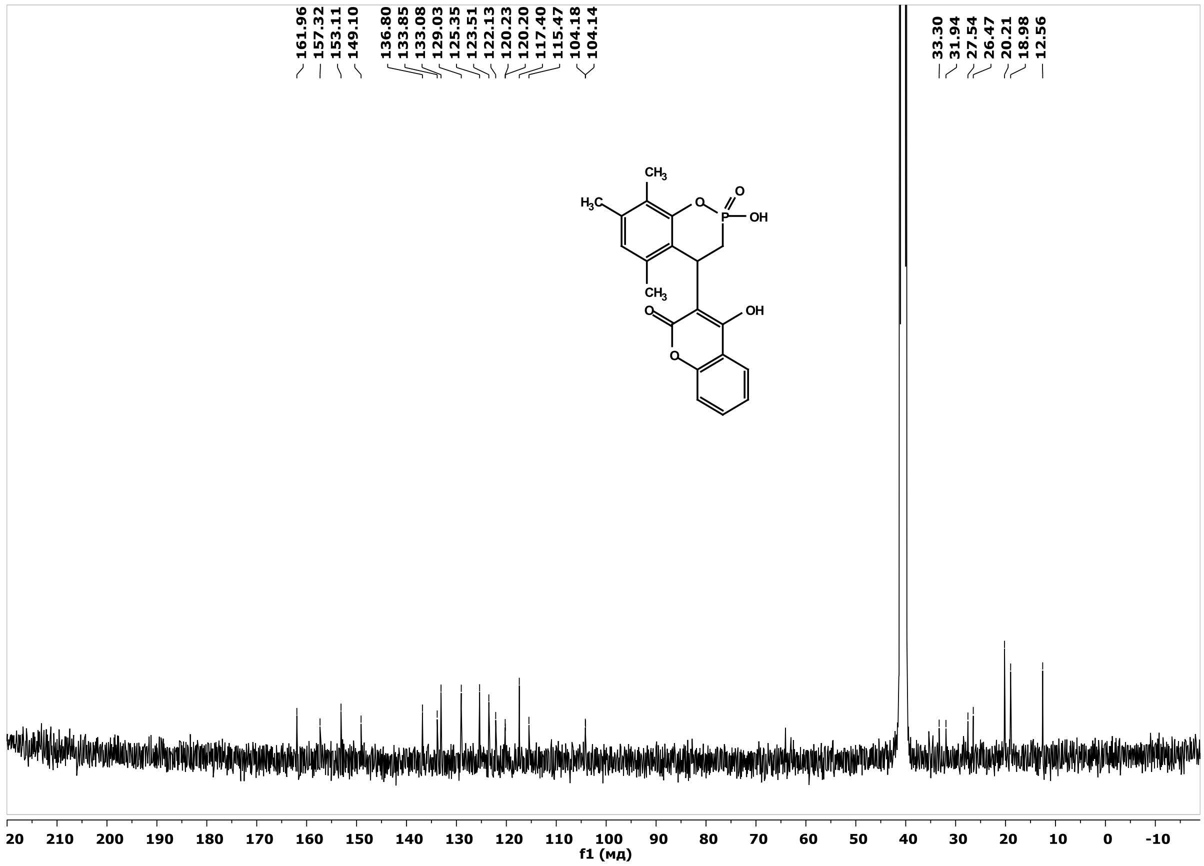

Figure S39. $^{13}C\{^{1}H\}$ NMR (DMSO-d_6, 101 MHz) spectrum of the compound **2k**

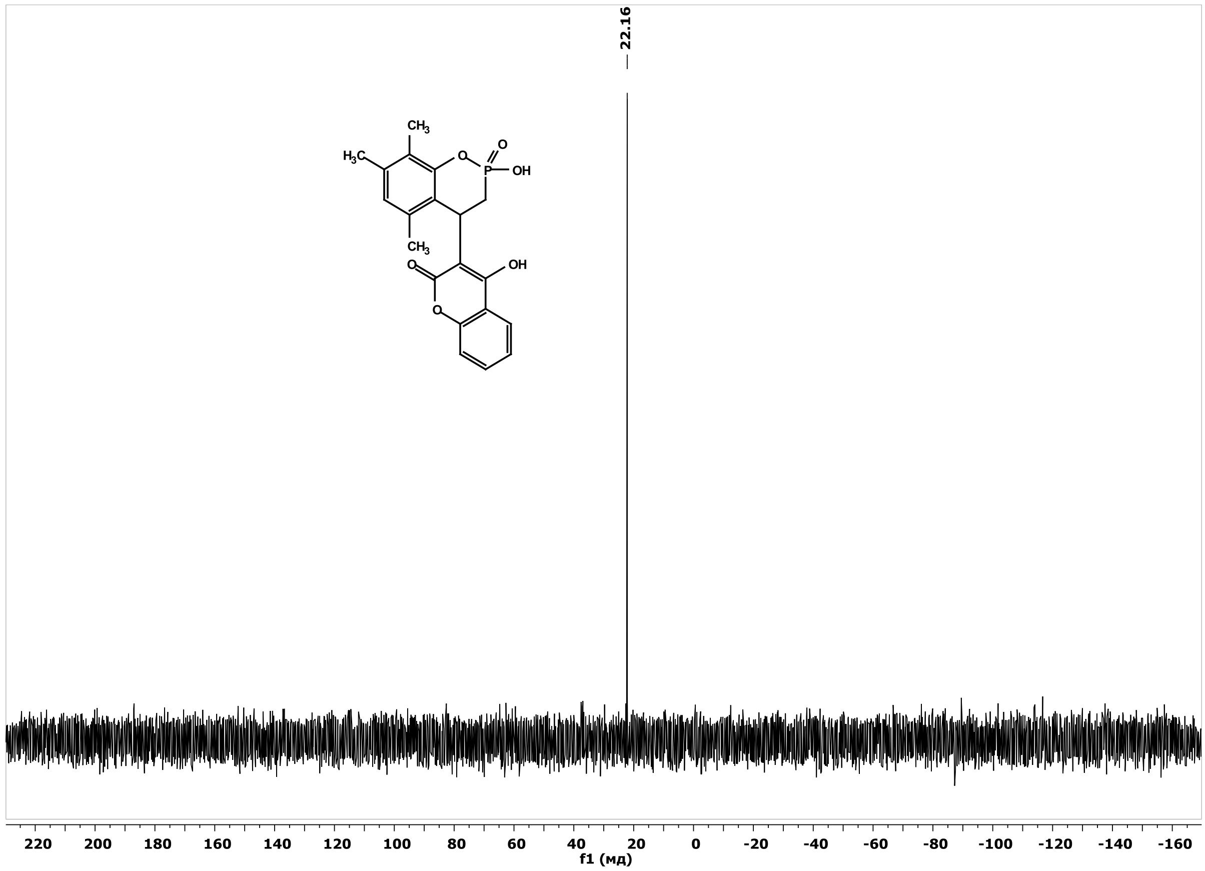

Figure S40. ^{31}P NMR (DMSO-d_6, 243 MHz) spectrum of the compound **2k**

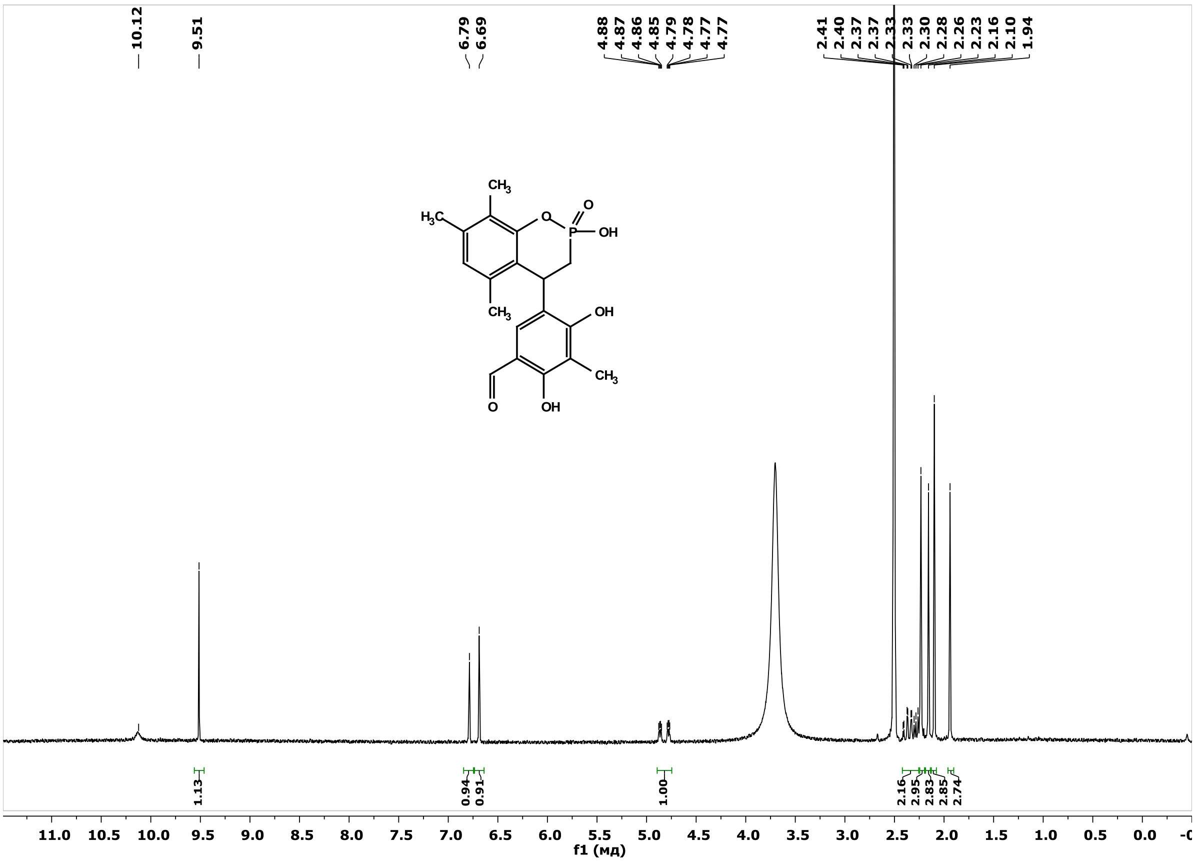

Figure S41. ^{1}H NMR (DMSO-d_6, 400 MHz) spectrum of the compound **2l**

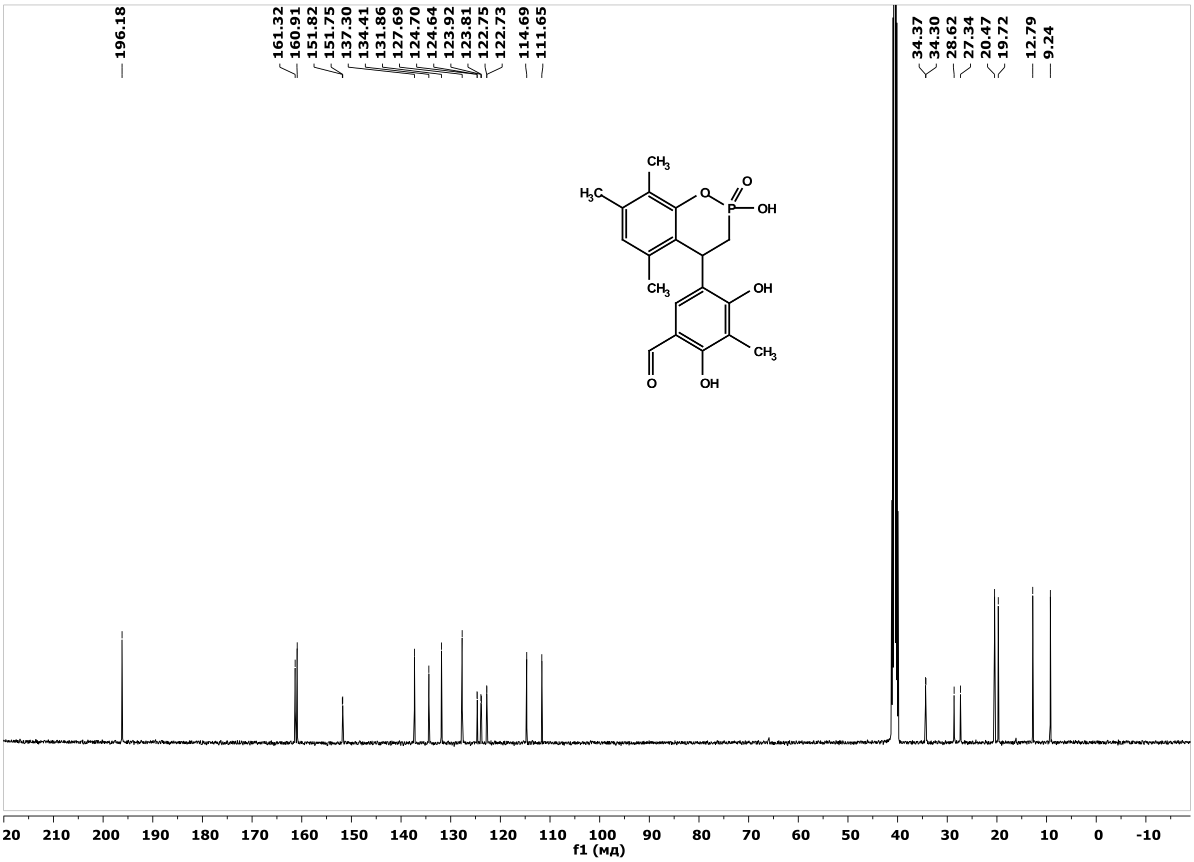

Figure S42. $^{13}C\{^{1}H\}$ NMR (DMSO-d_6, 101 MHz) spectrum of the compound **2l**

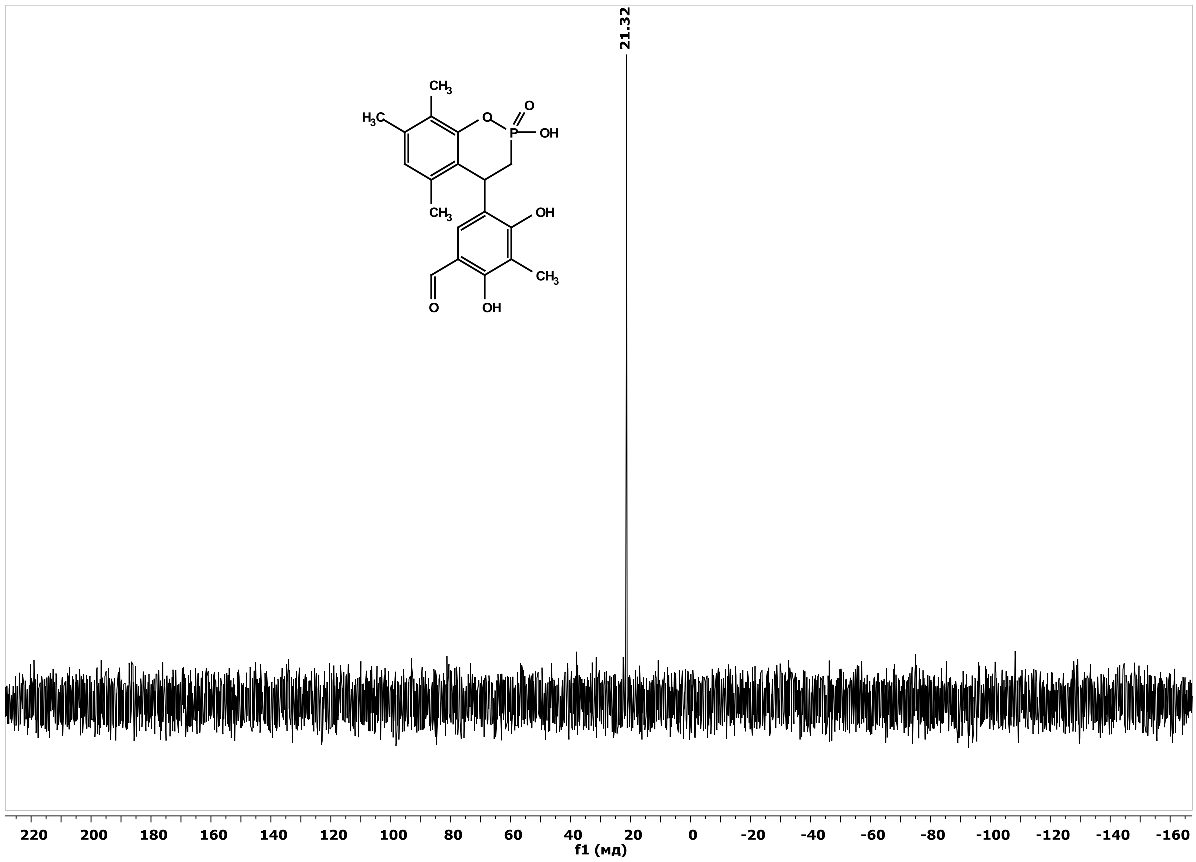

Figure S43. ^{31}P NMR (DMSO-d_6, 162 MHz) spectrum of the compound **2l**

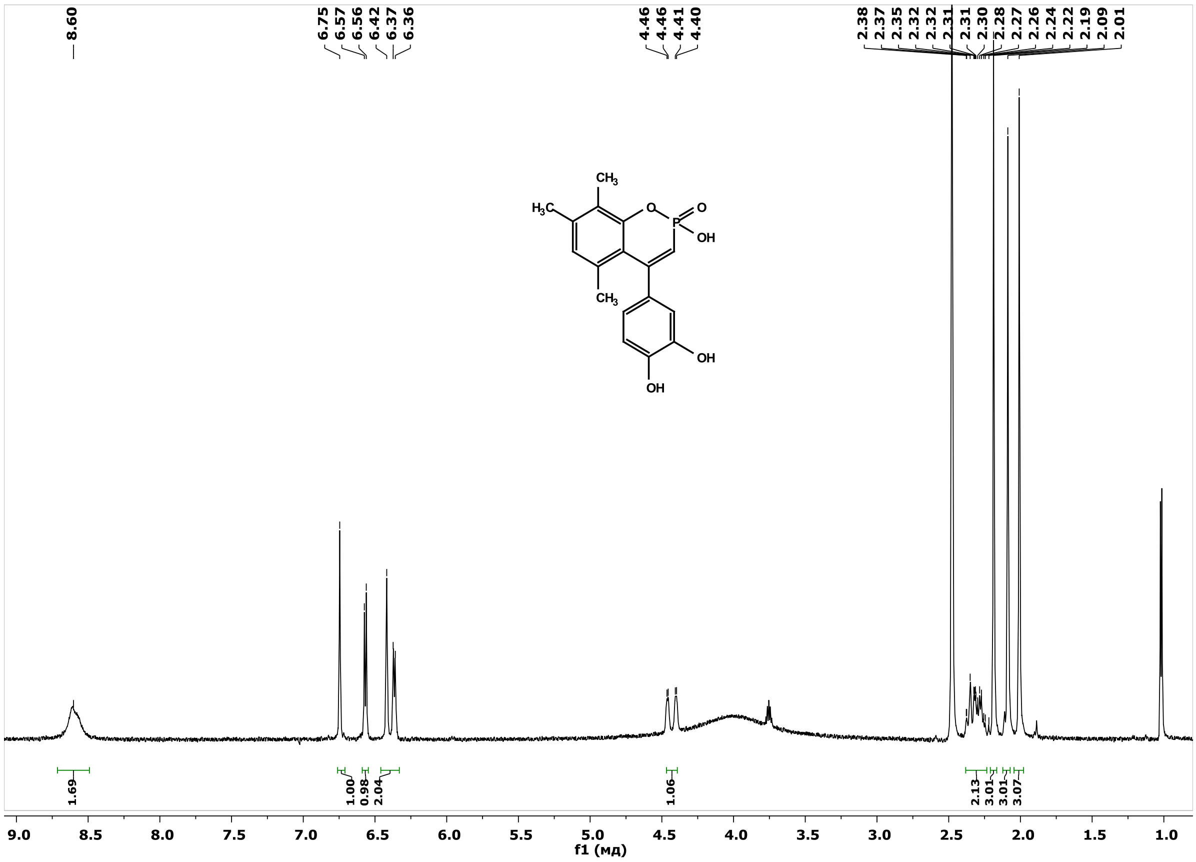

Figure S44. ^{1}H NMR (DMSO-d_6, 600 MHz) spectrum of the compound **2m**

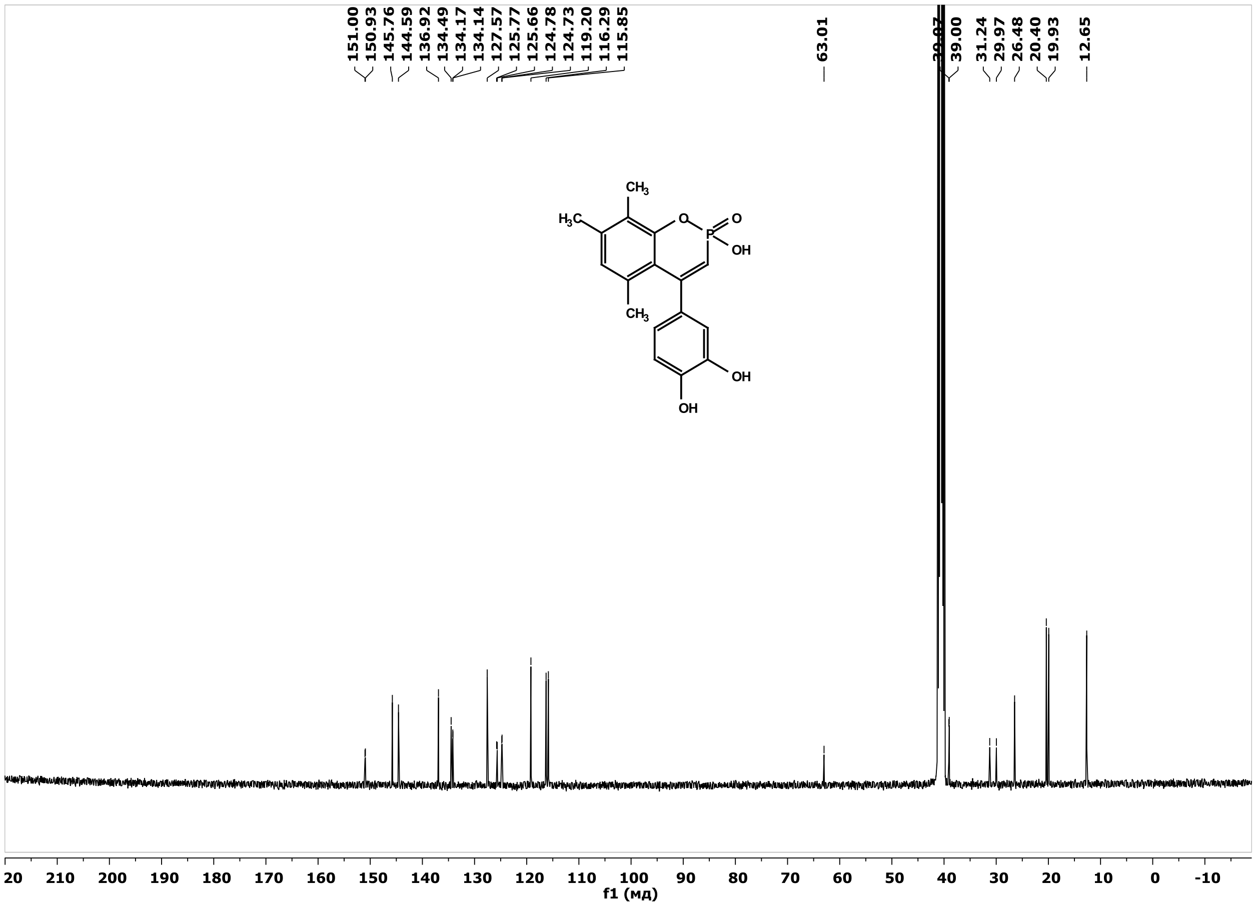

Figure S45. $^{13}C\{^{1}H\}$ NMR (DMSO-d_6, 101 MHz) spectrum of the compound **2m**

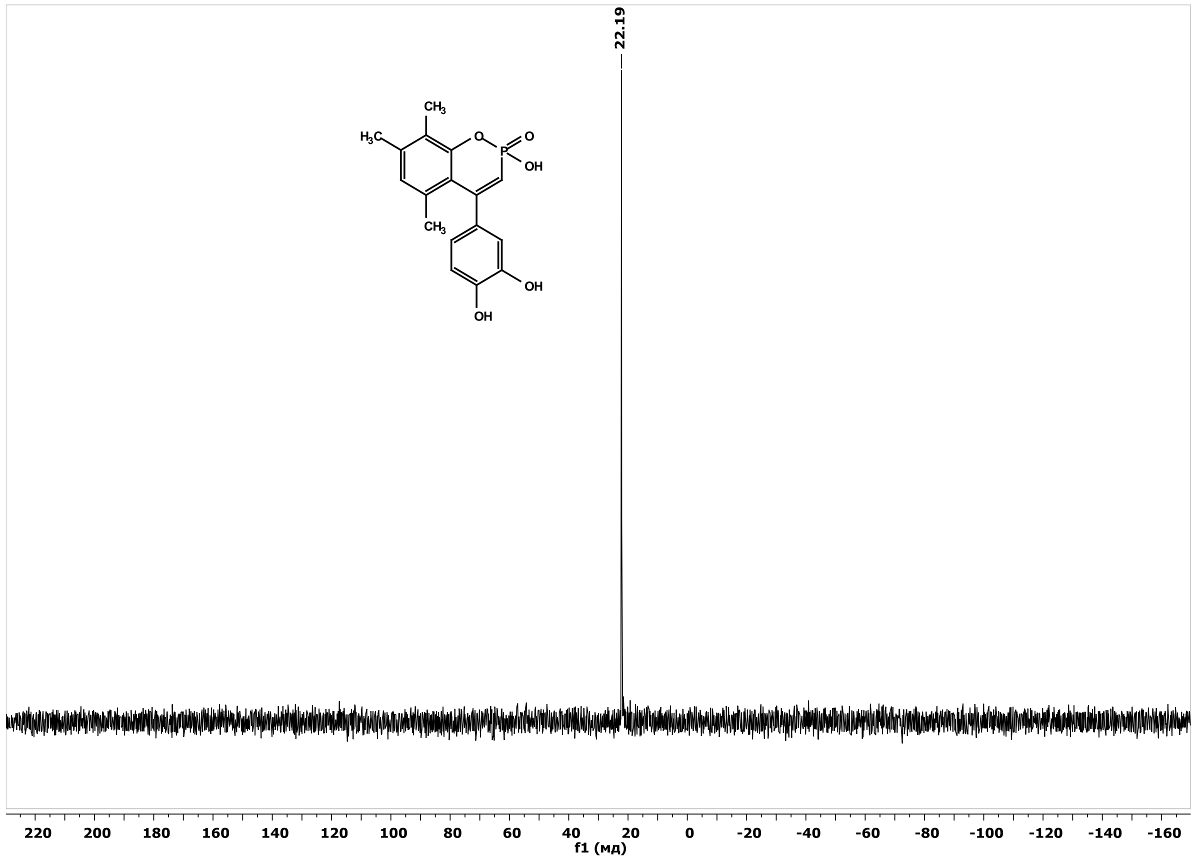

Figure S46. ^{31}P NMR (DMSO-d_6, 243 MHz) spectrum of the compound **2m**

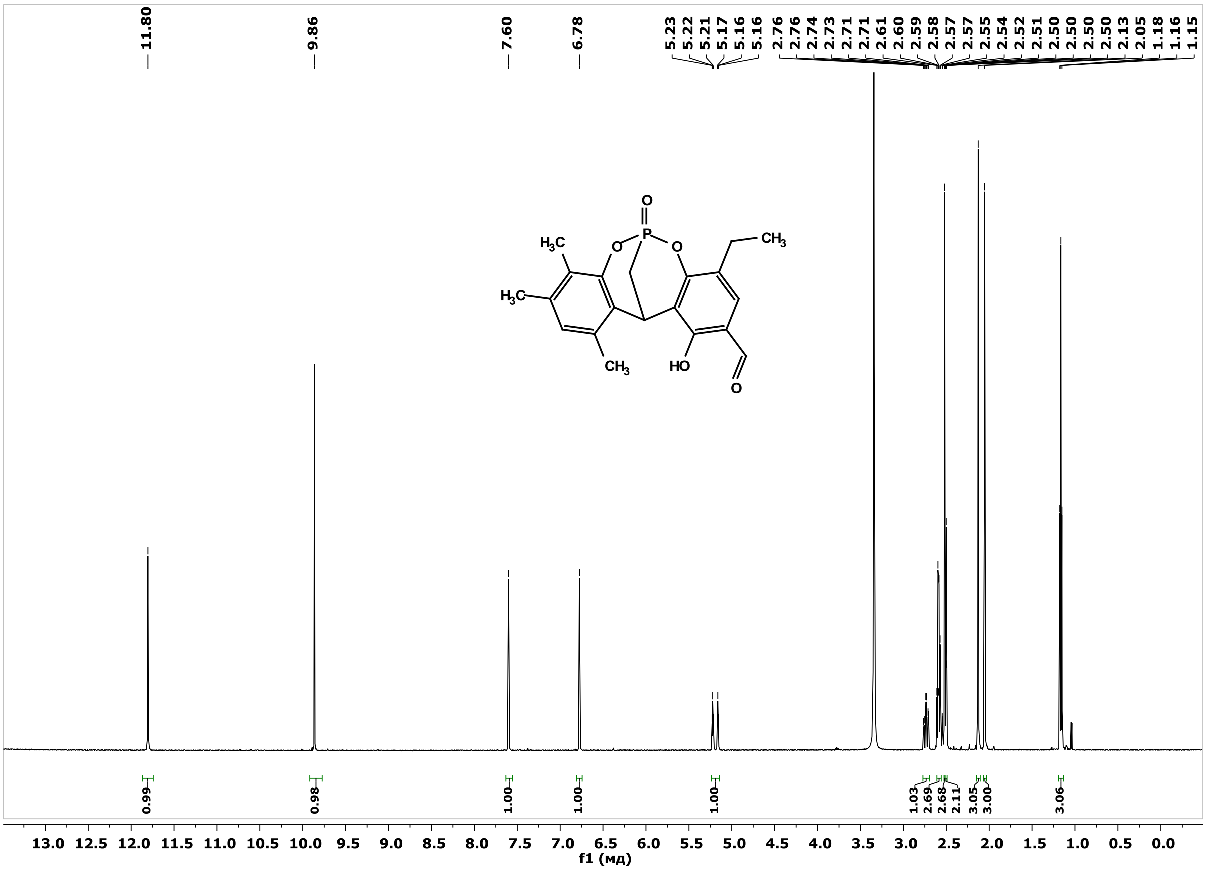

Figure S47. ^{1}H NMR (DMSO-d_6, 600 MHz) spectrum of the compound **3**

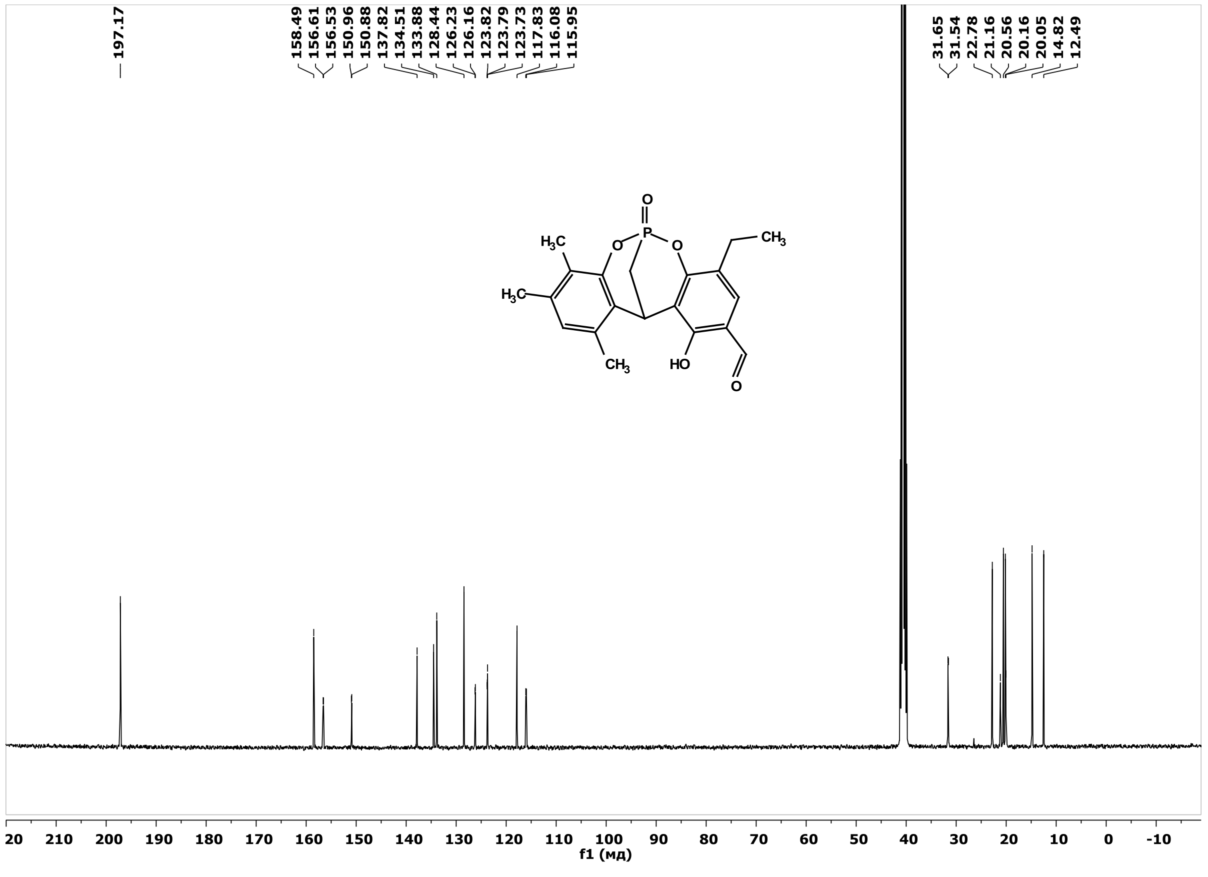

Figure S48. $^{13}C\{^{1}H\}$ NMR (DMSO-d_6, 101 MHz) spectrum of the compound **3**

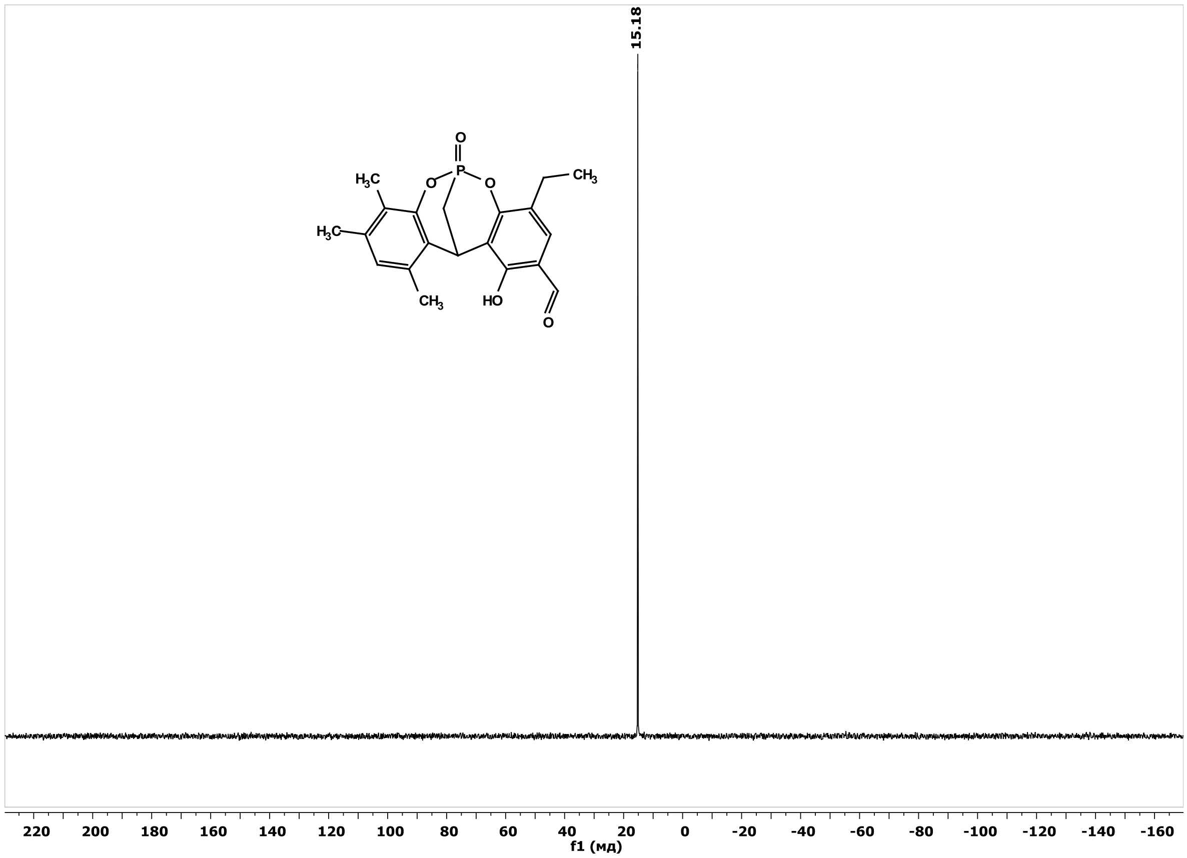

Figure S49. ^{31}P NMR (DMSO-d_6, 243 MHz) spectrum of the compound **3**

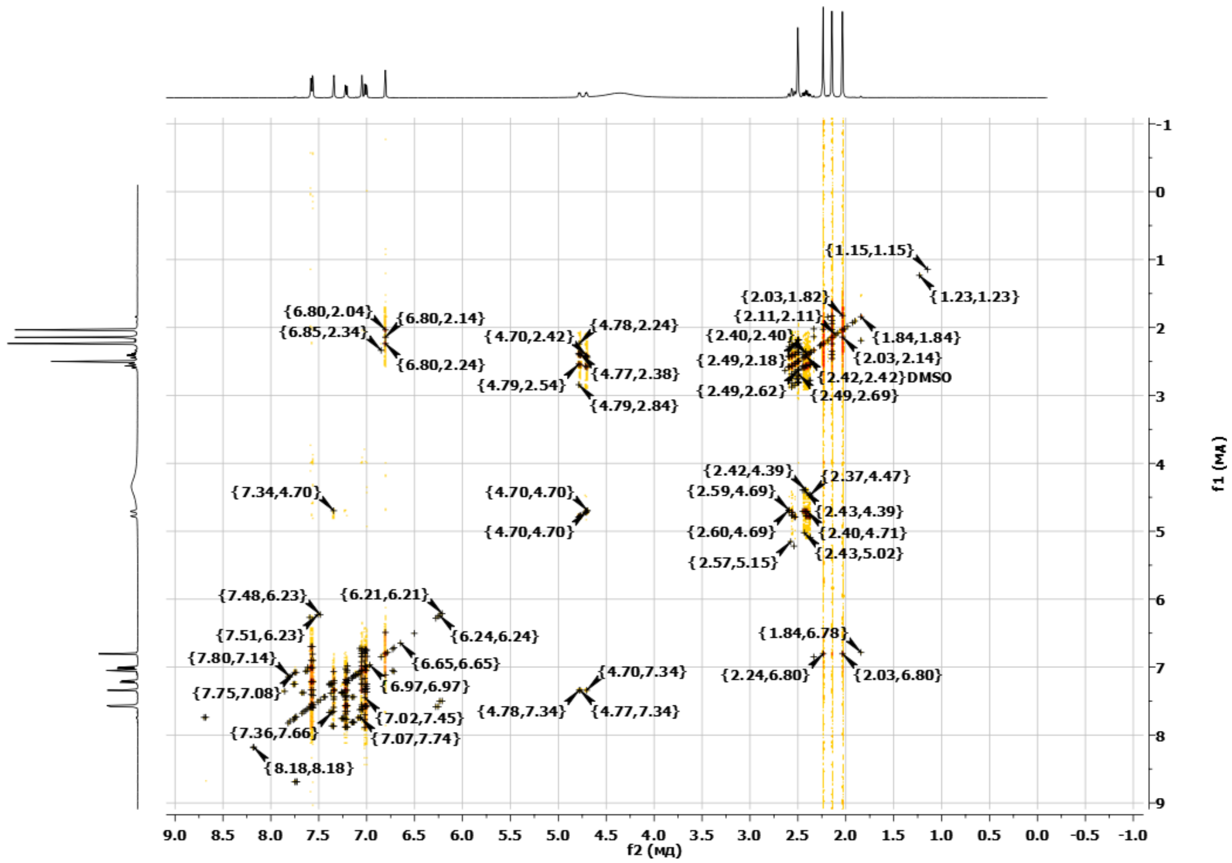

Figure S50. ^{1}H-^{1}H COSY spectrum (DMSO-d_6) of the compound **2i**

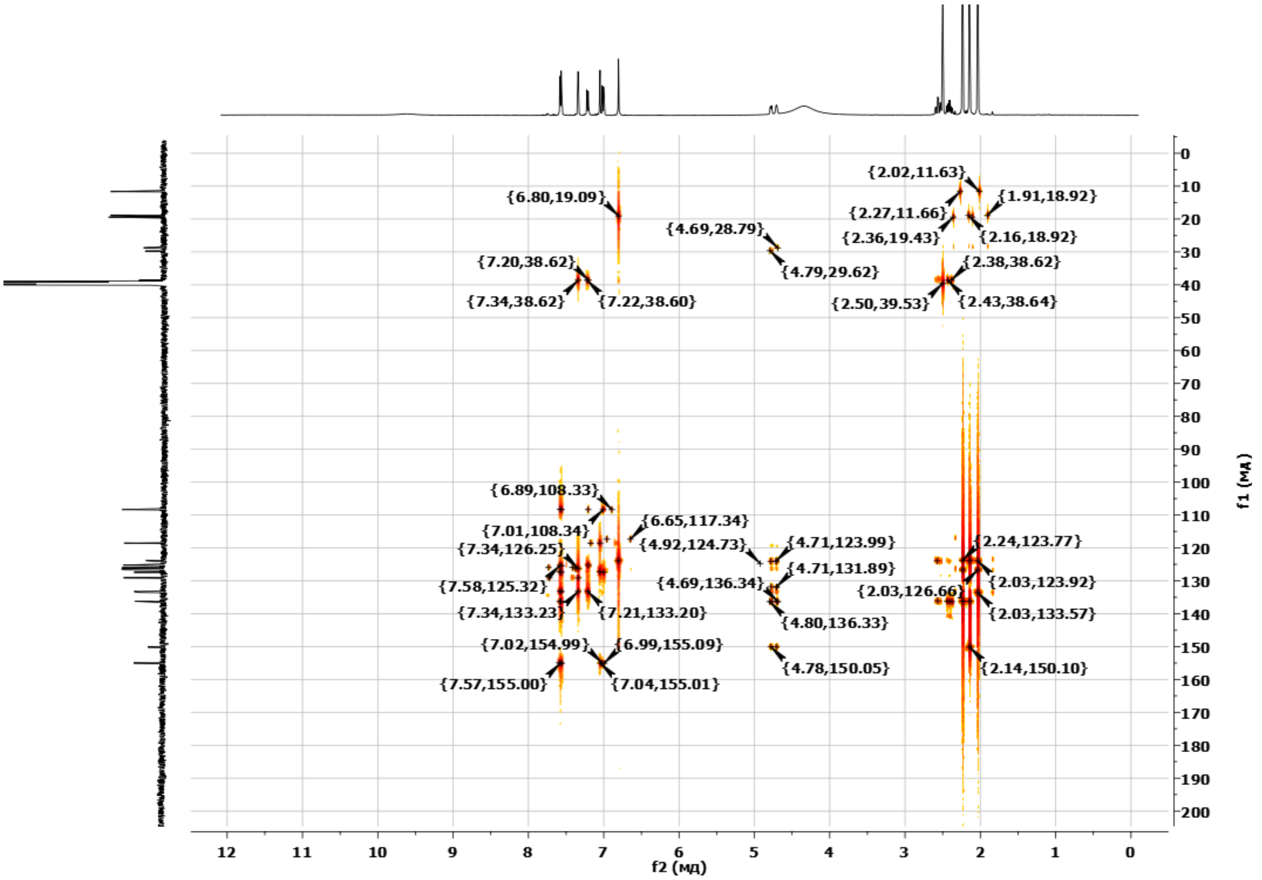

Figure S51. ^{1}H-^{13}C HMBC spectrum (DMSO-d_6) of the compound **2i**

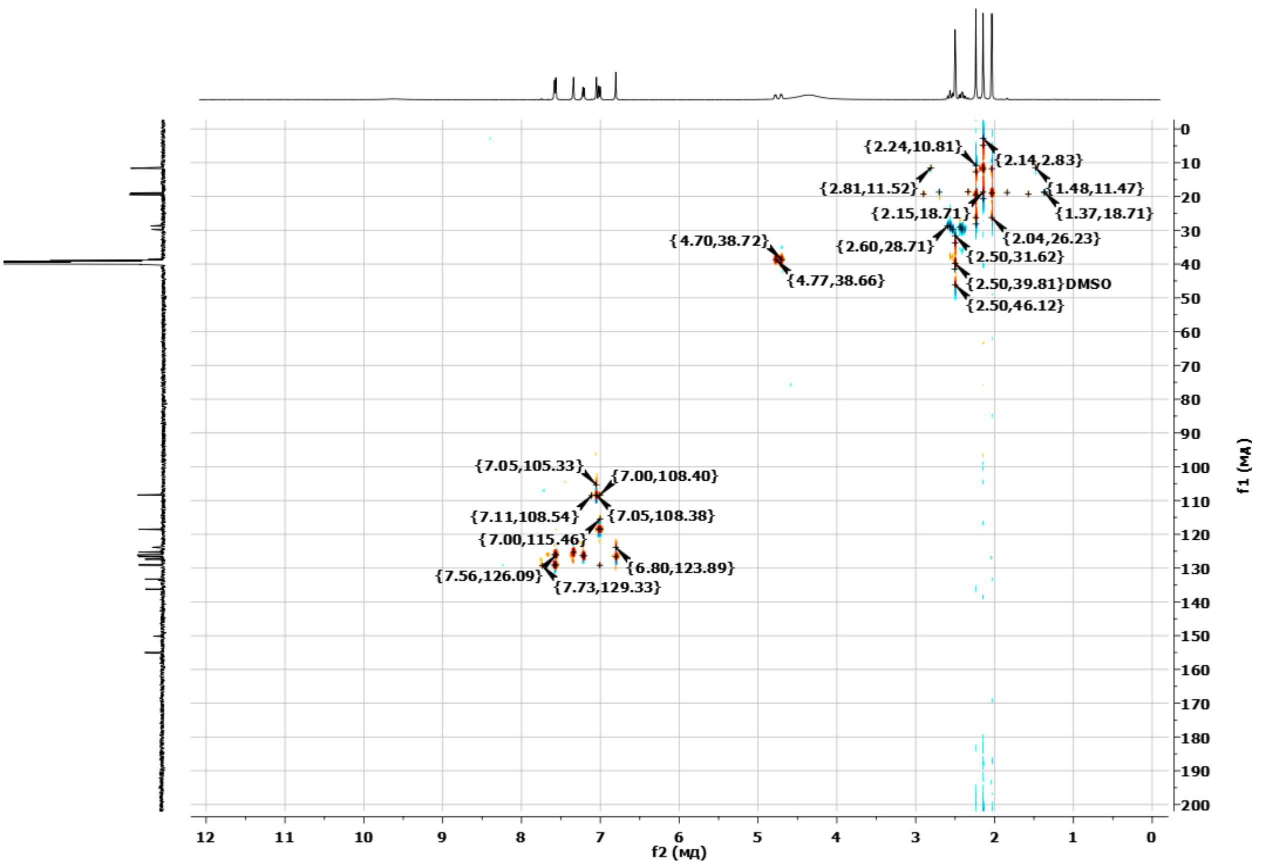

Figure S52. ^{1}H-^{13}C HSQC spectrum (DMSO-d_6) of the compound **2i**

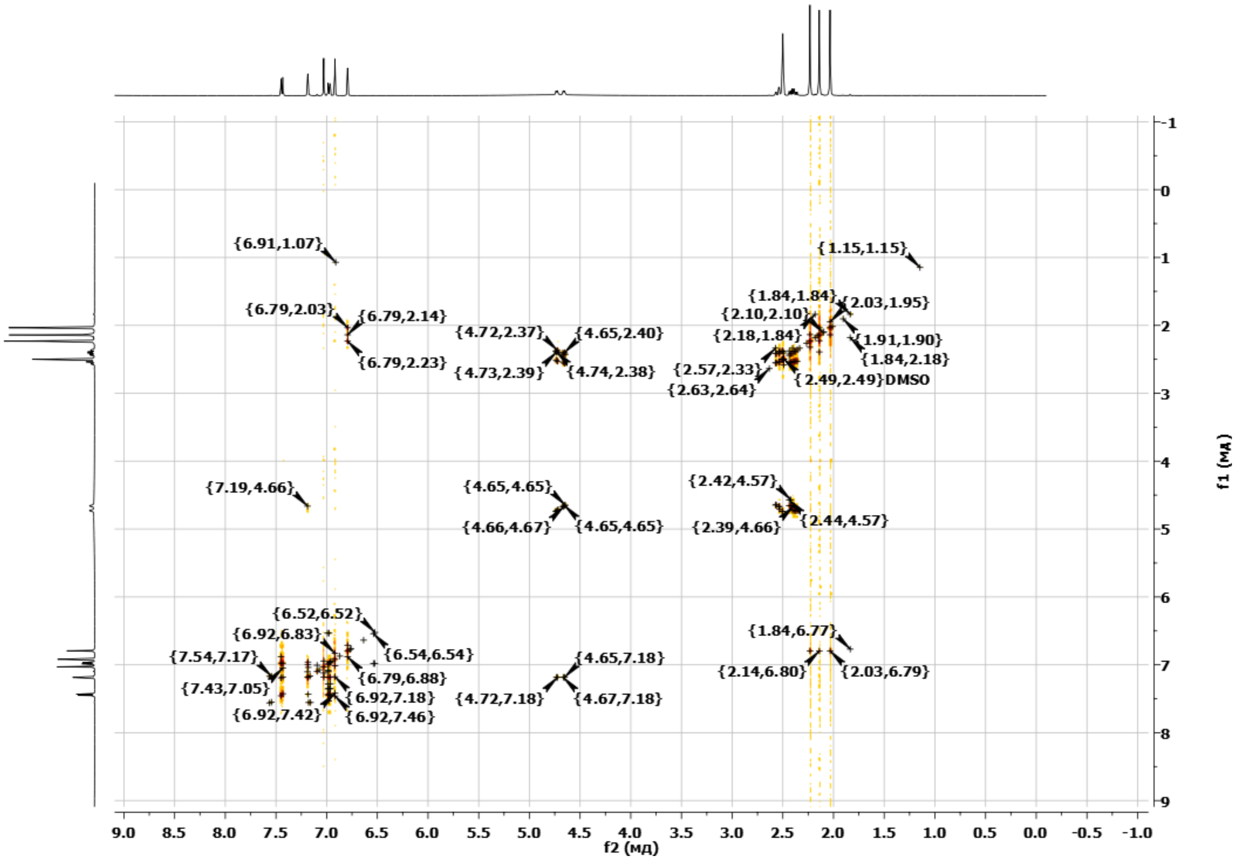

Figure S53. ^1H-^1H COSY spectrum (DMSO-d_6) of the compound **2j**

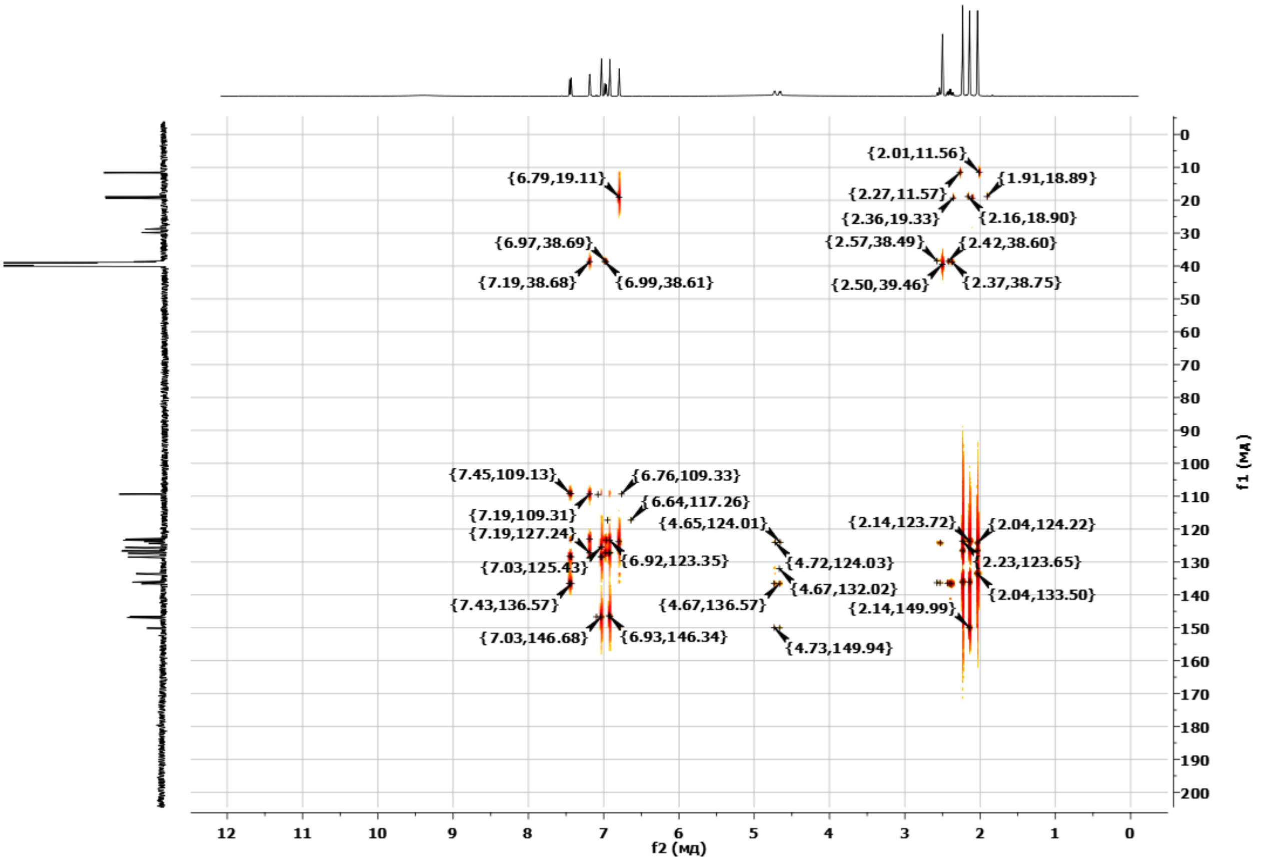

Figure S54. ^1H-^{13}C HMBC spectrum (DMSO-d_6) of the compound **2j**

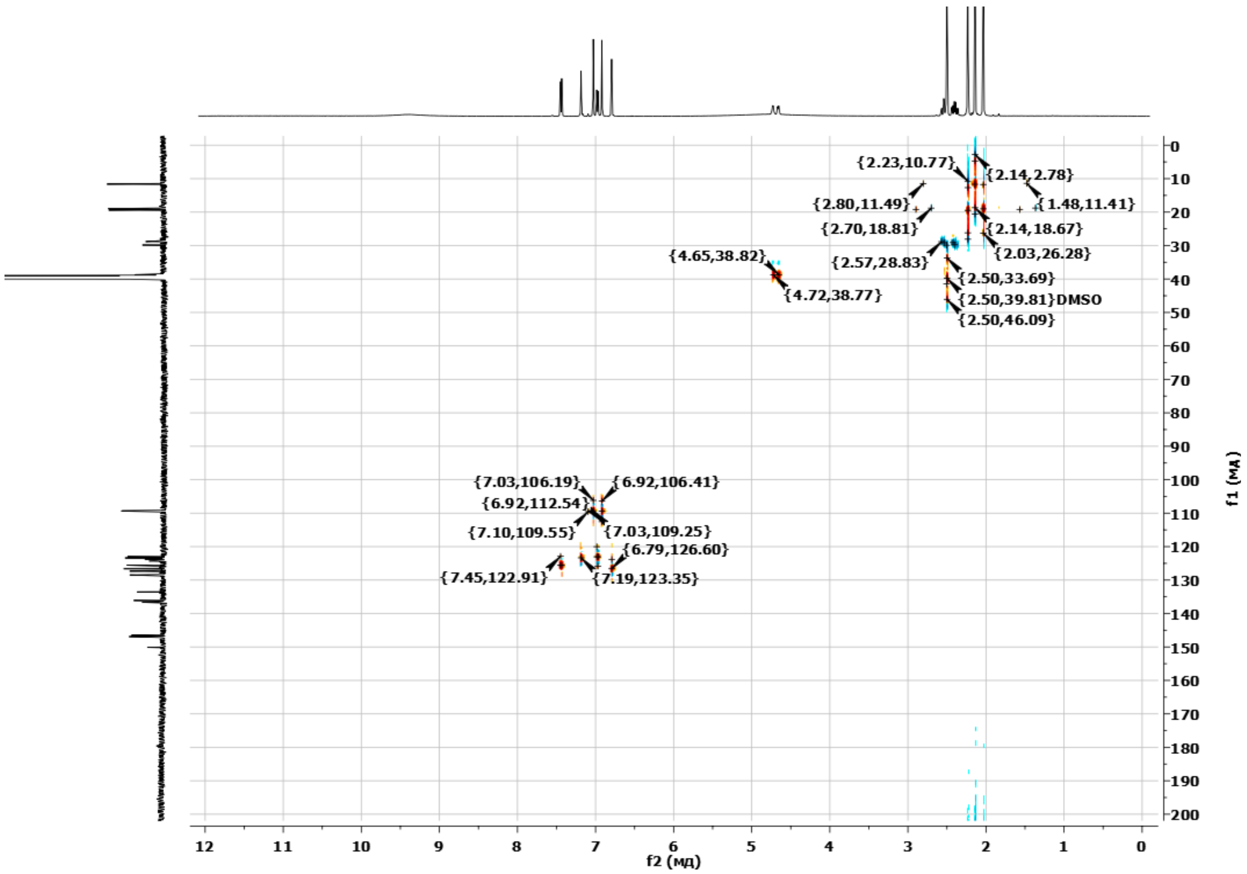

Figure S55. ^{1}H-^{13}C HSQC spectrum (DMSO-d_6) of the compound **2j**

Coordinates of stationary points

Molecule: **Cat**

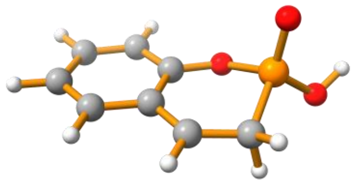

Energy = -876.89878141 Hartree

20

C	-4.09571	-1.00966	0.17423
C	-2.73844	-1.03517	0.23839
C	-1.97921	0.16010	0.02302
C	-2.67957	1.38906	-0.24286
C	-4.06123	1.39325	-0.30922
C	-4.74594	0.20986	-0.10399
H	-0.13787	-0.84974	0.22244
H	-4.67762	-1.90588	0.33139
H	-2.20290	-1.95261	0.44614
C	-0.61257	0.10779	0.03477
H	-4.59040	2.31359	-0.51427
H	-5.82706	0.22565	-0.15419
C	0.27887	1.24750	-0.23916
H	0.53050	1.20177	-1.31055
O	-2.02931	2.52268	-0.45595
P	-0.50220	2.82650	0.13681
O	-0.53144	3.24237	1.54355
O	0.04048	3.83286	-0.95088
H	0.10033	4.75535	-0.65783
H	1.22528	1.13973	0.29414

Molecule: **Cat_PHOS**

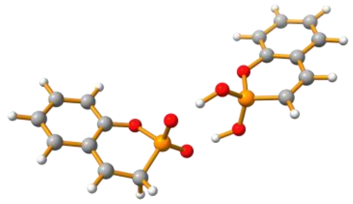

Energy = -1753.42357372 Hartree

39

C	-1.74308	11.18611	1.22123
C	-0.39115	10.90420	1.18312
C	0.07132	9.64498	0.78933
C	-0.87189	8.67811	0.43056
C	-2.22971	8.94971	0.45731
C	-2.65968	10.20537	0.85480
H	2.15689	10.19180	0.93785
H	-2.08544	12.16381	1.53081
H	0.33202	11.66123	1.46057
C	1.49074	9.35990	0.73658
H	-2.93767	8.18393	0.16850
H	-3.72008	10.41825	0.87961
C	2.02639	8.16097	0.46272
H	3.09857	8.01799	0.43987
O	-0.48831	7.44197	0.00795
P	0.98242	6.78368	0.17017
O	1.27953	5.97723	-1.06726
O	0.95484	5.93320	1.49529
H	0.64724	4.98653	1.38696
C	-4.17163	-0.19794	0.84972
C	-2.84285	-0.42777	0.68258
C	-1.98505	0.60298	0.17982
C	-2.55070	1.89131	-0.12305
C	-3.90786	2.10295	0.04813
C	-4.69383	1.07034	0.52335
H	-0.29589	-0.66215	0.17618
H	-4.82743	-0.97044	1.22323
H	-2.40613	-1.39013	0.91648
C	-0.66390	0.33384	-0.04920
H	-4.33750	3.06678	-0.18745
H	-5.75331	1.24813	0.65456
C	0.30167	1.28026	-0.62970
H	0.33856	1.07207	-1.71067
O	-1.81179	2.88188	-0.59410
P	-0.16544	3.00188	-0.37267
O	0.12862	3.50329	0.99711
O	0.27077	3.83344	-1.59411
H	0.71325	4.78754	-1.38682
H	1.30948	1.08576	-0.25736

Molecule: **Cat_TFA**

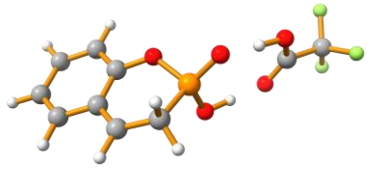

Energy = -1403.99945546 Hartree

28

C	-4.37583	-0.31159	-0.55038
C	-3.09691	-0.76809	-0.61385
C	-2.01371	0.03990	-0.13859
C	-2.30184	1.34837	0.38389
C	-3.60791	1.79141	0.44983
C	-4.61952	0.96636	-0.01030
H	-0.58623	-1.46565	-0.52582
H	-5.20026	-0.91359	-0.90285
H	-2.86768	-1.74665	-1.01619
C	-0.74250	-0.46127	-0.14590
H	-3.82911	2.76991	0.85331
H	-5.63938	1.32478	0.04427
C	0.44804	0.23165	0.37514
H	1.33757	-0.04210	-0.19741
O	-1.34279	2.14663	0.83772
P	0.26164	2.01607	0.46562
O	1.04240	2.72973	1.50590
O	0.38085	2.52644	-1.00724
H	0.77486	3.44406	-1.08960
C	2.73064	6.80073	-0.26317
C	2.03951	5.44233	-0.06684
F	2.55696	7.24091	-1.49857
F	4.03625	6.68030	-0.03501
F	2.23891	7.69964	0.58494
O	2.17797	5.01718	1.14041
H	1.74175	4.12152	1.30646
O	1.46887	4.91345	-0.99519
H	0.62545	-0.15443	1.39144

Molecule: **diCat**

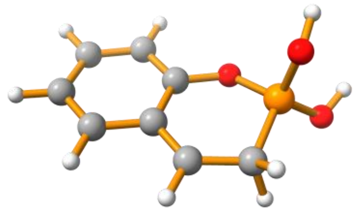

Energy = -877.27024910 Hartree

21

C	-4.09914	-0.95748	0.17527
C	-2.73826	-1.03498	0.17489
C	-1.94513	0.14350	-0.00746
C	-2.62038	1.39881	-0.17580
C	-3.98628	1.46823	-0.18655
C	-4.71143	0.29062	-0.01107
H	-0.13890	-0.95120	0.03980
H	-4.70531	-1.84077	0.31202
H	-2.23050	-1.98155	0.30835
C	-0.58430	0.03352	-0.05472
H	-4.49096	2.41405	-0.32825
H	-5.79219	0.35038	-0.01810
C	0.37433	1.13809	-0.26805
H	0.75545	1.05763	-1.29866
O	-1.92378	2.54211	-0.36841
P	-0.36886	2.73849	-0.02750
O	-0.19222	3.10414	1.46917
O	0.18646	3.75263	-1.05489
H	0.03482	4.70927	-0.94331
H	1.25147	1.01769	0.37633
H	-0.49891	3.95883	1.82294

Molecule: **diCat_PHOS**

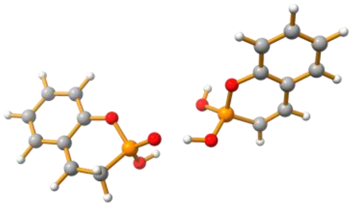

Energy = -1753.80511839 Hartree

40

C	-0.68451	11.81367	1.98934
C	0.31464	11.42921	1.11741
C	0.31329	10.15326	0.54412
C	-0.72908	9.28621	0.87656
C	-1.73988	9.65439	1.74301
C	-1.70924	10.92437	2.29882
H	2.10465	10.50595	-0.60370
H	-0.67082	12.80093	2.42940
H	1.11613	12.11317	0.86777
C	1.36041	9.75385	-0.36630
H	-2.53534	8.95999	1.97841
H	-2.49448	11.22003	2.98145
C	1.48747	8.53710	-0.92686
H	2.30210	8.30235	-1.59865
O	-0.79740	8.02879	0.32785
P	0.32986	7.30429	-0.53496
O	-0.40492	6.62739	-1.75246
O	0.99563	6.14841	0.23836
H	0.46892	5.29978	0.46885
C	-3.91250	-1.16098	-0.44377
C	-2.70099	-1.12760	0.17441
C	-2.01463	0.11530	0.36020
C	-2.62120	1.31986	-0.13775
C	-3.85147	1.27058	-0.75591
C	-4.47989	0.04295	-0.89979
H	-0.44249	-0.80117	1.43030
H	-4.44025	-2.09276	-0.58343
H	-2.23470	-2.03125	0.54534
C	-0.83369	0.13633	1.04842
H	-4.31417	2.17719	-1.12088
H	-5.44559	0.01495	-1.38766
C	-0.05859	1.34675	1.37512
H	1.01328	1.13413	1.38529
O	-2.03613	2.50876	0.01142
P	-0.43778	2.73502	0.30775
O	-0.27020	4.09791	0.86602
O	0.30411	2.36816	-1.03138
H	0.31014	3.03651	-1.73692
H	-0.86338	7.22173	-2.36824
H	-0.31231	1.62250	2.41102

Molecule: **diCat_TFA**

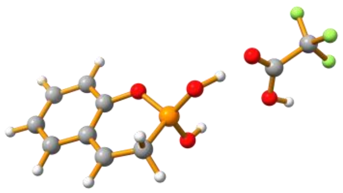

Energy = -1404.36502238 Hartree

29

C	-4.47915	-0.48950	-0.15629
C	-3.20742	-0.97835	-0.14811
C	-2.09301	-0.09625	0.02443
C	-2.34980	1.30749	0.18488
C	-3.63243	1.78959	0.18132
C	-4.68098	0.88947	0.01024
H	-0.70701	-1.68836	-0.02568
H	-5.32599	-1.14710	-0.28607
H	-3.01217	-2.03607	-0.26911
C	-0.82929	-0.61414	0.06454
H	-3.82376	2.84582	0.31084
H	-5.69189	1.27625	0.00476
C	0.41529	0.15249	0.27011
H	1.22514	-0.24702	-0.34769
O	-1.34167	2.18066	0.38138
P	0.20093	1.90187	-0.00209
O	1.06001	2.73581	0.93787
O	0.38921	2.12658	-1.53178
H	0.30835	3.02395	-1.90046
C	2.96847	7.06691	0.25238
C	2.32881	5.70313	-0.08800
F	3.58661	7.59464	-0.79786
F	3.84420	6.89811	1.22764
F	2.02770	7.90466	0.65781
O	1.84409	5.07299	0.82443
H	1.35348	3.72842	0.79069
O	2.32537	5.27853	-1.30782
H	2.75010	5.86685	-1.95393
H	0.73381	-0.01544	1.31156

43

Molecule: **IM1**

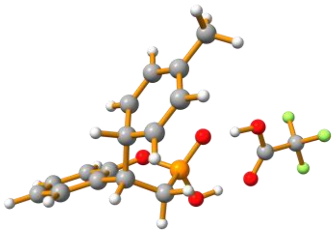

Energy = -1675.70180409 Hartree

C	-4.55000	-0.85400	-1.24000
C	-3.27700	-1.21800	-0.80600
C	-2.33200	-0.25000	-0.44000
C	-2.70900	1.10000	-0.52700
C	-3.97500	1.48400	-0.96400
C	-4.89600	0.50000	-1.32000
H	-0.72200	-1.64000	-0.37300
H	-5.27000	-1.62200	-1.52200
H	-2.99500	-2.27300	-0.74900
C	-0.95000	-0.63700	0.01300
H	-4.22200	2.54500	-1.01400
H	-5.89000	0.79300	-1.66200
C	0.10500	0.32400	-0.56600
H	0.09200	0.23000	-1.66300
O	-1.85100	2.11900	-0.10700
P	-0.23000	2.04700	-0.18200
O	0.32700	2.53700	1.13100
O	0.20100	2.90400	-1.42800
H	0.72400	3.75200	-1.17800
C	2.77000	6.55900	0.62900
C	1.93700	5.27300	0.34400
F	3.06600	7.20800	-0.50900
F	3.92700	6.25000	1.25800
F	2.06900	7.39800	1.42800
O	1.71500	4.60100	1.43200
H	1.12500	3.73000	1.26600
O	1.57400	5.02400	-0.80500
H	1.12200	0.08900	-0.22100
C	-0.84600	-0.77800	1.58900
C	0.44900	-1.28100	2.05600
C	-1.47900	0.25400	2.41500
C	0.99500	-0.85400	3.24100
H	0.95300	-2.03400	1.44500
C	-0.92600	0.64500	3.61300
H	-2.43600	0.66100	2.08600
C	0.30800	0.10500	4.01400
H	1.95000	-1.24700	3.58800
H	-1.42500	1.38500	4.23700
H	-1.52900	-1.65400	1.77900
C	0.93300	0.57900	5.33900
H	0.85900	-0.20200	6.06800
H	1.96200	0.82300	5.18100
H	0.41100	1.44400	5.69100

Molecule: **IM2**

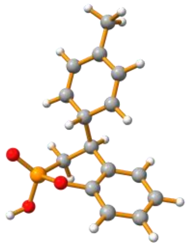

Energy = -1148.59801062 Hartree

35

C	-3.90600	-1.15200	-0.84300
C	-2.52000	-1.14400	-0.67700
C	-1.87100	-0.02200	-0.15000
C	-2.65000	1.09200	0.20300
C	-4.03400	1.10100	0.04700
C	-4.65900	-0.03100	-0.47800
H	-4.39900	-2.03200	-1.25700
H	-1.92500	-2.01600	-0.95900
C	-0.38000	0.04300	0.03300
H	-4.60100	1.98600	0.33600
H	-5.74300	-0.03400	-0.60500
C	0.14900	1.35800	-0.56600
H	-0.11400	1.37700	-1.63400
O	-2.03800	2.19900	0.79000
P	-0.60900	2.79000	0.23700
O	0.11500	3.41700	1.37200
O	-0.97300	3.74900	-0.99400
H	-1.16100	4.66900	-0.71600
H	1.23900	1.46400	-0.49500
H	0.09100	-0.79900	-0.49100
C	0.01800	-0.13300	1.57400
C	-0.64600	-1.26300	2.22700
C	1.44000	0.04200	1.88100
C	0.02000	-2.07300	3.11500
H	-1.69900	-1.43700	1.99900
C	2.09200	-0.79400	2.75500
H	1.97100	0.88200	1.43100
C	1.38800	-1.84900	3.36300
H	-0.49400	-2.89800	3.60800
H	3.14800	-0.64400	2.98100
H	-0.46200	0.76600	2.06100
C	2.13500	-2.78600	4.33000
H	1.47100	-3.09900	5.10800
H	2.48600	-3.64400	3.79500
H	2.96700	-2.26800	4.75800

54

C	-2.26780	6.65776	3.25828
C	-0.88682	6.76842	3.37022
C	-0.04295	6.46692	2.28071
C	-0.65358	6.05412	1.07976
C	-2.03261	5.93897	0.94934
C	-2.83866	6.24329	2.04828
H	1.77037	6.96313	3.35257
H	-2.90504	6.89388	4.11070
H	-0.42860	7.09271	4.30658
C	1.39220	6.58804	2.39708
H	-2.45877	5.61444	-0.00067
H	-3.92160	6.15268	1.95528
C	2.28876	6.27372	1.42627
H	3.36409	6.38463	1.57035
O	0.10919	5.75367	-0.04550
P	1.71232	5.63807	-0.09192
O	2.16424	6.44393	-1.37540
O	2.15974	4.19304	-0.32514
H	1.70072	3.55133	-1.11348
C	-4.88996	1.38673	-0.21449
C	-3.68645	0.99110	0.37208
C	-2.46510	1.48005	-0.10563
C	-2.49562	2.36179	-1.19433
C	-3.67976	2.77223	-1.79385
C	-4.88629	2.27984	-1.29054
H	-1.27406	0.25984	1.18857
H	-5.83355	0.99757	0.16935
H	-3.68582	0.29823	1.21636
C	-1.13704	1.11201	0.50929
H	-3.64818	3.46223	-2.63755
H	-5.82575	2.59197	-1.74830
C	-0.15325	0.67596	-0.59726
H	-0.53451	-0.25193	-1.04756
O	-1.28129	2.89755	-1.67366
P	-0.03431	1.89366	-1.92492
O	1.21581	2.73137	-2.02859
O	-0.30927	1.05150	-3.24013
H	-0.13297	1.53206	-4.07670
H	0.85930	0.46190	-0.23000
C	-1.24375	3.26336	3.59191
C	0.09603	3.15707	4.04559
C	1.08447	2.59639	3.19202
C	0.76248	2.16792	1.93590
C	-0.59713	2.31004	1.39395
C	-1.59034	2.84523	2.33737
H	-1.99734	3.67662	4.26281
H	2.10869	2.50837	3.55770
H	1.54223	1.76115	1.29176
H	-0.48295	3.14506	0.65092
H	-2.62429	2.92427	1.99901
C	0.47393	3.64823	5.38836
H	-0.38976	3.73320	6.05812
H	1.26553	3.03177	5.83596
H	0.90290	4.66228	5.26955

Molecule: **IM3_conf_1**

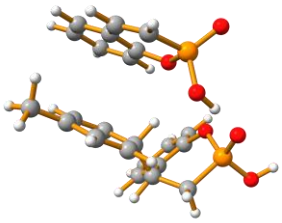

Energy = -2025.11285578 Hartree

Molecule: **IM3_conf_2**

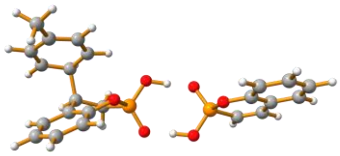

Energy = -2025.11755684 Hartree

54

C	-1.27000	11.78000	0.28300
C	-0.05700	11.22300	0.66900
C	0.11500	9.82500	0.73900
C	-0.98800	9.01000	0.40500
C	-2.21000	9.55700	0.02100
C	-2.34700	10.94300	-0.04100
H	2.16000	9.95700	1.43500
H	-1.38400	12.86300	0.23300
H	0.79000	11.86100	0.92800
C	1.37600	9.25000	1.15100
H	-3.03800	8.89000	-0.22600
H	-3.30300	11.37300	-0.34500
C	1.63800	7.92100	1.20800
H	2.61100	7.54500	1.52700
O	-0.91100	7.63200	0.46500
P	0.43100	6.74800	0.70800
O	0.79100	5.99200	-0.55500
O	0.05500	5.81500	1.91200
H	0.02600	4.79200	1.70200
C	-3.53100	-1.06500	1.16500
C	-2.27300	-1.13000	0.57000
C	-1.64100	0.02900	0.09700
C	-2.31600	1.25200	0.24100
C	-3.57700	1.33500	0.82800
C	-4.18400	0.16800	1.29000
H	-4.00500	-1.97500	1.53400
H	-1.76000	-2.08900	0.47200
C	-0.29200	-0.02200	-0.54900
H	-4.06200	2.30800	0.91600
H	-5.17000	0.22100	1.75300
C	0.63400	1.12300	-0.11300
H	1.50300	1.23200	-0.77700
O	-1.74400	2.41700	-0.26600
P	-0.16800	2.74100	0.06300
O	0.01000	3.32200	1.44100
O	0.23400	3.66400	-1.12500
H	0.46200	4.69300	-0.85500
H	1.01600	0.87000	0.88800
H	0.19700	-0.97200	-0.29700
C	-0.97100	1.09600	-2.77500
C	-0.40400	-0.12300	-2.20500
C	-2.21000	1.08300	-3.37400
C	-1.10200	-1.37300	-2.50700
H	0.67200	-0.20500	-2.45000
C	-2.89600	-0.13700	-3.51100
H	-2.65700	2.00300	-3.75000
C	-2.32700	-1.36900	-3.12100
H	-0.65000	-2.30400	-2.16100
H	-2.86900	-2.29700	-3.30000
H	-0.40300	2.02400	-2.69900
C	-4.29900	-0.13400	-4.14600
H	-4.21300	-0.28400	-5.20200
H	-4.88500	-0.92200	-3.72000
H	-4.77400	0.80600	-3.95600

Molecule: **IM4**

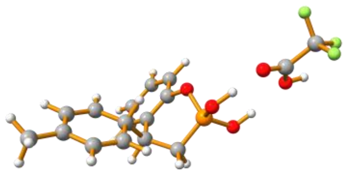

Energy = -1676.07493031 Hartree

44

C	-4.48900	-0.17100	-0.52200
C	-3.27100	-0.83900	-0.39500
C	-2.10400	-0.14000	-0.06000
C	-2.21200	1.24400	0.12500
C	-3.40800	1.93500	0.00300
C	-4.55900	1.21000	-0.31800
H	-0.82300	-1.82300	-0.35500
H	-5.38800	-0.73100	-0.78200
H	-3.21300	-1.91800	-0.55200
C	-0.76500	-0.81900	0.08800
H	-3.43200	3.01300	0.15900
H	-5.51100	1.73300	-0.41600
C	0.30800	-0.04200	-0.70300
H	0.05200	-0.09600	-1.77300
O	-1.05200	1.97800	0.49400
P	0.31600	1.69600	-0.28500
O	1.45800	2.05800	0.69600
O	0.37000	2.50200	-1.63400
H	0.59700	3.46000	-1.56200
C	2.60000	6.84300	0.55500
C	2.04300	5.42300	0.23000
F	2.23500	7.73800	-0.39400
F	3.94100	6.79500	0.61000
F	2.12500	7.25300	1.74000
O	2.23700	4.50900	1.01100
H	1.73300	3.04800	0.80700
O	1.37400	5.27300	-0.90300
H	1.32700	-0.43800	-0.59900
C	-0.39600	-1.05300	1.61700
C	-1.47900	-1.67800	2.38700
C	0.95600	-1.56200	1.88200
C	-1.22600	-2.63600	3.33800
H	-2.50000	-1.34100	2.19700
C	1.18200	-2.53200	2.82700
H	1.79900	-1.12200	1.34900
C	0.09600	-3.07300	3.54300
H	-2.04000	-3.07300	3.91600
H	2.19300	-2.88800	3.02500
H	-0.36200	-0.01900	2.06600
H	1.30200	6.11400	-1.41200
C	0.36500	-4.17500	4.58500
H	-0.36300	-4.11200	5.36700
H	0.30100	-5.13400	4.11400
H	1.34400	-4.04500	4.99700

Molecule: **IM5**

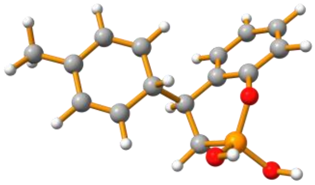

Energy = -1148.98396254 Hartree

36

C	-4.16800	-0.82800	-0.84500
C	-2.79800	-1.01600	-0.66300
C	-2.00600	-0.00300	-0.10700
C	-2.64400	1.19100	0.24200
C	-4.00200	1.41100	0.07000
C	-4.76900	0.37900	-0.47600
H	-4.77000	-1.62700	-1.27900
H	-2.32600	-1.95800	-0.95000
C	-0.52200	-0.17600	0.11800
H	-4.44100	2.36600	0.35900
H	-5.84100	0.52600	-0.61700
C	0.25600	1.05000	-0.41900
H	0.18100	1.06500	-1.51600
O	-1.86700	2.22900	0.83900
P	-0.46700	2.57400	0.16200
O	0.42700	3.18100	1.29400
O	-0.64700	3.51900	-1.07700
H	-1.08300	4.38600	-0.92300
H	1.32600	1.05300	-0.16700
H	0.13000	4.01800	1.71300
H	-0.18300	-1.04400	-0.46300
C	-0.18900	-0.52200	1.62800
C	-1.15500	-1.40200	2.29900
C	1.22700	-0.78700	1.91400
C	-0.75100	-2.34100	3.21700
H	-2.21400	-1.25800	2.08300
C	1.60600	-1.74600	2.82100
H	1.98600	-0.18000	1.41900
C	0.62300	-2.52100	3.46500
H	-1.48400	-2.95900	3.73600
H	2.66100	-1.91700	3.03700
H	-0.36600	0.43300	2.20700
C	1.06300	-3.60100	4.47000
H	0.32700	-3.69300	5.24100
H	1.16800	-4.53800	3.96400
H	2.00100	-3.32400	4.90400

Molecule: **IM6**

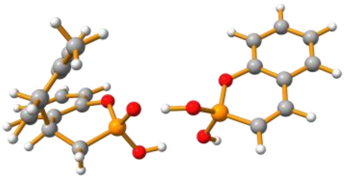

Energy = -2025.51204091 Hartree

55

C	7.24400	8.20700	-4.38200
C	7.26800	6.83900	-4.54800
C	6.24500	6.02900	-4.03400
C	5.19700	6.65600	-3.34800
C	5.17100	8.02700	-3.17500
C	6.19700	8.80300	-3.69300
H	7.15100	4.21900	-4.75800
H	8.03900	8.81300	-4.78500
H	8.08200	6.37100	-5.08000
C	6.29800	4.59800	-4.21100
H	4.35300	8.47600	-2.63400
H	6.17500	9.87300	-3.55700
C	5.38300	3.73400	-3.74800
H	5.46800	2.67000	-3.87900
O	4.12300	5.93500	-2.81600
P	4.00700	4.36700	-2.82900
O	2.59500	4.01400	-3.43500
O	3.93500	3.87400	-1.37500
H	3.04000	3.50400	-1.07600
C	-4.51400	1.57800	-0.06100
C	-3.38400	0.79000	0.03400
C	-2.11100	1.33200	-0.14900
C	-2.00800	2.69900	-0.41800
C	-3.13900	3.49400	-0.51500
C	-4.39200	2.93300	-0.33800
H	-1.20400	-0.58200	-0.25200
H	-5.48900	1.13800	0.07300
H	-3.48400	-0.26600	0.23500
C	-0.89600	0.45700	-0.09800
H	-3.01600	4.54200	-0.73400
H	-5.27300	3.54900	-0.42100
C	0.08800	0.83500	-1.20300
H	-0.38300	0.63800	-2.16900
O	-0.77400	3.32100	-0.53100
P	0.50700	2.61300	-1.16800
O	1.71400	3.02800	-0.41900
O	0.50600	3.07600	-2.67900
H	1.36300	3.33200	-3.02500
H	2.43400	4.21800	-4.36000
H	1.00700	0.25300	-1.14700
C	2.17300	-0.08500	1.85000
C	2.37600	1.21400	2.33300
C	1.33500	2.14500	2.35700
C	0.10200	1.81500	1.86300
C	-0.17800	0.48400	1.32500
C	0.95000	-0.45000	1.36400
H	2.98900	-0.79300	1.86500
C	3.74157	1.60956	2.84177
H	1.51200	3.13300	2.75500
H	-0.71000	2.52600	1.90400
H	-0.96700	0.05800	1.97100
H	0.78800	-1.45000	0.98500
H	4.42960	0.80665	2.67788
H	3.68428	1.82204	3.88889
H	4.07849	2.48027	2.31905

Molecule: **toluene**

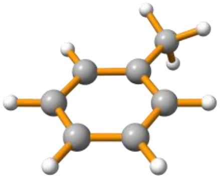

Energy = -271.70327689 Hartree

15

C	-0.38500	-0.20800	-0.00000
C	1.01400	-0.20800	0.00000
C	1.71300	1.00300	0.00000
C	1.01400	2.21400	-0.00100
C	-0.38500	2.21400	-0.00200
C	-1.08400	1.00300	-0.00100
H	-0.93100	-1.15400	0.00000
H	1.55900	-1.15400	0.00100
H	2.80500	1.00300	0.00100
H	-0.93100	3.16000	-0.00200
H	-2.17600	1.00300	-0.00200
C	1.78400	3.54800	-0.00100
H	1.96500	3.85400	-1.01000
H	1.20400	4.29500	0.50000
H	2.71700	3.42100	0.50600

Molecule: **TS1**

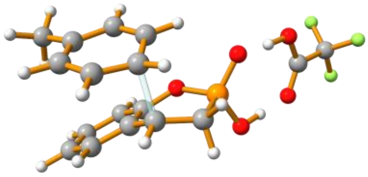

Energy = -1675.68772755 Hartree

Imag. Freq. = -433.81 cm^{-1}

43

C	-3.80000	-1.80900	-1.80600
C	-2.55400	-2.30300	-1.45100
C	-1.57700	-1.45600	-0.88100
C	-1.91400	-0.09800	-0.66800
C	-3.16300	0.40300	-1.01500
C	-4.10300	-0.45700	-1.58300
H	-0.07100	-2.97300	-0.92600
H	-4.54300	-2.46900	-2.25600
H	-2.30400	-3.35300	-1.61300
C	-0.27300	-1.97600	-0.52900
H	-3.38500	1.45500	-0.83600
H	-5.08400	-0.06700	-1.85800
C	0.92800	-1.07200	-0.53700
H	1.45600	-1.22500	-1.49400
O	-1.02000	0.75600	-0.04300
P	0.57400	0.69400	-0.38800
O	1.30700	1.42700	0.69900
O	0.75700	1.28600	-1.83400
H	1.05900	2.26800	-1.82900
C	2.70700	5.69000	-0.87600
C	2.11600	4.25100	-0.79600
F	4.02700	5.67500	-0.57600
F	2.08400	6.50600	0.00700
F	2.55800	6.20700	-2.10700
O	2.25400	3.74800	0.39400
H	1.84600	2.77700	0.48200
O	1.60500	3.74500	-1.79400
H	1.65700	-1.33800	0.24500
C	-0.49500	-2.70700	1.38800
C	-0.74100	-1.49400	2.10100
C	-1.58300	-3.61900	1.20300
C	-2.02700	-1.14400	2.45600
H	0.09600	-0.83000	2.32200
C	-2.86300	-3.26100	1.57200
H	-1.39300	-4.58800	0.74100
C	-3.11500	-2.01000	2.18100
H	-2.21900	-0.19900	2.96600
H	-3.69700	-3.94200	1.39400
H	0.52200	-3.10200	1.38500
C	-4.50900	-1.60100	2.50900
H	-4.53500	-0.86600	3.32300
H	-4.96400	-1.12600	1.62200
H	-5.13300	-2.46900	2.75900

Molecule: **TS2**

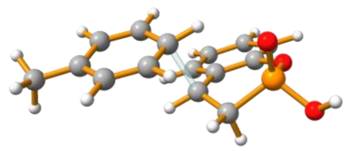

Energy = -1148.57788933 Hartree

Imag. Freq. = - 421.87 cm^{-1}

35

C	-2.46600	-1.43400	-1.02700
C	-1.08600	-1.38100	-1.15400
C	-0.36500	-0.21200	-0.81800
C	-1.09600	0.89800	-0.32600
C	-2.48100	0.84800	-0.18500
C	-3.16100	-0.31800	-0.53500
H	-3.00700	-2.33900	-1.30100
H	-0.53100	-2.24200	-1.52900
C	1.07500	-0.16400	-0.95800
H	-3.00700	1.72100	0.20200
H	-4.24500	-0.35600	-0.42600
C	1.71800	1.16200	-1.29500
H	1.61900	1.28700	-2.38700
O	-0.46000	2.04400	0.09400
P	0.95100	2.61100	-0.52400
O	1.69400	3.32800	0.54000
O	0.52300	3.47900	-1.80000
H	0.29400	4.40500	-1.57400
H	2.79300	1.18200	-1.07300
H	1.49400	-0.98400	-1.54800
C	3.64700	-2.28300	-0.13300
C	4.55400	-1.20100	-0.20700
C	2.37800	-2.09700	0.37100
C	4.20300	0.04200	0.38300
C	1.94400	-0.78100	0.73800
H	1.67200	-2.92800	0.42200
C	2.93900	0.24200	0.88100
H	4.94600	0.84000	0.41500
H	1.03200	-0.68900	1.33300
H	2.65100	1.20700	1.30200
H	3.95800	-3.26400	-0.49600
C	5.85800	-1.35600	-0.90200
H	6.63600	-0.72400	-0.45400
H	6.18300	-2.40400	-0.93600
H	5.73500	-1.01800	-1.94900

Molecule: **TS3**

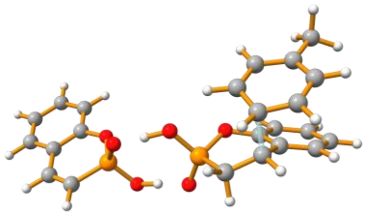

Energy = -2025.10912009 Hartree

Imag. Freq. = -313.89 cm^{-1}

C	-1.12000	11.85900	0.24600
C	0.07700	11.27400	0.64000
C	0.21400	9.87200	0.71900
C	-0.90800	9.08400	0.38800
C	-2.11500	9.65800	-0.00400
C	-2.21600	11.04800	-0.07500
H	2.26200	9.95800	1.41400
H	-1.20600	12.94500	0.18900
H	0.94000	11.89200	0.89600
C	1.45900	9.26900	1.13700
H	-2.95900	9.01200	-0.24800
H	-3.16000	11.49900	-0.38500
C	1.68800	7.93400	1.20700
H	2.65000	7.53600	1.53100
O	-0.86700	7.70500	0.45700
P	0.44800	6.78800	0.72400
O	0.79200	5.99600	-0.52200
O	0.04400	5.88700	1.94400
H	-0.03900	4.86800	1.74300
C	-3.55700	-1.26900	0.87800
C	-2.27500	-1.23000	0.34700
C	-1.66400	-0.00300	0.01100
C	-2.40900	1.18300	0.20600
C	-3.70200	1.15300	0.72400
C	-4.26900	-0.07500	1.06200
H	-4.00800	-2.22300	1.14700
H	-1.71100	-2.15200	0.19600
C	-0.29600	0.04100	-0.49100
H	-4.24300	2.09000	0.85900
H	-5.27900	-0.10100	1.47300
C	0.55200	1.22800	-0.07300
H	1.42500	1.37700	-0.72200
O	-1.89200	2.40800	-0.16600
P	-0.32400	2.80600	0.11100
O	-0.13400	3.39200	1.48400
O	-0.00800	3.74500	-1.08600
H	0.33300	4.75100	-0.81600
H	0.94100	0.98300	0.93000
H	0.24400	-0.89800	-0.34500
C	-1.00600	1.12000	-2.79400
C	-0.24400	-0.03600	-2.41000
C	-2.34900	0.98700	-3.08900
C	-0.81500	-1.33700	-2.63200
H	0.84500	0.04000	-2.47000
C	-2.92600	-0.29200	-3.11000
H	-2.96000	1.86400	-3.30200
C	-2.15200	-1.45700	-2.93500
H	-0.19500	-2.21900	-2.46200
H	-2.61700	-2.43800	-3.03300
H	-0.53000	2.10100	-2.78500
C	-4.43600	-0.43000	-3.38100
H	-4.60200	-0.52200	-4.43400
H	-4.80900	-1.30100	-2.88300
H	-4.94500	0.43600	-3.01300

Molecule: **TS4**

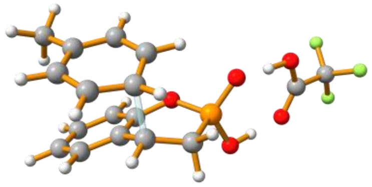

Energy = -1675.68772755 Hartree

Imag. Freq. = -433.81 cm^{-1}

43

C	-3.80000	-1.80900	-1.80600
C	-2.55400	-2.30300	-1.45100
C	-1.57700	-1.45600	-0.88100
C	-1.91400	-0.09800	-0.66800
C	-3.16300	0.40300	-1.01500
C	-4.10300	-0.45700	-1.58300
H	-0.07100	-2.97300	-0.92600
H	-4.54300	-2.46900	-2.25600
H	-2.30400	-3.35300	-1.61300
C	-0.27300	-1.97600	-0.52900
H	-3.38500	1.45500	-0.83600
H	-5.08400	-0.06700	-1.85800
C	0.92800	-1.07200	-0.53700
H	1.45600	-1.22500	-1.49400
O	-1.02000	0.75600	-0.04300
P	0.57400	0.69400	-0.38800
O	1.30700	1.42700	0.69900
O	0.75700	1.28600	-1.83400
H	1.05900	2.26800	-1.82900
C	2.70700	5.69000	-0.87600
C	2.11600	4.25100	-0.79600
F	4.02700	5.67500	-0.57600
F	2.08400	6.50600	0.00700
F	2.55800	6.20700	-2.10700
O	2.25400	3.74800	0.39400
H	1.84600	2.77700	0.48200
O	1.60500	3.74500	-1.79400
H	1.65700	-1.33800	0.24500
C	-0.49500	-2.70700	1.38800
C	-0.74100	-1.49400	2.10100
C	-1.58300	-3.61900	1.20300
C	-2.02700	-1.14400	2.45600
H	0.09600	-0.83000	2.32200
C	-2.86300	-3.26100	1.57200
H	-1.39300	-4.58800	0.74100
C	-3.11500	-2.01000	2.18100
H	-2.21900	-0.19900	2.96600
H	-3.69700	-3.94200	1.39400
H	0.52200	-3.10200	1.38500
C	-4.50900	-1.60100	2.50900
H	-4.53500	-0.86600	3.32300
H	-4.96400	-1.12600	1.62200
H	-5.13300	-2.46900	2.75900

Molecule: **TS5**

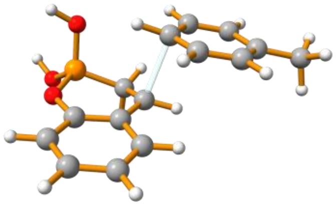

Energy = -1148.95435343 Hartree

Imag. Freq. = -300.00 cm^{-1}

C	-4.29301	-0.93519	-0.19085
C	-2.96497	-1.08523	0.11736
C	-2.07537	0.01090	0.05682
C	-2.59786	1.26253	-0.33717
C	-3.93096	1.42460	-0.63553
C	-4.77311	0.32391	-0.55565
H	-4.96268	-1.78195	-0.14978
H	-2.57395	-2.05227	0.40763
C	-0.70625	-0.18873	0.35014
H	-4.30860	2.39314	-0.93417
H	-5.82186	0.45116	-0.78791
C	0.32488	0.77267	-0.14260
H	0.49045	0.49903	-1.19766
O	-1.80806	2.37756	-0.46476
P	-0.25455	2.45079	-0.13663
O	-0.03848	3.04982	1.28454
O	0.45061	3.22429	-1.28205
H	0.46894	4.19764	-1.28322
H	1.29228	0.67963	0.34779
H	-0.44243	3.90502	1.52337
H	-0.35191	-1.20695	0.46385
C	-0.59115	-0.01493	2.69060
C	-1.35011	-1.17989	2.92309
C	0.80986	-0.09768	2.77704
C	-0.72823	-2.39176	3.10992
H	-2.43056	-1.11753	2.92964
C	1.42690	-1.31900	2.94715
H	1.40922	0.80091	2.69968
C	0.67136	-2.48652	3.10403
H	-1.32128	-3.28396	3.26643
H	2.50730	-1.37630	2.98129
H	-1.08214	0.95018	2.70961
C	1.33570	-3.81351	3.25650
H	0.83055	-4.42581	4.00307
H	1.29570	-4.36394	2.31344
H	2.38276	-3.71261	3.53500

Molecule: **TS6**

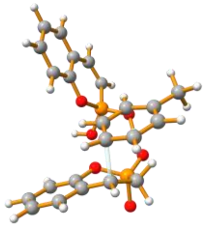

Energy = -2025.49300444 Hartree

Imag. Freq. = -368.20 cm^{-1}

55

C	5.24837	7.69586	-1.00350
C	5.71947	7.06737	-2.13925
C	5.02706	5.98994	-2.70207
C	3.84694	5.57421	-2.08284
C	3.35665	6.19621	-0.95138
C	4.06632	7.25892	-0.41402
H	6.40137	5.76933	-4.35281
H	5.79504	8.52498	-0.57668
H	6.63588	7.40030	-2.60996
C	5.51682	5.33936	-3.89572
H	2.43660	5.85518	-0.49517
H	3.69165	7.74865	0.47464
C	4.97076	4.25319	-4.47175
H	5.39239	3.80983	-5.36391
O	3.11138	4.53474	-2.59748
P	3.59061	3.50796	-3.72451
O	2.34122	3.26528	-4.64912
O	3.95540	2.13470	-3.13429
H	3.23782	1.49480	-2.74157
C	-3.40450	3.47587	-0.93724
C	-2.67843	2.64262	-0.11761
C	-1.42453	2.15968	-0.52697
C	-0.91836	2.56155	-1.77508
C	-1.63713	3.40177	-2.59822
C	-2.87941	3.85391	-2.17278
H	-1.22291	0.92568	1.20092
H	-4.37441	3.83684	-0.62643
H	-3.06871	2.34095	0.84665
C	-0.69476	1.26877	0.31885
H	-1.22971	3.70386	-3.55371
H	-3.44650	4.51233	-2.81695
C	0.30038	0.30317	-0.21656
H	-0.17574	-0.68404	-0.18951
O	0.32790	2.16900	-2.16452
P	0.84542	0.65413	-1.89800
O	2.31078	0.58726	-2.15666
O	-0.06890	-0.29334	-2.77183
H	0.15960	-0.37371	-3.71147
H	1.99702	4.03522	-5.12891
H	1.17443	0.20135	0.43559
C	2.12130	1.22051	2.54226
C	3.02306	1.61253	1.54238
C	2.62037	2.56899	0.60048
C	1.36008	3.10935	0.64740
C	0.43009	2.67538	1.61986
C	0.85987	1.76126	2.60547
H	2.43464	0.49630	3.28302
C	4.39298	1.02844	1.46977
H	3.31925	2.89059	-0.16055
H	1.06451	3.86772	-0.06569
H	-0.47539	3.24224	1.78813
H	0.17706	1.46812	3.39202
H	4.55915	0.27936	2.24054
H	5.14536	1.81112	1.58282
H	4.56134	0.56695	0.49548
